# Coumarin-facilitated iron transport: An IRT1-independent strategy for iron acquisition in *Arabidopsis thaliana*

**DOI:** 10.1016/j.xplc.2025.101431

**Published:** 2025-06-25

**Authors:** Kevin Robe, Max J.J. Stassen, Shunsuke Watanabe, Javier Espadas, Philippe Gonzalez, Alice Rossille, Meijie Li, Sonia Hem, Aurélien Roux, Véronique Santoni, Joseph Chamieh, Christian Dubos, Esther Izquierdo

**Affiliations:** 1 IPSiM, University of Montpellier, CNRS, INRAE, Institut Agro, Montpellier, France; 2 Department of Biochemistry, University of Geneva, Geneva, Switzerland; 3 IBMM, University of Montpellier, CNRS, ENSCM, Montpellier, France

**Keywords:** *Arabidopsis thaliana*, coumarins, fraxin, iron, nutrition

## Abstract

Iron (Fe) is an essential micronutrient for plant growth and development. Despite its importance, Fe uptake in alkaline soils is challenging for most plants because of its poor bioavailability. Plants have evolved two main strategies to acquire Fe. Grass species release phytosiderophores (PS) into the rhizosphere and take up Fe as Fe(III)-PS complexes via specific transporters (strategy II). Non-grass species, such as *Arabidopsis thaliana*, reduce Fe(III) to Fe(II) at the root surface and transport Fe(II) into the root via the high-affinity transporter IRT1 (strategy I). Additionally, these species secrete catechol coumarins, such as fraxetin, into the rhizosphere to enhance Fe acquisition. Although the role of catechol coumarins in Fe reduction has been clearly demonstrated in acidic soils, their functions under alkaline conditions remain unclear. In this study, we demonstrate that, at circumneutral pH, the catechol coumarin fraxetin forms stable complexes with Fe(III). We also demonstrate that fraxetin significantly improves Fe nutrition, even in mutant plants lacking IRT1 and in the presence of the strong Fe(II) chelator ferrozine, suggesting that plants can bypass the conventional Fe(II)-dependent uptake pathway. These findings support the hypothesis that Fe-coumarin complexes are taken up by plant roots in a manner analogous to Fe(III)-PS complexes in grass species, thereby challenging the current paradigm for plant Fe uptake and suggesting a more unified and flexible model in which strategy I plants can utilize Fe(III)-chelating mechanisms similar to strategy II.

## Teaser

To improve iron (Fe) acquisition, non-grass species such as *Arabidopsis thaliana* secrete catechol coumarins into the rhizosphere; however, their precise functions in circumneutral and alkaline soils, where Fe bioavailability is limited, remains unclear. This study demonstrates that secreted catechol coumarins form stable complexes with Fe(III), which are taken up by plant roots and enhance plant iron nutrition through a mechanism independent of the IRT1-mediated Fe uptake pathway.

## Introduction

Iron (Fe) is a major micronutrient required for plant growth and development, and its availability influences both crop productivity and the quality of derived products (Briat et al., 2015). Although Fe is one of the most abundant elements in soil, plants often experience Fe deficiency (Chen and Barak, 1982), particularly in alkaline soils where Fe solubility is low. In aerated soils, Fe solubility decreases by a factor of 1000 for each pH unit increase between 4 and 9 (Lindsay, 1997). The limited bioavailability of Fe in soil has driven plants to evolve strategies for Fe mobilization and uptake, known as strategy I and strategy II (Gao and Dubos, 2021; Chao and Chao, 2022; Huang et al., 2024).

Grass species (strategy II plants) release phytosiderophores from the mugineic acid (MA) family to solubilize Fe. MAs form stable complexes with both Fe(III) (ferric iron) and Fe(II) (ferrous iron). MA-Fe complexes are then taken up into the root epidermis by transporters of the YELLOW STRIPE 1 (YS1) family (Curie et al., 2001; Weber et al., 2006; Xuan et al., 2006). When expressed in the Fe uptake-deficient yeast mutant *fet3fet4*, the maize (*Zea mays*) ZmYS1 restores yeast growth in MA-containing medium, even in the presence of the Fe(II) chelator bathophenanthroline disulfonic acid (Meda et al., 2007). Intriguingly, despite the physiological importance of phytosiderophore uptake at neutral and alkaline pH, it has been shown that uptake of phytosiderophore-Fe complexes into maize roots is a pH-sensitive process. This sensitivity is attributed to the proton-coupled symporter function of ZmYS1 (Schaaf et al., 2004). It is notable that other micronutrients, such as copper (Cu), zinc (Zn), nickel (Ni), and cobalt (Co), also form complexes with phytosiderophores and are taken up in this form by the plant (Xuan et al., 2006).

In non-grass species (strategy I), efficient Fe uptake from soil is mediated by a reduction-based mechanism, at least in acidic soils (Kobayashi et al., 2012; Brumbarova et al., 2015). This process involves acidification of the rhizosphere through proton release, reduction of Fe(III) to Fe(II) by a membrane-localized reductase, and subsequent transport of Fe(II) across the plasma membrane by high-affinity Fe transporters. In *Arabidopsis thaliana*, these steps depend on the activities of AHA2 (ATPase2), FRO2 (FERRIC REDUCTION OXIDASE2), and IRT1 (IRON-REGULATED TRANSPORTER1), respectively (Robinson et al., 1999; Vert et al., 2002).

At alkaline pH, FRO2 activity is negligible (Terés et al., 2019); as a result, Fe(III) cannot be effectively reduced by FRO2, leading to low Fe uptake. To compensate for the inactivity of FRO2 under high pH, Fe-mobilizing coumarins (i.e., catechol coumarins such as fraxetin and sideretin in *Arabidopsis thaliana*) are secreted into the rhizosphere by the ABCG transporter PDR9 (PLEIOTROPIC DRUG RESISTANCE 9) (Fourcroy et al., 2014). Coumarins are specialized metabolites derived from the phenylpropanoid pathway (Rodríguez-Celma et al., 2013; Fourcroy et al., 2014; Schmidt et al., 2014; Robe et al., 2021b). In *Arabidopsis thaliana*, F6′H1 (FERULOYL-COA 6-HYDROXYLASE 1), a 2-oxoglutarate-dependent dioxygenase, catalyzes the ortho-hydroxylation of feruloyl-CoA into 6-hydroxyferuloyl-CoA, the common precursor of coumarins, which is then converted into scopoletin by COSY (COUMARIN SYNTHASE) (Rodríguez-Celma et al., 2013; Schmid et al., 2014; Vanholme et al., 2019). Because scopoletin lacks catechol groups, it cannot bind Fe and is therefore not directly involved in Fe acquisition (Rajniak et al., 2018). The two primary catechol coumarins, fraxetin and sideretin, are biosynthesized by S8H (SCOPOLETIN 8-HYDROXYLASE), a 2-oxoglutarate-dependent dioxygenase (Siwinska et al., 2018; Tsai et al., 2018), and CYP82C4, a P450-dependent monooxygenase, respectively. Both genes are upregulated in response to Fe deficiency (Murgia et al., 2011; Rajniak et al., 2018). Whereas alkaline pH favors the biosynthesis of fraxetin, the predominant catechol coumarin secreted under acidic pH is sideretin. The importance of catechol coumarin secretion for Fe nutrition has been demonstrated in multiple studies (Schmid et al., 2014; Rajniak et al., 2018; Tsai et al., 2018; Paffrath et al., 2024). There is evidence that catechol coumarins can reduce Fe(III) to Fe(II) and form stable complexes with Fe(III). However, the relative importance of each process for the plant and the precise role of these metabolites in Fe nutrition remain incompletely understood, particularly in alkaline soils where the reduction of Fe by coumarins is negligible (Paffrath et al., 2024). Three hypotheses can be proposed regarding the role of catechol coumarins in plant Fe nutrition under high pH conditions: (1) catechol coumarins form stable complexes with Fe(III) that are translocated into the roots through the activity of an unidentified mechanism at the root epidermal cells; (2) Fe(III) is reduced by catechol coumarins and directly transported by IRT1; or (3) Fe(III) solubilized by coumarins serves as a substrate for the FRO2 reductase (Rodríguez-Celma and Schmidt, 2013). These hypotheses are not mutually exclusive and may coexist, although the third appears less likely in high pH soils because FRO2 activity is negligible. The uptake of catechol coumarins by strategy I plants grown under Fe-deficient conditions has been previously reported (Robe et al., 2021a); however, the biological implications of this process require elucidation. These findings have led to the consideration of coumarins as putative siderophores, based on their strong similarities to MA.

In this study, we demonstrate that at circumneutral pH, the catechol coumarin fraxetin forms stable complexes with Fe(III). Our data indicate that Fe-coumarin complexes are taken up by plant roots and significantly enhance Fe nutrition, even in mutants lacking the high-affinity Fe(II) transporter IRT1 and in the presence of the strong Fe(II) chelator ferrozine. Remarkably, this newly identified mechanism of coumarin-mediated Fe uptake in *Arabidopsis* closely parallels the uptake of Fe-phytosiderophore complexes in grasses. Collectively, these findings support the notion that boundaries between strategy I and strategy II plants are much narrower than typically presumed.

## Results

### Coumarin uptake in the presence of iron

**Figure 1:**
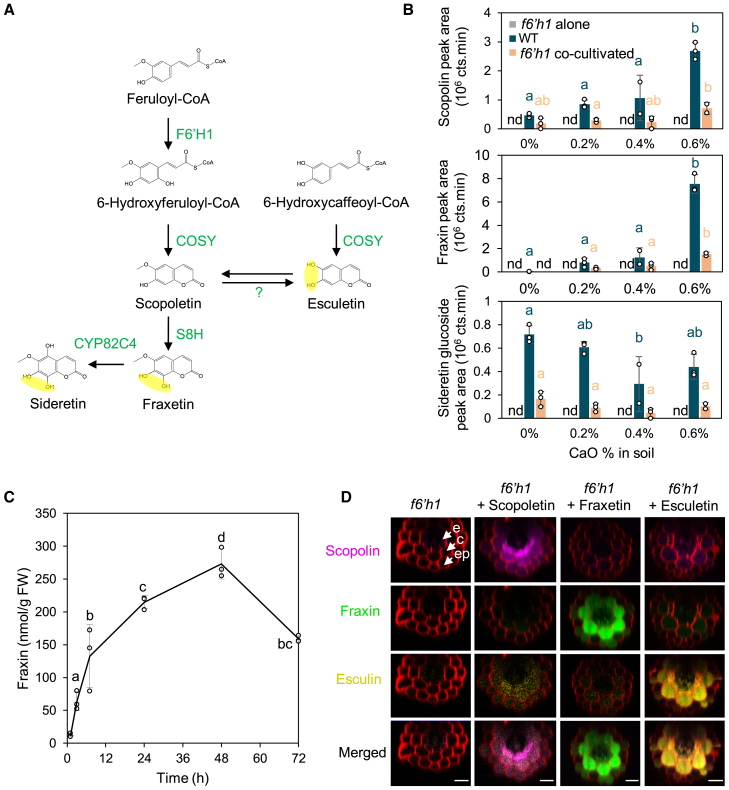
Uptake of coumarins in the presence of iron. **(A)** Coumarin biosynthetic pathway. Enzymes are indicated in uppercase green letters. Catechol groups are highlighted in yellow. Note that the conversion of 6-hydroxycaffeoyl-CoA into esculetin has only been demonstrated *in vitro*. Question marks denote putative unknown enzymes. **(B)** Scopolin, fraxin, and sideretin glucoside contents in the roots of 3-week-old *Arabidopsis thaliana* wild-type (WT) and *f6′h1* mutants, co-cultivated or not with WT plants, in soil supplemented with varying CaO concentrations (%, w/w). Coumarin quantification was performed using liquid chromatography–tandem mass spectrometry (LC–MS/MS). Coumarins were identified based on their retention times, MS, and MS/MS profiles and quantified by integrating the extracted ion chromatogram (EIC) signals of their aglycone counterparts. EIC values used for quantification were as follows: scopolin, *m/z* 193.04 ± 0.02; sideretin glycoside, *m/z* 225.03 ± 0.02; fraxin, *m/z* 209.05 ± 0.02. nd, not detected. Means within the same genotype sharing the same letter are not significantly different according to one-way analysis of variance (ANOVA) followed by post hoc Tukey test, *p* < 0.05 (*n* = 3 biological replicates of at least 20 seedlings each). Bars represent means ± standard deviation (SD). **(C)** Three-day kinetic analysis of fraxetin uptake in *f6′h1* mutants grown in the presence of poorly available Fe (25 μM FeCl_3_) and exogenous fraxetin. Root samples were collected at the indicated time points, and fraxin was analyzed by HPLC. Means sharing the same letter are not significantly different according to one-way ANOVA followed by post hoc Tukey test, *p* < 0.05 (*n* = 3 biological replicates of at least 50 seedlings each). Error bars represent means ± SD. Lines connect the means. **(D)** Localization of coumarins (scopolin, purple; fraxin, green; esculin, dark yellow) in *f6′h1* mutant seedlings grown for 3 days in the presence of synthetic scopoletin, fraxetin, or esculetin. Images were obtained through spectral deconvolution and are representative of three independent experiments, each analyzing roots from five to six seedlings. Scale bar corresponds to 25 μm. e, endodermis; c, cortex; ep, epidermis.

The uptake and glycosylation of coumarins by *Arabidopsis* roots under Fe-deficient conditions on agar plates have been previously demonstrated (Robe et al., 2021a). However, this mechanism has not been evaluated in plants grown in soil, where large amounts of insoluble iron hydroxides are expected to accumulate. Accordingly, we investigated the mechanism using *f6′h1* mutant plants grown near wild-type (WT) plants in potting soil supplemented with various concentrations of calcium oxide (CaO; i.e., 0%, 0.2%, 0.4%, and 0.6%). CaO was utilized to increase soil pH and stimulate the production of coumarins such as fraxetin and esculetin (Figure 1A; Rajniak et al., 2018). Because coumarins in roots are predominantly present in their glycosylated form, only coumarin glucosides were quantified in this tissue. Given that no commercial standards are available for sideretin glucosides, mass spectrometry (MS) was employed to unequivocally identify the coumarins produced and taken up (Figure 1B). CaO addition strongly reduced root length; thus, contamination of *f6′h1* roots by the co-cultivated WT roots can be excluded. In WT plants, scopolin and fraxin accumulation increased in a CaO concentration-dependent manner, whereas sideretin glucoside accumulation exhibited the opposite trend. As expected, none of the analyzed coumarins were detected in the roots of *f6′h1 mutants* grown alone across all tested CaO concentrations. However, MS analysis of *f6′h1* roots co-cultivated with WT plants confirmed that fraxetin and the non-catechol coumarin scopoletin were taken up by *Arabidopsis* roots grown in soil. Esculin levels in this experiment were very low and could not be quantified by MS. Sideretin glucosides were detected in *f6′h1* roots co-cultivated with WT plants but not in *f6′h1* roots grown alone, indicating that sideretin can also be taken up by plant roots (Figure 1B). To examine the kinetics of this uptake process, a time-course experiment was performed using *f6′h1* seedlings grown on control medium and then transferred to a medium containing synthetic fraxetin. Samples were collected at 1, 3, 7, 24, 48, and 72 h after transfer; fraxin accumulation was analyzed by high-performance liquid chromatography (HPLC) (Figure 1C). Fraxin was detectable in the roots as early as 1 h after transfer, although at low levels, indicating that both uptake and glycosylation, which converts fraxetin to fraxin, are rapid processes. Fraxin accumulation in the roots increased steadily over the first 48 h and began to decline 72 h after transfer. Spectral imaging was used to investigate the localization of scopolin, fraxin, and esculin after the uptake of their respective aglycone forms by *f6′h1* mutants in the presence of poorly available Fe. For this purpose, *f6′h1 mutants were* grown on a medium supplemented with 25 μM iron chloride (FeCl_3_) at pH 7 and either catechol or non-catechol coumarins. Spectral imaging confirmed that seedlings were capable of taking up coumarins in the presence of Fe, glycosylating them, and storing the glucosides in vacuoles (Figure 1D). Similar to the accumulation pattern observed under Fe deficiency (Robe et al., 2021a), fraxin and esculin accumulated intensely in the cortex and, to a lesser extent, in the epidermis and endodermis. In contrast, scopolin accumulated predominantly in the endodermis, with weaker signals in the cortex and minimal presence in the epidermis. The absence of fraxetin and esculetin in root hairs (Figure 1D) suggests that coumarins are either not taken up by trichoblasts or, if taken up, are secreted back into the rhizosphere.

### Identification and characterization of Fe-catechol coumarin complexes

**Figure 2:**
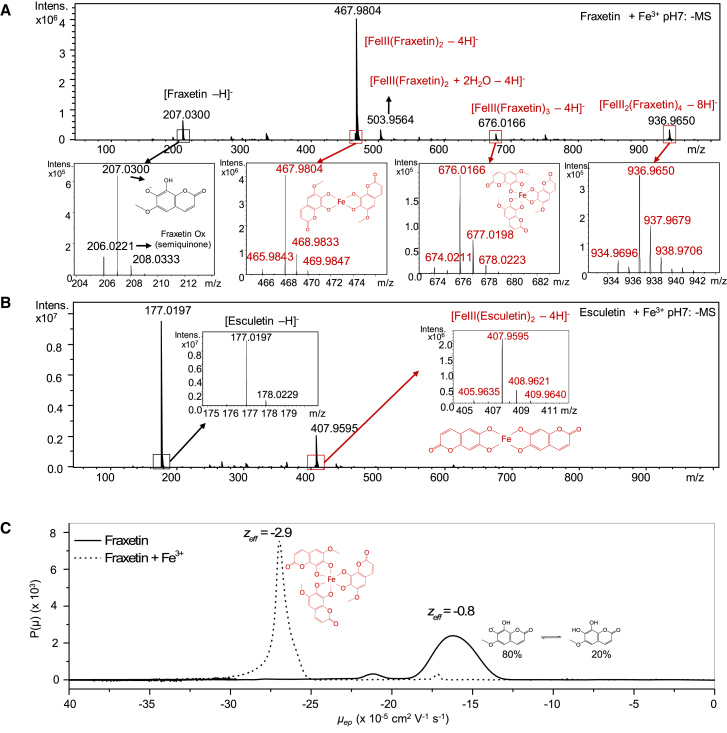
Identification of Fe-fraxetin and Fe-esculetin complexes. **(A)** Direct infusion ESI–quadrupole time-of-flight (QTOF) MS analysis of a 2:1 (mol:mol) mixture of synthetic fraxetin and Fe(III) at pH 7. **(B)** Direct infusion ESI–QTOF MS analysis of a 2:1 (mol:mol) mixture of synthetic esculetin and Fe(III) at pH 7. **(A–C)** ESI was operated in negative mode. Iron complexes were identified by their characteristic isotopic patterns and are highlighted in red. Boxes show a zoomed-in view of MS signals for the main coumarin species. MS intensity is expressed in counts. **(C)** Electrophoretic mobility distributions for fraxetin (solid line) and a 2:1 (mol:mol) mixture of fraxetin and Fe(III) (dashed line) at pH 7 in 50 mM ammonium carbonate buffer. The effective charge (z_eff_) was calculated using the model described by Ohshima (2001). The structure of the main iron-containing complex is highlighted in red. The relative abundances of protonated and deprotonated forms of fraxetin were calculated from its effective charge and align with its p*K*_*a*_ value.

Previous studies demonstrated the formation of Fe-coumarin complexes, but their characterization remained incomplete (Rajniak et al., 2018; Baune et al., 2020). To investigate Fe-coumarin complexes at circumneutral to alkaline pH, solutions of scopoletin, esculetin, and fraxetin were prepared and mixed with FeCl_3_ in an ammonium bicarbonate buffer at pH 7. Each mixture was analyzed by direct infusion in positive and negative electrospray ionization (ESI) modes. In positive ESI mode, only free coumarins ionized efficiently, and Fe complexes were barely detectable. In negative ESI mode, Fe(III) complexes with fraxetin and esculetin were identified as singly charged species (Figure 2A and 2B). Fe complexes were readily recognized by the characteristic isotopic profile of Fe (Tsednee et al., 2016); the relative abundances of naturally occurring stable Fe isotopes are approximately ^54^Fe (5.8%), ^56^Fe (91.7%), ^57^Fe (2.2%), and ^58^Fe (0.3%). When fraxetin/FeCl_3_ mixtures were infused, the predominant species detected was the 1:2 stoichiometry complex [Fe^III^(fraxetin)_2_–4H^+^]^˗^ (*m/z* = 467.99). Additional complexes, including the 1:3 [Fe^III^(fraxetin)_3_–4H^+^]^˗^ (*m/z* = 676.03) and the 2:4 [Fe^III^_2_(fraxetin)_4_–8H^+^]^˗^ (*m/z* = 936.98), were also observed (Figure 2A). Analysis of esculetin/FeCl_3_ solutions revealed the presence of [Fe^III^(esculetin)_2_–4H^+^]^˗^ (*m/z* = 407.96) (Figure 2B). No Fe complexes with scopoletin were detected. Notably, even in the absence of Fe addition, small quantities of fraxetin-Fe complexes were identified (supplemental Figure 1), most likely due to Fe contamination in the infusion system. To confirm that Fe-fraxetin complexes formed prior to introduction into the MS system, fraxetin was incubated with isotopically labeled ^57^Fe^3+^. In this experiment, the expected 1 Da MS shift was observed for species containing one Fe ([Fe^III^(fraxetin)_2_–4H^+^]^˗^ (*m/z* = 468.98) and [Fe^III^(fraxetin)_3_–4H^+^]^˗^ (*m/z* = 677.02), and a 2 Da MS shift for species containing two Fe atoms ([Fe^III^_2_(fraxetin)_4_–8H^+^]^˗^ (*m/z* = 938.97)). Additionally, the isotopic profile of all major species identified as Fe-fraxetin complexes was altered, consistent with the incorporation of ^57Fe^ (supplemental Figure 2). The decrease in free coumarin signal intensity after Fe addition was considerably higher for fraxetin (90%) than for esculetin (25%) (Figure 2A and 2B compared with supplemental Figure 1), indicating that fraxetin is a stronger Fe chelator than esculetin at circumneutral pH. Fraxetin is mostly produced in alkaline soil (Figure 1B), suggesting that Fe-fraxetin complexes exhibit greater stability under these conditions. Intriguingly, upon Fe addition, substantial amounts of oxidized free fraxetin (fraxetin semiquinone, *m/z* = 206.0221) were detected (Figure 2A); only the reduced form of fraxetin was detected (*m/z* = 207.0351) in the absence of Fe (supplemental Figure 1). This finding suggests that whereas most solubilized Fe(III) forms stable chelates with fraxetin, redox dissociation of the complex may produce a small fraction of soluble Fe^2+^ and free oxidized fraxetin.

The stabilities of Fe-fraxetin complexes at various pH values were assessed by MS (supplemental Figure 3). No significant change in the signal intensity of the complex (*m/z* = 467.99) was observed at pH 8 and 7. At pH 6, the signal slightly decreased; at pH 5, 50% of the complex was lost; and at pH 3, almost no complex remained. The loss of complex signal was accompanied by an increase in the free fraxetin signal, confirming that the Fe-fraxetin complex dissociates under acidic conditions (supplemental Figure 3).

Contrary to previous findings (Baune et al., 2020), all Fe-coumarin complexes detected in our study contained Fe(III). Singly charged species of Fe(II) complexes would carry one additional proton compared with Fe(III) complexes and would therefore be 1 Da heavier. The isotopic profiles of the 467.99 and 676.01 *m/z* peaks closely matched the theoretical profiles (supplemental Table 1), making the presence of +1 Da signals corresponding to Fe(II) complexes highly unlikely. The ESI source represents an artificial environment that induces ionization and does not reflect the actual charge states of species under physiological conditions. Moreover, ionization efficiency varies among species. To further determine whether fraxetin chelates Fe(III) or Fe(II), Fe-fraxetin complexes were analyzed using capillary electrophoresis, a milder technique that preserves molecules in their native state. Figure 2C shows the electrophoretic mobility distributions of fraxetin and a fraxetin/FeCl_3_ mixture, derived by converting the temporal experimental electropherogram at pH 7 into a mobility distribution (Chamieh et al., 2015). Fraxetin exhibited an average electrophoretic mobility (*μ*_*ep*_) of −16.42 × 10^−5^ cm^2^ s^−1^ V^−1^, whereas the Fe(III)-fraxetin complex displayed a value of *μ*_*ep*_ = −27.02 × 10^−5^ cm^2^ s^−1^ V^−1^ (Figure 2C). Using these *μ*_*ep*_ values along with the hydrodynamic radii of fraxetin (*R*_*h*_ = 0.49 nm, obtained by Taylor dispersion analysis) and the Fe(III)-fraxetin 1:3 complex (*R*_*h*_ = 0.77 nm, determined by modeling), the effective charges of both species were calculated and found to be −0.8 and −2.9 for fraxetin and the Fe(III)-fraxetin complex, respectively. The effective charge of fraxetin aligns with the p*K*_*a*_ value of 6.61 determined in the present study (supplemental Figure 4**)**. The effective charge of −2.9 for the Fe-fraxetin complex indicates that the main species at pH 7 is the 1:3 stoichiometry Fe(III)-fraxetin complex (Figure 2C and supplemental Figure 5; supplemental Table 2), in which one Fe^3+^ atom is coordinated by three fraxetin molecules, each lacking two protons, resulting in an overall charge of −3. Other stoichiometry complexes might also be present at very low concentrations. The higher MS intensity observed for the 467.98 *m/z* peak, corresponding to the 1:2 complex, may reflect superior ionization efficiency of this species and/or *in source* dissociation of the 1:3 complex.

By analogy with phytosiderophores, we explored whether coumarins could form complexes with other transition metals. In particular, we investigated interactions of fraxetin with the divalent cations Ni^2+^, Zn^2+^, Mn^2+^, and Cu^2+, as well as^ the trivalent cation Al^3+^. The identification of metal-coumarin complexes relied, as with Fe, on metal-specific isotopic signatures observed in the metal complex spectra (supplemental Table 3). Low-intensity signals corresponding to fraxetin complexes with Zn, Al, Ni, Mn, and Cu were detected, with a metal-ligand stoichiometry of 1:2 (supplemental Figure 6). However, in all cases, most fraxetin remained in free ligand form, indicating that the chelation efficiency of these metals by fraxetin is very low relative to Fe. These results demonstrate that fraxetin is a strong and highly specific Fe chelator compared with other transition metals.

### Fe-coumarin complexes can rescue *irt1* and *bhlh121* mutants defective in Fe^2+^ uptake

**Figure 3:**
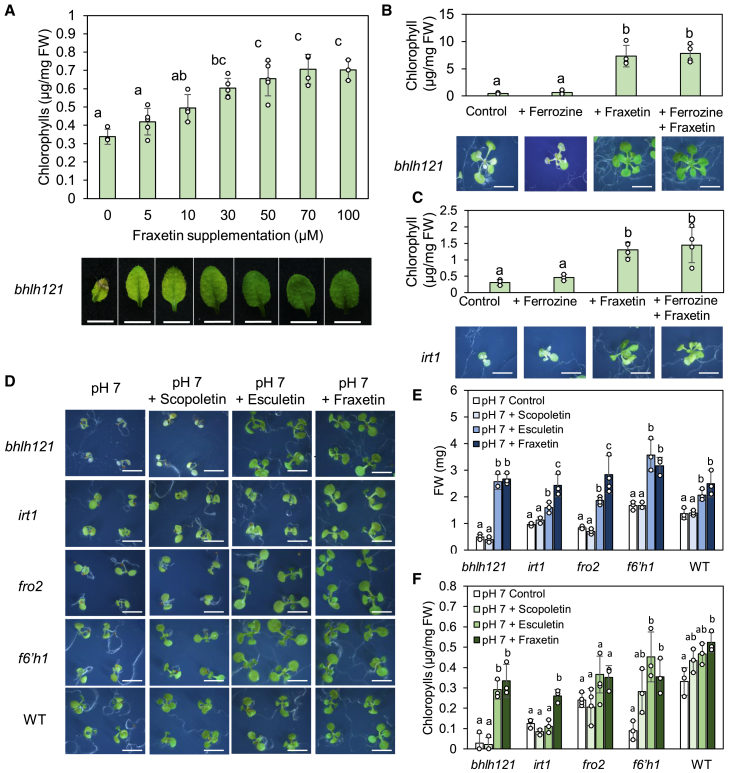
Effects of coumarins on *Arabidopsis thaliana* iron acquisition mutants. **(A)** Dose-dependent chlorophyll contents (top) and leaf phenotypes (bottom) of *bhlh121* mutants supplemented with fraxetin. Fraxetin was added at various concentrations to Hoagland medium containing poorly available Fe (25 μM FeCl_3_). **(B)** Chlorophyll contents (top) and phenotypes (bottom) of *bhlh121* mutants grown in the absence of Fe^2+^ ions. **(C)** Chlorophyll contents (top) and phenotypes (bottom) of *irt1* mutants grown in the absence of Fe^2+^ ions. **(B andC)** The strong Fe^2+^ chelator ferrozine was added to the medium at 75 μM. **(A–C)** Plants were grown for 1 week on control Hoagland agar plates, then transferred for 1 week to new agar plates containing fraxetin and/or ferrozine. **(D)** Phenotypes of several Fe acquisition mutants grown for 10 days on Hoagland medium with poorly available Fe and supplemented with scopoletin, esculetin, or fraxetin. Plants were directly sown on coumarin-supplemented medium. **(E)** Fresh weights of shoots from 10-day-old seedlings shown in **(D)**. **(F)** Chlorophyll contents of shoots from 10-day-old seedlings shown in **(D)**. **(A–C, E, and F)** Means within each genotype sharing the same letter are not significantly different according to one-way ANOVA followed by post hoc Tukey test, *p* < 0.05 (*n* = 3–4 biological replicates of at least 3 seedlings each). Bars represent means ± SD.

Previous studies showed that the growth defects of *f6′h1 mutants* could be complemented by growing WT plants in close proximity or by adding exogenous coumarins to the culture medium (Tsai et al., 2018; Vanholme et al., 2019). However, the mechanism by which coumarins complement *f6′h1 mutants remains unresolved.* The stability of Fe(III)-fraxetin complexes characterized in this study, as well as in recent work (Kang et al., 2023), supports the hypothesis that secreted catechol coumarins re-enter the root as Fe-coumarin complexes, similar to MAs in grass species. To test this hypothesis, we analyzed *irt1* and *bhlh121* mutants. The *irt1* mutant is unable to transport Fe^2+^ ions from the soil, leading to severe chlorosis (Vert et al., 2002). The recently described *bhlh121* mutant is defective in activating Fe deficiency responses, including the biosynthesis of coumarins (Kim et al., 2019; Gao et al., 2020a). Both mutants exhibit pronounced chlorosis and fail to grow properly unless supplied with high concentrations of Fe (Vert et al., 2002; Kim et al., 2019; Gao et al., 2020a). We first investigated the effects of various fraxetin concentrations on the *bhlh121* mutant phenotype. For this purpose, *bhlh121* mutants were grown for 1 week on Hoagland medium containing 75 μM Fe-EDTA, then transferred to Hoagland medium supplemented with poorly available Fe. As expected, when *bhlh121* mutants were grown in the presence of 25 μM FeCl_3_ at pH 7, the plants exhibited severe chlorosis with necrotic spots on younger leaves (Figure 3A). The addition of as little as 5 μM fraxetin to the medium partially rescued the phenotype. At this concentration, necrotic spots were no longer detectable, although the leaves remained chlorotic. Increasing the fraxetin concentration progressively alleviated the chlorotic phenotype in a dose-dependent manner. Optimal growth was observed at fraxetin concentrations ranging from 70 to 100 μM (Figure 3A and supplemental Figure 7A). Spectral imaging confirmed that fraxetin was taken up by the roots and glycosylated into fraxin in *bhlh121* mutants (supplemental Figure 7B).

Although the chlorotic phenotype of *bhlh121* was rescued by external addition of fraxetin, we could not exclude the possibility that this effect resulted from the uptake of fraxetin-reduced Fe^2+^ ions by alternative ferrous iron transport systems. To confirm that coumarins directly enter the roots bound to Fe(III), we utilized the strong Fe(II) chelator ferrozine (3-(2-pyridyl)-5,6-diphenyl-1,2,4-triazine-4′,4″-disulfonic acid) to sequester all free Fe^2+^ ions potentially reduced by fraxetin. The ability of fraxetin to rescue the Fe-deficiency phenotypes of *bhlh121 and irt1 mutants* was not diminished in the presence of ferrozine. Plants displayed phenotypes identical to those grown without ferrozine (Figure 3B and 3C). Moreover, ferrozine did not affect fraxetin uptake (supplemental Figure 8). These findings indicate that the complementation of *bhlh121-2* and *irt1* mutant phenotypes by fraxetin does not rely on Fe^2+^ ions and is most likely due to the direct uptake of Fe(III)-fraxetin complexes by plant roots.

The Fe-deficiency phenotypes of *bhlh121*, *irt1*, and other Fe acquisition mutants such as *fro2* and *f6′h1*, grown directly on Hoagland medium containing poorly available Fe, were rescued by the addition of fraxetin and, to a lesser extent, esculetin (Figure 3D–3F). In contrast, scopoletin, which lacks the catechol moiety, was unable to complement any of the mutants (Figure 3D–3F). Notably, *irt1* produces catechol coumarins under both Fe-sufficient and Fe-deficient growth conditions, unlike the *bhlh121* mutant (supplemental Figure 9).

Because ferritin accumulation serves as a proxy for Fe content in plants, western blot analysis was performed on *irt1* seedlings grown on media containing poorly available Fe and supplemented with various coumarins. *irt1* mutants accumulated very low levels of ferritins in leaves. Ferritin accumulation increased when *irt1* mutants were grown with fraxetin but not in the presence of scopoletin or esculetin, confirming that the addition of fraxetin enhanced Fe content in the symplasm (supplemental Figure 10). Considering that esculetin only partially complemented the phenotypes of *irt1* and *bhlh121* mutants and its uptake did not result in ferritin accumulation, it is likely that the primary role of this coumarin is not Fe transport into the roots. Moreover, esculetin secretion is relatively limited (Ziegler et al., 2017; Tsai et al., 2018; Terés et al., 2019); its specific function in plant Fe nutrition, if any, remains to be elucidated.

### Uptake of Fe(III)-fraxetin is not restricted to *Arabidopsis*

Several eudicots, such as tomato (*Solanum lycopersicum*), are capable of taking up fraxetin into their roots (Robe et al., 2021a). To determine whether the uptake of Fe-coumarin complexes observed in *Arabidopsis* can also rescue Fe acquisition mutants in other strategy I plants, we grew the T3238*fer* tomato mutant, which is unable to activate the Fe deficiency response, with or without fraxetin supplementation (Ling et al., 2002). After 3 weeks of growth on Hoagland medium containing 75 μM Fe-EDTA, plants were transferred to Hoagland medium containing poorly available Fe, supplemented or not with 100 μM fraxetin. One week after transfer under alkaline conditions, T3238*fer* mutants exhibited pronounced chlorosis. However, T3238*fer* mutants grown in the presence of fraxetin did not display a chlorotic phenotype (supplemental Figure 11A), strongly suggesting that Fe(III)-fraxetin complexes are also taken up in tomato*.* Furthermore, HPLC analysis of root extracts demonstrated that the T3238*fer* mutant glycosylated the absorbed fraxetin, converting it to fraxin (supplemental Figure 11B). These findings indicate that fraxetin prevents chlorosis in eudicots other than *Arabidopsis thaliana*. The presence of S8H orthologs in numerous eudicot species strongly supports the notion that the uptake of Fe(III)-fraxetin complexes is a widespread Fe acquisition strategy among eudicot plants (Rajniak et al., 2018).

### Catechol coumarin uptake is an active mechanism

**Figure 4:**
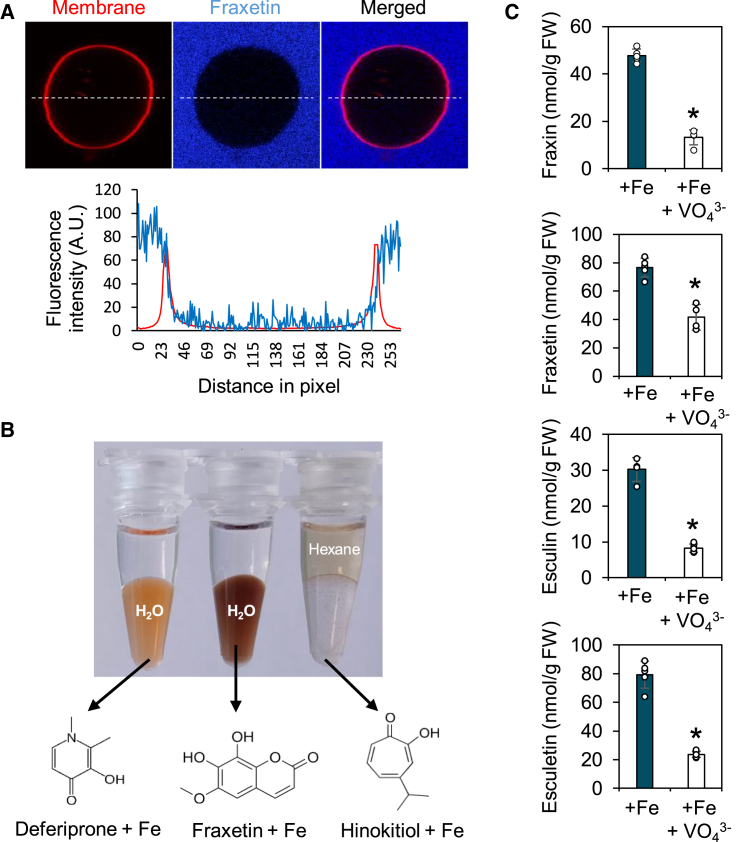
Characterization of the membrane diffusion capacity of fraxetin. **(A)** Fraxetin diffusion assay in liposomes. Fraxetin (1 mM) was incubated for 1 h with liposomes. Left panel: red, liposome membrane; blue, fraxetin. Right panel: fluorescence intensity plot of fraxetin (blue) and Atto 647N (lipid labeling, red) across the dashed line. **(B)** Phase partitioning of deferiprone, fraxetin, and hinokitiol Fe complexes into hexane (upper phase) and water (lower phase). **(C)** Inhibition of coumarin uptake by orthovanadate (VO_4_^3^^˗^). Uptake of coumarins by *f6′h1* roots grown hydroponically in Hoagland solution containing poorly available Fe (+Fe; 25 μM FeCl_3_), with or without 50 μM VO_4_^3˗^. Plants were incubated for 3 h in the presence of coumarins and orthovanadate. Coumarins were quantified by HPLC. Significant differences were determined by *t*-test: ∗*p* < 0.01 (*n* = 5 biological replicates). Bars represent means ± SD.

Several specialized metabolites, such as alkaloids, have been reported to passively diffuse into plant roots from the soil (Selmar et al., 2015). Based on their octanol-water partition coefficient (logK_ow_) values (∼1.5) (Mercer et al., 2013), the coumarins studied here are also predicted to passively diffuse across the plant plasma membrane (Limmer and Burken, 2014; Selmar et al., 2015). However, at pH 7, fraxetin is deprotonated and thus negatively charged (Figure 2C and supplemental Figure 4), which would strongly limit its diffusion capacity. Additionally, the inability of rice and maize to take up fraxetin (Robe et al., 2021a) suggests that catechol coumarin uptake is not passive but requires a specific molecular mechanism present only in strategy I plants. To test this hypothesis, we assessed fraxetin diffusion in liposomes at pH 7 via confocal microscopy. Liposomes were incubated for 1 h in the presence of 1 mM fraxetin prior to observation. We did not detect fraxetin within liposomes, indicating that fraxetin cannot diffuse through lipid membranes (Figure 4A). We next investigated fraxetin lipophilicity by comparison with Fe-chelating molecules previously described as capable of diffusing through lipid membranes (hinokitiol) or not (deferiprone) (Grillo et al., 2017). All three ligands rapidly bound Fe to form colored complexes. Unlike the membrane-permeable hinokitiol-Fe neutral complexes, the negatively charged fraxetin-Fe complexes predominantly partitioned into water rather than polar solvents (Figure 4B). A similar observation was made for Fe chelates with deferiprone, which served as a model for hydrophilic chelates, showing that both ligands (i.e., fraxetin and deferiprone) form hydrophilic complexes. This finding further supports the conclusion that, due to their biophysical properties, fraxetin-Fe complexes are unlikely to passively diffuse through lipid membranes.

We also investigated the uptake of Fe-mobilizing coumarins using sodium orthovanadate (Na_3_VO_4_), an inhibitor of plasma membrane ATPases that blocks ABC transporter activities and disrupts the formation of a proton gradient across the plasma membrane (Kang et al., 2011). For this purpose, *f6′h1* mutants were grown under hydroponic conditions and supplemented with two catechol coumarins (fraxetin and esculetin) in the presence or absence of 50 μM orthovanadate for either 3 h or 3 days. The corresponding glucosides (i.e., fraxin and esculin, respectively) were quantified by HPLC. These analyses showed that orthovanadate treatment almost completely inhibited root uptake of fraxetin and esculetin, given that fraxin and esculin contents were reduced by approximately 70% after 3 h of treatment (Figure 4C). Notably, after 3 days, almost no fraxin or esculin was detected in roots by HPLC (supplemental Figure 12), demonstrating that orthovanadate blocks the uptake of fraxetin and esculetin, rather than their glycosylation. There is evidence that orthovanadate treatment does not interfere with vacuolar loading of glycosylated coumarins (Robe et al., 2021a). After 3 h, non-glycosylated coumarins were detectable in hydroponically grown roots (Figure 4C); their levels, similar to those of the glycosylated forms, were strongly reduced by orthovanadate treatment. The active uptake of fraxetin was confirmed using the sulfonylurea glibenclamide, another inhibitor of adenosine triphosphate (ATP)-dependent transport (Forestier et al., 2003), and the proton uncoupler carbonyl cyanide *m*-chlorophenylhydrazone (CCCP) (Schaaf et al., 2004). Both inhibitors, similar to orthovanadate, suppressed fraxetin uptake (supplemental Figure 13A and 13B). These findings demonstrate that catechol coumarin uptake by *Arabidopsis* roots cannot be attributed to passive diffusion and depends on an unidentified ATP-dependent mechanism.

Due to the similarities between MA and coumarin uptake, we speculated that AtYSL (YELLOW STRIPE LIKE) transporters, the closest homologs of ZmYS1, might be involved in fraxetin uptake. Within the AtYSL family, AtYSL1, AtYSL2, and AtYSL3 are the closest homologs to ZmYS1 and thus were selected for further analysis. To test whether these AtYSL transporters mediate fraxetin-Fe uptake, we analyzed *ysl1-2*, *ysl2-1*, *ysl3-1*, and *ysl1-2 ysl3-1* mutants grown on Hoagland agar plates for 1 week before transfer to a medium containing poorly available Fe (25 μM FeCl_3_), with or without fraxetin supplementation. The amount of fraxetin taken up was then quantified by HPLC. None of the tested *ysl* mutants exhibited reduced fraxetin uptake compared with WT plants (supplemental Figure 14), suggesting that AtYSL1, AtYSL2, and AtYSL3 are not involved in fraxetin-Fe uptake, although functional redundancy among transporters could not be excluded.

### Fraxetin uptake increases iron content in plant tissues

**Figure 5:**
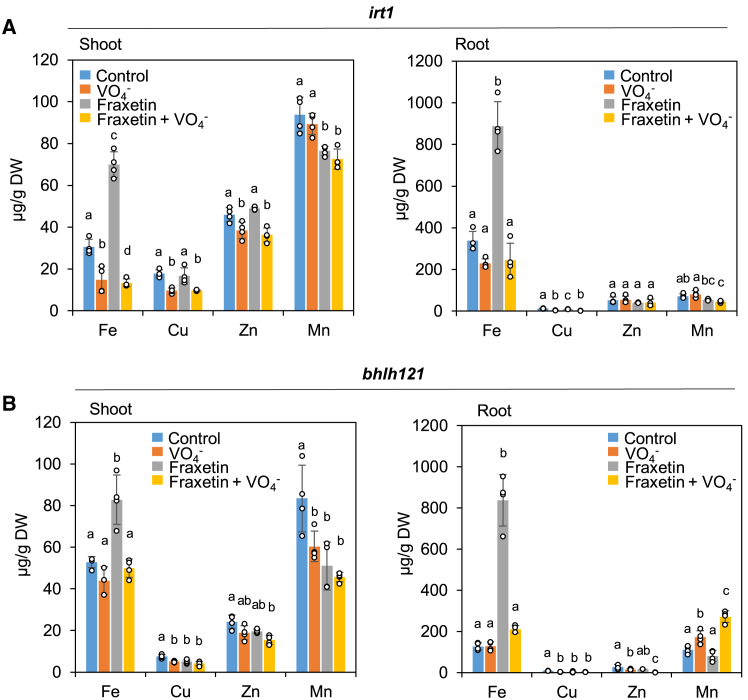
Effect of fraxetin treatment on iron accumulation in *Arabidopsis thaliana* iron acquisition mutants. **(A)** Shoot and **(B)** root Fe and transition metal contents in *irt1* and *bhlh121* mutants. Mutants were germinated and grown for 10 days on control agar plates, then transferred for another 10 days to agar plates containing poorly available Fe (25 μM FeCl_3_) supplemented or not with 100 μM fraxetin and/or 50 μM orthovanadate (VO_4_^3˗^). Means within the same metal sharing the same letter are not significantly different according to one-way ANOVA followed by post hoc Tukey test, *p* < 0.05 (*n* = 4 biological replicates of at least 20 seedlings each). Bars represent means ± SD. Fe, iron; Cu, copper; Zn, zinc; Mn, manganese.

To confirm that fraxetin is selectively taken up by roots in the form of Fe(III)-fraxetin complexes, we measured the metal contents of leaves and roots in *bhlh121* and *irt1* mutants supplemented with fraxetin. The analysis revealed that Fe content increased in both roots and shoots of *irt1* and *bhlh121* mutants grown with fraxetin (Figure 5). Notably, this Fe content increase was completely abolished when orthovanadate was also present in the culture medium. Because we previously demonstrated that fraxetin uptake is blocked by orthovanadate (Figure 4B and supplemental Figure 12), the present findings strongly suggest that an unknown ATP-dependent transport system mediates the joint uptake of Fe and fraxetin. To evaluate the contribution of Fe-fraxetin uptake to overall plant Fe acquisition, we compared the metal contents of WT plants grown with and without exogenous fraxetin. The addition of fraxetin resulted in an Fe content increase in WT plants comparable to that observed in *irt1* mutants (supplemental Figure 15A and 15B), indicating that, at circumneutral pH, Fe-fraxetin uptake plays a major role in plant Fe nutrition. Importantly, fraxetin application did not lead to increased levels of other non-ferric metals, consistent with its limited capacity to form complexes with non-ferric metals.

## Discussion

### Uptake of Fe(III)-coumarin complexes as an alternative iron uptake system

**Figure 6:**
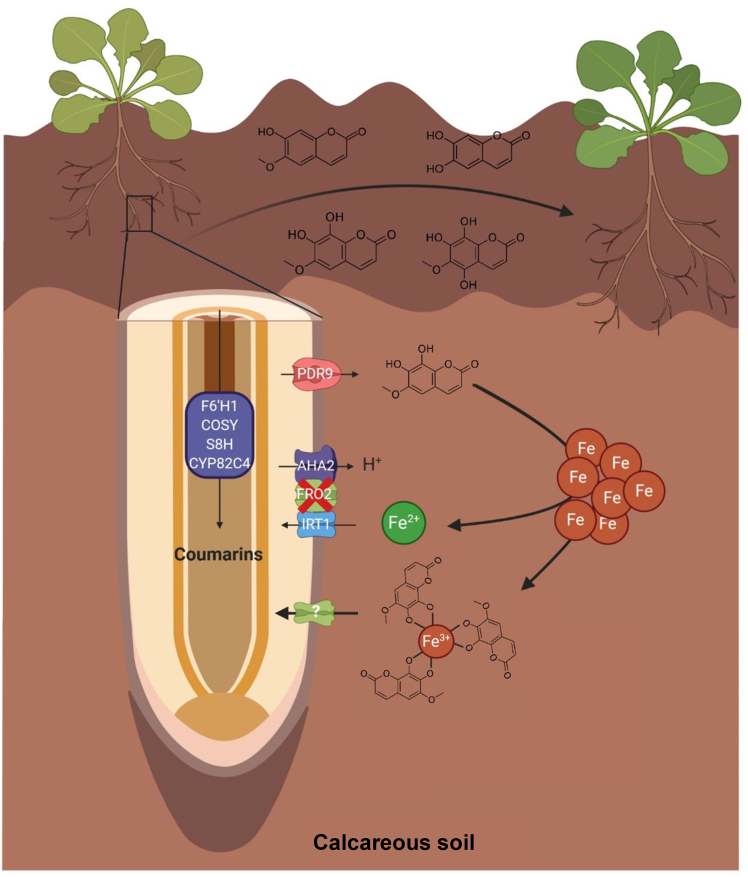
Proposed model for Fe-coumarin uptake by *Arabidopsis thaliana* roots. In calcareous or alkaline soils, the FRO2/IRT1 high-affinity Fe^2+^ transport system is nearly inactive because FRO2 activity is strongly inhibited at high pH. In response to these growth conditions, *Arabidopsis* roots produce and secrete several coumarins, including scopoletin, esculetin, fraxetin, and sideretin. Once secreted via PDR9, catechol coumarins solubilize Fe hydroxides by forming stable complexes with Fe(III) and by reducing Fe(III) to Fe(II). Reduced Fe is then taken up by IRT1, whereas Fe(III)–coumarin complexes are transported by an unidentified transporter localized in the plasma membrane of root epidermal cells. Fraxetin is the primary catechol coumarin involved in Fe(III) uptake, predominantly acting in a 3:1 fraxetin-to-Fe ratio.

Coumarins have emerged as key metabolites in iron acquisition by non-grass species (Fourcroy et al., 2014; Schmidt et al., 2014; Rajniak et al., 2018; Tsai et al., 2018); however, the mechanisms by which these compounds enhance iron nutrition have remained unclear. Several studies have shown that catechol coumarins participate in Fe reduction and/or Fe chelation (Fourcroy et al., 2016; Sisó-Terraza et al., 2016; Rajniak et al., 2018; Tsai et al., 2018; Paffrath et al., 2024). We recently demonstrated that the catechol coumarin fraxetin is taken up by several non-grass species, suggesting that this process represents a widespread phenomenon within the plant kingdom (Robe et al., 2021a). Whether this uptake is accompanied by Fe uptake has remained an open question (Rodríguez-Celma and Schmidt, 2013; Tsai et al., 2018). By complementing mutants defective in Fe(II) uptake (i.e., *irt1* and *bhlh121*) with synthetic fraxetin, we provide strong genetic evidence that *Arabidopsis* can directly take up Fe(III)-fraxetin complexes from the growth medium. Intriguingly, other Fe(III) chelates (i.e., Fe(III)-EDDHA [Fe-ethylenediamine-*N*,*N*′-bis(2-hydroxyphenylacetic acid)], and Fe(III)-HSAL [Fe-hydrothermal sulfuric acid lignin]) failed to complement such mutants (i.e., *irt1*), supporting the hypothesis that a specific uptake transport mechanism for Fe(III)-fraxetin complexes exists (Liu et al., 2023; Paffrath et al., 2024). The *fro2* mutant was also complemented by exogenous fraxetin at pH 7, demonstrating that the role of coumarins at high pH is not limited to providing chelated Fe(III) for FRO2 activity, as recently suggested (Paffrath et al., 2024). Complementation assays conducted in the presence of ferrozine confirmed this conclusion. Additionally, we showed that at pH 7, fraxetin and Fe interact *in vitro* to form highly specific and stable Fe(III)-fraxetin complexes with a 1:3 metal-to-ligand stoichiometry, similar to those observed for grass MAs. Given that both fraxetin uptake and fraxetin-mediated Fe uptake are abolished in the presence of orthovanadate, our results strongly suggest that fraxetin contributes to plant iron nutrition by forming stable Fe(III) chelates that are directly taken up by the root system (Figure 6) through a specific ATP-dependent mechanism.

### Several pathways for re-entry into the root after secretion

Although the secretion of catechol coumarins via the PDR9 transporter has been clearly established, the mechanism underlying their re-uptake remains far more elusive (Fourcroy et al., 2014). In this study, we provide evidence for a long-suspected parallel iron acquisition system in non-grass species. This IRT1/FRO2-independent system was previously proposed by Vansuyt et al. (2007), who demonstrated that iron from pyoverdine, a bacterial siderophore, is not incorporated through the classical IRT1 transporter. Similar to fraxetin-Fe complexes, several Fe-ligand complexes have been suggested to enter plants through alternative pathways. Tipton and Buell (1970) proposed that benzoxazinoids, secreted into the rhizosphere by maize roots and capable of strongly binding iron, can function as phytosiderophores. Similarly, Honda and Borthakur (2020) provided evidence that mimosine acts as a siderophore in the leguminous nitrogen-fixing tree *Leucaena leucocephala*. Notably, the peanut AhYSL1 transporter mediates the uptake of maize-derived siderophores, indicating that at least some strategy I plants can utilize strategy II siderophores (Xiong et al., 2013).

Two potential mechanisms could explain the entry of Fe-coumarins into roots: passive diffusion across the plasma membrane or active transport through a specific transporter. The negative charge of Fe-fraxetin complexes and the phase partitioning results presented here strongly argue against passive diffusion. Moreover, orthovanadate treatment substantially impaired the uptake of catechol coumarins, and fraxetin uptake was associated with increased Fe accumulation, supporting the likelihood of an active transport mechanism. ABC transporters are plausible candidates because they transport a wide variety of ions and metabolites, and they are inhibited by orthovanadate. Alternatively, the inhibition of catechol coumarin uptake by orthovanadate could result from the inhibition of plasma membrane-localized ATPases, such as AHA2, whose activity maintains the proton gradient required for transport. The inhibitory effect of CCCP on coumarin uptake further supports this hypothesis, although an indirect effect due to disrupted ATP synthesis cannot be ruled out. Overall, given the broad effects of the inhibitors used—particularly in *in vivo* systems—and their inability to exclusively block ATP-dependent processes, further studies are necessary to determine the precise molecular components responsible for Fe-coumarin complex import. Nevertheless, the data presented here strongly suggest the involvement of a proton-coupled transport mechanism for Fe-coumarin complexes, analogous to the Fe-MA uptake mediated by ZmYS1. Considering the substrates of YSL transporters (nicotianamine bound to various metals) and the inability of ZmYS1’s closest homologs to transport fraxetin, the AtYSL family seems unlikely to be involved in Fe-fraxetin uptake. AtNRAMP1 activity in *irt1* and *bhlh121* mutants could potentially explain the positive effect of fraxetin supplementation in these mutants, although the *in planta* role of AtNRAMP1 in Fe uptake has not been clearly demonstrated. Moreover, fraxetin supplementation continues to complement *irt1* and *bhlh121* mutants even in the presence of ferrozine. However, because NRAMP1 transports Fe(II), this hypothesis would require Fe(III) to be reduced by fraxetin, an activity that is negligible at neutral pH (Paffrath et al., 2024). Extensive studies will be required to identify and characterize the mechanism responsible for Fe-coumarin complex uptake. It is also probable that the uptake mechanism for non-catechol coumarins, such as scopoletin, differs from that of fraxetin and occurs independently of Fe availability.

### The advantage of a parallel iron uptake system in strategy I plants

From an ecological perspective, the presence of a chelation-based Fe(III) acquisition system in strategy I plants likely confers a strong advantage under soil conditions—such as high pH (Römheld and Marschner, 1983; Terés et al., 2019)—that inhibit Fe reduction by FRO2. This reduction step is critical for Fe acquisition via the high-affinity Fe(II) transporter IRT1 and, to a lesser extent, NRAMP1. In contrast, grasses utilize a proton-coupled, high-affinity MA-Fe transport system that enables them to thrive in high-pH soils. Proton-coupled MA-Fe transport remains functional as long as the local pH gradient generated by proton extrusion is maintained and the overall electrochemical potential difference remains negative. The data presented here support the existence of a fraxetin-Fe complex uptake system in non-grass species that is highly similar to MA-Fe uptake in grasses. This system may explain how non-grass species, including *Arabidopsis*, are able to grow in carbonate-rich soils with limited bioavailable Fe.

We have found that fraxetin, unlike MAs, has limited capacity to chelate non-ferric metals. This limitation arises because fraxetin contains a catechol moiety that strongly coordinates Fe(III) due to its high charge density, hard acid character, and preference for octahedral coordination, resulting in highly stable tris-catecholate complexes. In contrast, divalent metals such as Cu(II), Ni(II), and Mn(II), which are softer and less charged, interact more weakly with catechol moieties. Such selectivity enables fraxetin to mobilize Fe(III) efficiently without facilitating the uptake of non-target metals, which likely provides plants with an advantage in maintaining metal homeostasis under Fe-limiting conditions.

It has been shown that fraxetin secretion in 22 different *Arabidopsis* accessions is strongly correlated with plant Fe content, and accessions that secrete large amounts of fraxetin exhibit superior growth parameters (Tsai et al., 2018). Additionally, local *Arabidopsis* populations adapted to alkaline soils (pH 7.5) display high fraxin content in roots and a strong capacity for fraxetin secretion (Terés et al., 2019). In contrast, root ferric reductase activities are comparable between these populations and pH-sensitive ones (Terés et al., 2019).

The identification of a second Fe acquisition system represents a major step toward fully understanding Fe uptake in non-grass plant species and may have important implications for improving agricultural practices to prevent Fe deficiency. Selecting plants that secrete higher levels of fraxetin could significantly reduce Fe-induced chlorosis in the field without the need for synthetic Fe chelates, thereby improving cultivation and yields in soils with low Fe availability. Previous studies have demonstrated that grass species can enhance Fe nutrition in non-grass plants within intercropping systems (Brown et al., 1991; Hopkins et al., 1992; Kamal et al., 2000; Gunes et al., 2007; Xiong et al., 2013). It is therefore plausible to hypothesize that the transporter involved in Fe-coumarin complex uptake also mediates the uptake of phytosiderophores. However, previous evidence suggests that the reverse scenario is unlikely (Robe et al., 2021a).

## Materials and Methods

### Plant materials

*Arabidopsis thaliana* ecotype Columbia (Col-0) was used as the WT. *Arabidopsis thaliana* mutant lines used in this study were as follows: *f6′h1-1(*Kai et al., 2008), *irt1-2* (Varotto et al., 2002), *fro2* (*frd1-1*) (Robinson et al., 1999), *bhlh121-2* (Gao et al., 2020a, 2020b), *ysl1-2* (Le Jean et al., 2005), *ysl2-1* (DiDonato et al., 2004), *ysl3-1*, and *ysl1-2 ysl3-1* (Waters et al., 2006). The Fe-inefficient tomato (*Solanum lycopersicum*) mutant used in this study was T3238*fer* (Brown et al., 1971).

### Growth conditions

For *in vitro* culture, seeds were surface sterilized in a solution of bleach, water, and ethanol (12.5:37.5:50, v/v/v) for 5 min with vigorous agitation. Seeds were rinsed three times with 96% ethanol and allowed to dry. They were then germinated on control Hoagland medium (50 μM Fe-EDTA [pH 5.5]) solidified with 0.7% agar and grown under long-day conditions (16 h light/8 h dark).

For all experiments, control Hoagland medium contained 50 μM Fe-EDTA. Poorly available Fe Hoagland medium contained 25 μM FeCl_3_, supplemented with 0.5 g L^−1^ 4-(2-hydroxyethyl)-1-piperazineethanesulfonic acid (HEPES) buffer, and adjusted to pH 7 with KOH. For agar plates, pH was adjusted after autoclaving using pH indicator paper (±0.25 pH units).

For coumarin uptake assays, *f6′h1* mutant plants were grown hydroponically for 4 weeks in control Hoagland solution. Coumarins were then added for either 3 h or 3 days at a concentration of 50 μM in fresh Hoagland solution containing poorly available Fe (25 μM FeCl_3_ [pH 7]). For orthovanadate and glibenclamide treatments, seedlings were transferred for 3 h or 3 days into Hoagland solution containing poorly available Fe, coumarins, and either 100 μM sodium orthovanadate or 150 μM glibenclamide (both from Sigma-Aldrich).

For coumarin uptake kinetics and CCCP treatments, 1-week-old seedlings were transferred to Hoagland agar plates containing poorly available Fe and 50 μM fraxetin.

For *in vitro* mutant complementation, plants were germinated directly on Hoagland plates containing poorly available Fe (25 μM FeCl_3_ [pH 7]) supplemented with synthetic coumarins (scopoletin, fraxetin, or esculetin; all from Sigma-Aldrich). Scopoletin was supplied at 25 μM, esculetin at 50 μM, and fraxetin at 100 μM. Higher concentrations of scopoletin or esculetin (100 μM) were avoided due to observed detrimental effects on seedlings.

Tomato seeds were germinated for 1 week on moist tissue paper under long-day conditions. When cotyledons were fully developed, seedlings were transferred to Hoagland solution containing 25 μM FeCl_3_ (pH 7.5), with or without 100 μM fraxetin supplementation. Plants were subsequently grown under short-day conditions (8 h light/16 h dark).

### Coumarin imaging

Seedlings were grown for 1 week on control Hoagland agar plates (50 μM Fe-EDTA [pH 5.5]) prior to transfer for 3 days to low Fe availability medium (25 μM FeCl_3_ [pH 7]) supplemented with 25 μM scopoletin, fraxetin, or esculetin. Root cell walls were stained with 10 μg mL^−1^ propidium iodide for 10 min. Coumarins were imaged using an LSM 780 multiphoton (two-photon) microscope (Zeiss, Oberkochen, Germany) equipped with a Chameleon Ultra II laser (Coherent, Santa Clara, CA, USA) and a W Plan-Apochromat 20×/1.0 objective (Koyyappurath et al., 2015; Talamond et al., 2015). Spectral imaging was performed using laser excitation at 720 nm and the 32-channel GaAsP spectral detector, as previously described (Robe et al., 2021a). The Linear Unmixing function in Zeiss Zen microscope software (version 2.10) was used to separate the signals of the autofluorescent compounds scopolin, fraxin, esculin, and propidium iodide.

### Chlorophyll content

Chlorophyll was extracted from 2–5 mg of leaf tissue (fresh weight) in 1 mL of 100% acetone in the dark with agitation. Absorbance (A) was measured at 661.8 and 644.8 nm. Total chlorophyll content was calculated using the following equation: Chl a + Chl b = 7.05 × A_661.6_ + 18.09 × A_644.8_. Content was expressed as micrograms per gram of fresh weight. Three seedlings were pooled for each biological replicate.

### HPLC analysis of root and leaf extracts

Coumarin extraction from roots, sample preparation for HPLC, and coumarin quantification were performed as previously described (Robe et al., 2021a). HPLC–UV and HPLC–fluorescence analyses were conducted using a 1220 Infinity II LC system (Agilent Technologies) coupled to a 1260 Infinity II fluorescence detector (Agilent Technologies). Separation was carried out on an analytical HPLC column (Kinetex 2.6 μm XB-C18 100 Å, 150 × 4.6 mm; Phenomenex) with a gradient mobile phase consisting of 0.1% (v/v) formic acid in water (A) and 0.1% (v/v) formic acid in acetonitrile (B) at a flow rate of 0.5 mL min^−1^. The gradient program was as follows: 8% B for 1 min, increasing linearly to 33% B over 11.5 min, then to 50% B in 0.5 min. This proportion was maintained for 3 min before returning linearly to the initial conditions in 0.5 min. The column was equilibrated for 5 min at the starting conditions. Absorbance was monitored at λ = 338 nm; fluorescence was monitored with excitation at λ_exc_ = 365 nm and emission at λ_em_ = 460 nm.

### LC–MS/MS analysis of soil grown roots

WT and *f6′h1* plants were co-cultivated for 3 weeks in soil supplemented with 0–0.6% (w/w) CaO. Plants were sufficiently spaced to prevent root intermixing between genotypes. Soils containing *Arabidopsis* plants were rinsed roughly with tap water, and roots from both genotypes were carefully washed with distilled water to remove residual soil particles. The absence of cross-contamination between the roots of the two genotypes was verified by polymerase chain reaction genotyping (supplemental Figure 16). Coumarins were then extracted from roots as previously described for HPLC analysis (Robe et al., 2021a).

LC–MS/MS analyses were performed using an Ultimate 3000 nano HPLC system (Thermo Fisher Scientific) coupled to a quadrupole time-of-flight mass spectrometer (Maxis Impact; Bruker Daltonics), as previously described (Robe et al., 2021a). The LC conditions were identical to those used for HPLC analysis, except that the flow rate was reduced to 200 μL min^−1^. Glycosylated coumarins often lose their sugar moiety in the ESI source. Therefore, after their identities had been verified according to retention time, MS, and MS/MS profiles, fraxin, esculin, scopolin, and sideretin glycosides were quantified by integrating the extracted ion chromatogram (EIC) peaks of their aglycone counterparts. Given that no commercial standards are available for sideretin glycosides, calibration curves could not be established; thus, relative quantification was utilized for all coumarins in this experiment. Results are expressed as relative intensity peak areas (counts min^−1^).

### Direct infusion MS

To investigate the *in vitro* interactions of Fe and other transition metals with coumarins, 50 μM methanolic solutions of each ligand (fraxetin, scopoletin, and esculetin) and 50 μM aqueous solutions of each metal salt (FeCl_3_, MnCl_2_, ZnCl_2_, NiCl_2_, AlCl_3_, and CuCl_2_) were freshly prepared. ^57^Fe^3+^ was obtained from ^57^Fe_2_O_3_ (Eurisotop, Saint-Aubin, France) via overnight solubilization in 37% HCl, followed by neutralization with 1 M ammonium bicarbonate. Metal/ligand mixtures at a 1:2 ratio were prepared and diluted 1:1000 in a solution of 50% acetonitrile and 50% 25 mM ammonium bicarbonate, adjusted to pH 8, 7, 6, 5, or 3 using formic acid. The resulting solutions were injected into the mass spectrometer through a syringe pump at a flow rate of 200 μL h^−1^. Source settings were the same as those used for LC–MS analysis, except for the mass range (50–1000 *m/z*) and with ESI operated in negative mode.

### Capillary electrophoresis and Taylor dispersion analysis

Capillary electrophoresis experiments were performed using an Agilent 7100 capillary electrophoresis instrument equipped with a built-in diode array UV detector (Waldbronn). Detection was carried out at 214 nm. Fused silica capillaries (50 μm inner diameter/375 μm outer diameter) were obtained from Polymicro Technologies. The capillaries were 33.5 cm in length (25 cm to the detection window). New capillaries were conditioned with sequential washes at 1 bar: 1 M NaOH for 30 min, followed by water for 15 min. Before each sample injection, the capillary was flushed with buffer for 2 min. Samples were hydrodynamically injected at the inlet side of the capillary by applying 50 mbar pressure for 3 s. Separations were conducted under a −15 kV voltage. Each injection was repeated three times, with a 30-s buffer flush between repetitions. Samples were prepared at a concentration of 1 μM fraxetin in 50 mM ammonium bicarbonate (NH_4_HCO_3_) buffer adjusted to various pH levels (5–8) with formic acid, or in 50 mM formic acid solution at pH 2.5.

The hydrodynamic radius of fraxetin was determined via Taylor dispersion analysis (Chamieh and Cottet, 2014). Briefly, using the same capillary, instrument, and buffers as in the capillary electrophoresis experiments, samples were hydrodynamically injected by applying a 50 mbar pressure for 5 s and mobilized with the buffer under 100 mbar pressure. Elution profiles were analyzed as previously described (Chamieh and Cottet, 2014) to calculate the diffusion coefficient and determine the hydrodynamic radius (*R*_*h*_). For Fe(III)–fraxetin complexes, *R*_*h*_ was estimated from the diffusion coefficient using HydroPro software (Ortega et al., 2011) and structures drawn in Marvin Sketch (ChemAxon).

The effective charges of fraxetin and Fe-fraxetin complexes were determined using a method based on the O’Brien-White-Ohshima model (Ohshima, 2001; Ibrahim et al., 2013). Detailed protocols and equations for determining the effective charge of fraxetin and Fe(III)–fraxetin complexes, as well as the pKa of fraxetin, are provided in supplemental Methods 1 (supplemental Figure 17).

### Fraxetin diffusion in liposomes

Giant unilamellar vesicles were prepared as previously described (Velasco-Olmo et al., 2019). Dioleoyl-phosphatidylcholine, dioleoyl-phosphatidylserine, and dioleoyl-phosphoethanolamine labeled with Atto 647N (Atto 647N DOPE) lipid stocks dissolved in chloroform were mixed at 59.9:40:0.1 molar percentages, respectively, in a glass vial and dried under vacuum for 2–3 h. The dried lipid film was then hydrated in a buffer containing 25 mM HEPES (pH 7.4) to form multilamellar vesicles (MLVs) at a final concentration of 1 g L^−1^. Twenty microliters of the MLV solution were mixed with 2 μL of silica beads (40 μm in diameter) (Microspheres-Nanospheres) and deposited on parafilm. The MLV–silica bead mixture was dried under vacuum for 2 h to form supported bilayers on the silica beads. The supported silica beads were collected using a glass capillary and placed in a hydration chamber containing 1 M trehalose at 60°C for 15 min as a membrane pre-hydration step. The pre-hydrated model membranes were transferred to a 1.5-mL tube containing the working buffer (25 mM HEPES [pH 7.4]) and further hydrated for 20 min. The mixture was gently stirred for 20–30 s to detach the hydrated model membranes from the supporting silica beads. The supernatant, containing detached giant unilamellar vesicles, was transferred to a new tube containing fraxetin dissolved in the working buffer at a final concentration of 1 mM. After 20 min of incubation, samples were imaged using a scanning confocal microscope (inverted Nikon Eclipse Ti A1R microscope with a ×100, 1.3 NA oil immersion objective [Nikon]).

### Lipophilicity determination

To investigate hexane–water partitioning of iron chelates, 40 μM solutions of each ligand (fraxetin, deferiprone, and hinokitiol; Grillo et al., 2017) and 13 μM FeCl_3_ were prepared in 25 mM carbonate buffer (pH 8). The solutions were mixed with an equal volume of hexane, shaken for 5 min, and centrifuged for 5 min to separate the phases.

### Metals measurements

Approximately 2.5 mg of dry root tissue or 7.5 mg of dry shoot tissue per sample were mixed with 750 μL of nitric acid (65% [v/v]) and 250 μL of hydrogen peroxide (30% [v/v]). After incubation overnight at room temperature, samples were mineralized at 85°C for 24 h. Following mineralization, 4 mL of Milli-Q water were added to each sample. Metal contents were then measured using microwave plasma atomic emission spectroscopy (MP-AES; Agilent Technologies). Approximately 100 seedlings were pooled for each biological replicate.

### Western blot analysis

Western blotting was performed as previously described (Robe et al., 2021a). Immunodetection of ferritins was carried out using a rabbit polyclonal antiserum raised against purified FER1 (Dellagi et al., 2005). The antibody dilutions used were 1:10 000 for rabbit anti-ferritin primary antibody and 1:10 000 for horseradish peroxidase-conjugated anti-rabbit secondary antibody (Promega).

## Accession numbers

Sequence data from this article can be found in the GenBank/EMBL databases under the following accession numbers: *bHLH121* (AT3G19860), *FRO2* (AT1G01580), *F6′H1* (AT3G13610), *IRT1* (AT4G19690), *YSL1* (AT4G24120), *YSL2* (AT5G24380), and *YSL3* (AT5G53550).

## Funding

We thank Sandrine Chay (IPSiM, SAME platform, Montpellier, France) for technical support for plant Fe determination, Dr. Marie Lopez (IBMM, Montpellier, France) for help with coumarin chemistry, and Dr. Brigitte Touraine (IPSiM) for help with HPLC analysis of coumarins. We also thank Dr. Catherine Curie (IPSiM) for kindly providing seeds for *ysl* mutants. We thank the imaging facility MRI, a member of the national infrastructure France-BioImaging, supported by the 10.13039/501100001665French National Research Agency (ANR-10-INBS-04, “Investments for the Future”). Support for this work was provided to C.D. by the 10.13039/501100001665Agence Nationale de la Recherche (ANR-17-CE20-0008 and ANR-22-CE20-0006) and the National Research Institute for Agriculture, Food and the Environment (INRAE, BAP Department). K.R. was supported by a fellowship from the 10.13039/501100001665Agence Nationale de la Recherche (ANR-17-CE20-0008) and the BAP Department of 10.13039/501100022077INRAE. A.R. was supported by a fellowship from the 10.13039/501100001665Agence Nationale de la Recherche (ANR-22-CE20-0006) and the BAP department of INRAE. M.L. was supported by the 10.13039/501100004543China Scholarship Council, and S.W. by a Marie Skłodowska-Curie Individual Fellowship in Horizon (2020) from the European Council (MSCA-IF-2020). A.R. acknowledges funding from the Swiss National Fund for Research (nos. CRSII5_189996 and 310030_200793) and the 10.13039/501100000781European Research Council Synergy Grant (no. 951324-R2-TENSION). J.E. acknowledges a long-term EMBO fellowship (EMBO ALTF 989-2022).

## Acknowledgments

No conflict of interest declared.

## Author contributions

K.R., J.C., C.D., and E.I. conceived and designed the experiments. K.R., M.J.J.S., S.W., P.G., A.R., M.L., J.C., and E.I. performed the experiments. K.R., M.J.J.S., S.W., J.E., P.G., A.R., M.L., S.H., J.C., C.D., and E.I. analyzed the data. S.H. and V.S. provided access to MS facilities. K.R., J.C., C.D., and E.I. wrote the paper with the help of all the authors.

## Supplemental information

Document S1. Supplemental Figures 1–17, supplemental Tables 1–3, and supplemental Methods 1

Document S2. Article plus supplemental information
