# Supplementary material for: Coumarin-facilitated iron transport: An IRT1-independent strategy for iron acquisition in Arabidopsis thaliana
Source: Plant Commun. 2025 Jun 25;6(9):101431. doi: 10.1016/j.xplc.2025.101431 (PMC12447440; doi:10.1016/j.xplc.2025.101431)
Supplement: Document S1. Supplemental Figures 1–17, supplemental Tables 1–3, and supplemental Methods 1 [file mmc1.pdf]

Plant Communications, Volume *6*

Supplemental information

Coumarin-facilitated iron transport: An IRT1-independent strategy for iron acquisition in *Arabidopsis thaliana*

Kevin Robe, Max J.J. Stassen, Shunsuke Watanabe, Javier Espadas, Philippe Gonzalez, Alice Rossille, Meijie Li, Sonia Hem, Aurélien Roux, Véronique Santoni, Joseph Chamieh, Christian Dubos, and Esther Izquierdo

Supplemental information

Supplemental items

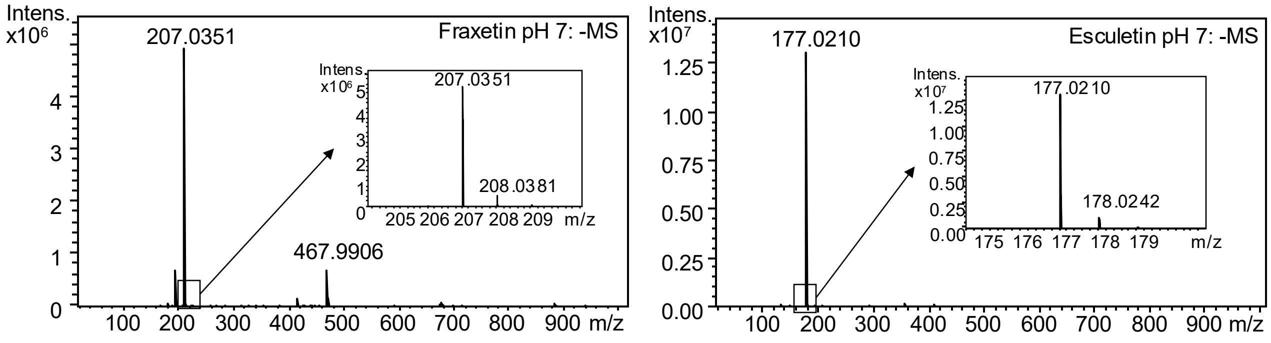

Figure S1. Direct infusion ESI-QTOF MS analysis of fraxetin (left) and esculetin (right) at pH 7 without any Fe addition. Note that [Fe(fraxetin)$_2$-4H$^+$]$^-$ can be detected even in the absence of an external Fe source, certainly due to Fe contamination.

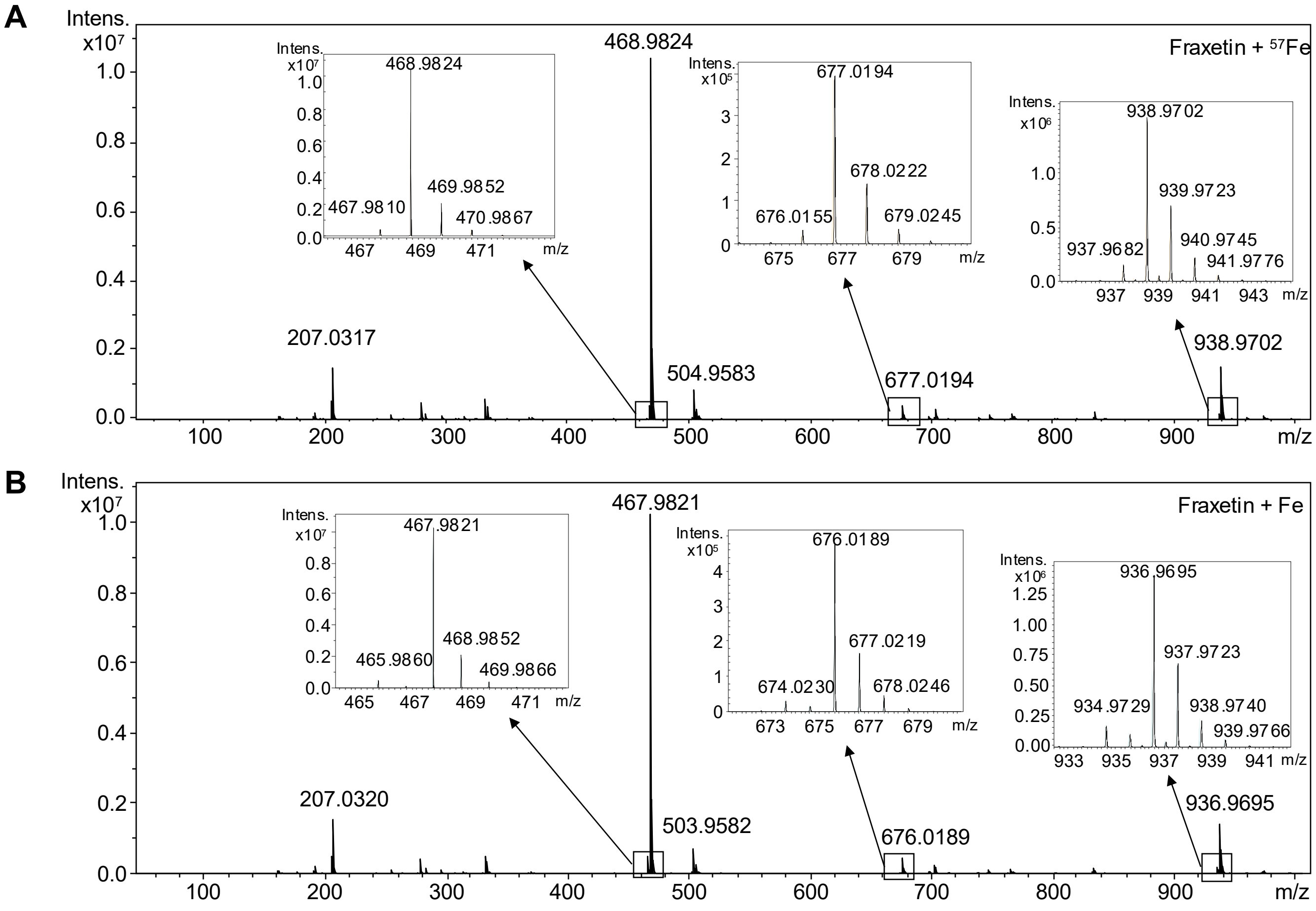

Figure S2. Analysis of isotopically labelled ^{57}Fe-fraxetin complexes.
(A) Direct infusion ESI-QTOF MS analysis of a 2:1 (mol:mol) mixture of synthetic fraxetin and $^{57}Fe^{3+}$ at pH 7. **(B)** Direct infusion ESI-QTOF MS analysis of a 2:1 (mol:mol) mixture of synthetic fraxetin and Fe^{3+} at pH 7. $^{57}Fe^{3+}$ and Fe^{3+} were obtained by acid hydrolysis of respectively $^{57}Fe_2O_3$ and Fe_2O_3. Black boxes show a zoom on the MS signal of the main coumarin species. MS intensity was measured in counts. All spectra were obtained in negative ESI mode with Q-TOF Maxis Impact HD (Bruker Daltonics). MS intensity was measured in counts.

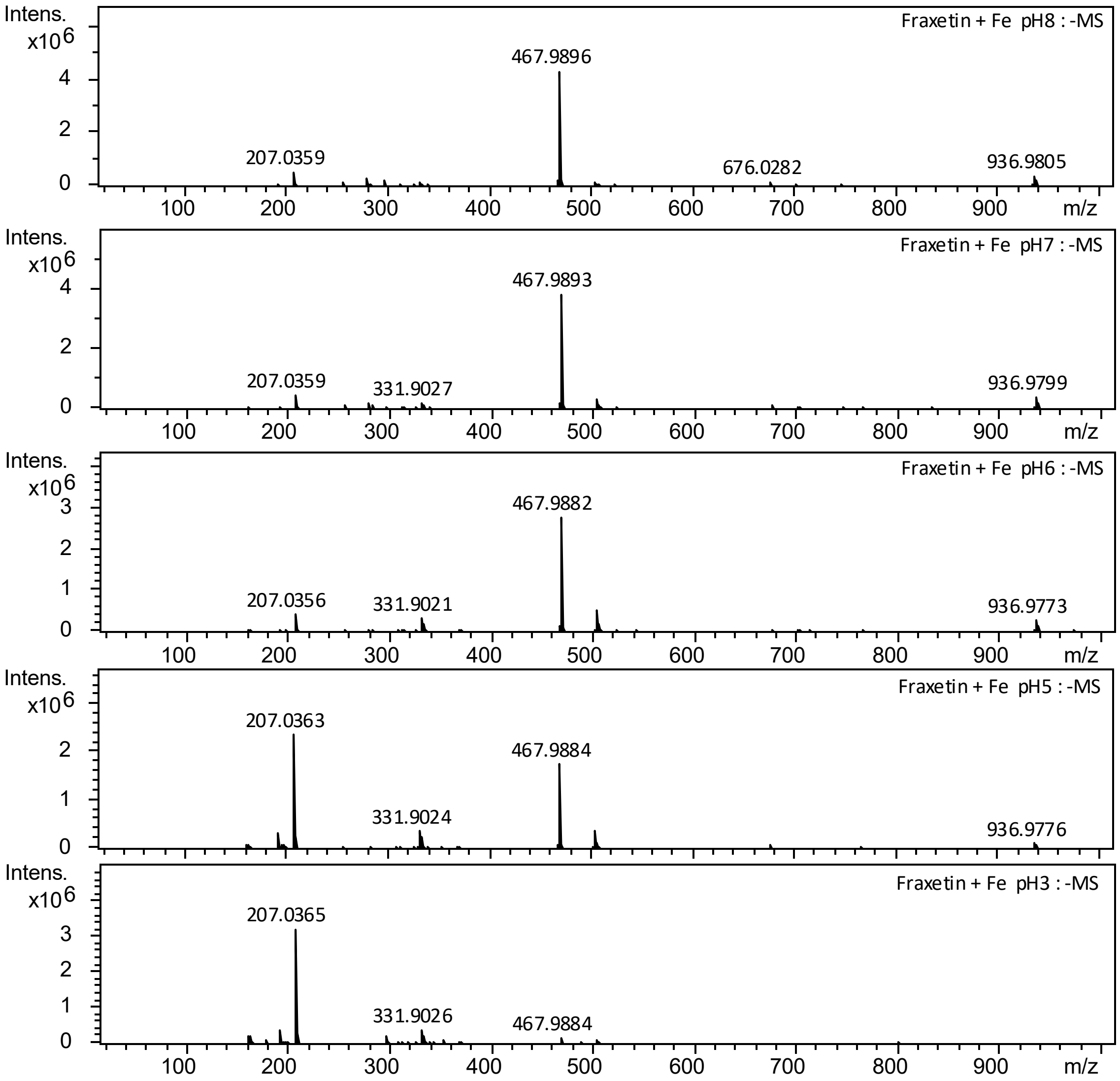

Figure S3. Analysis of the stability of Fe-fraxetin complexes at different pH. Direct infusion ESI-QTOF MS analysis. Stability of the complexes decreases when pH becomes more acidic. MS intensity was measured in counts. Note that free fraxetin signal increases in acidic conditions, confirming the dissociation of the complexes.

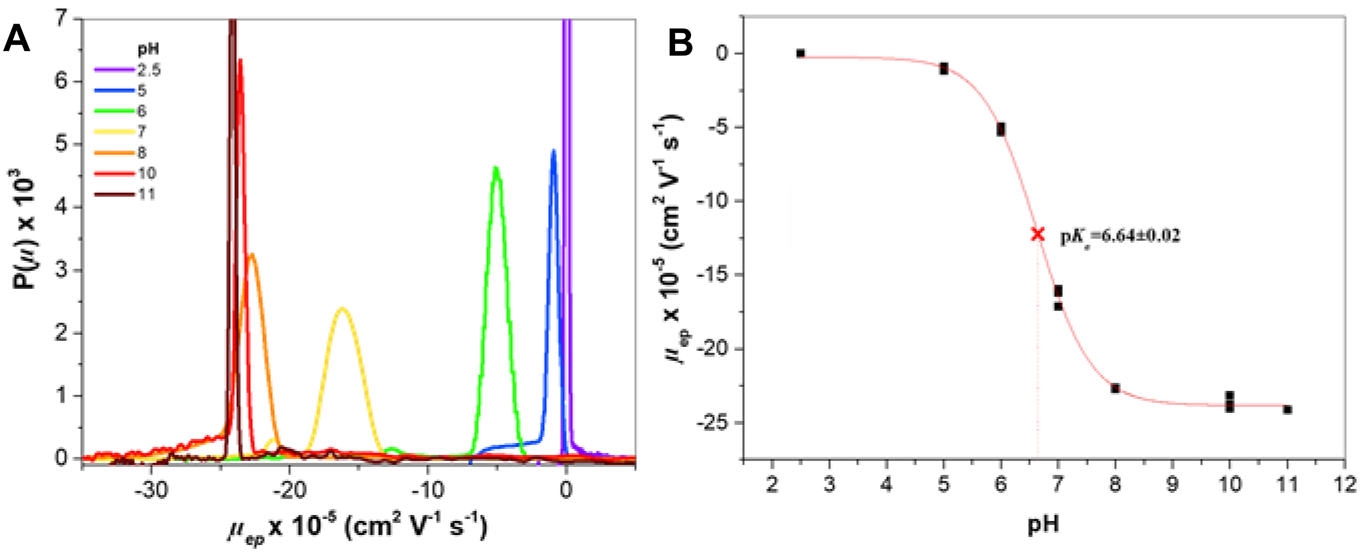

Figure S4. Determination of the pK_a of fraxetin by capillary electrophoresis.
(A) Electrophoretic mobility distributions for fraxetin as a function of pH (n=3). **(B)** Evolution of the effective mobility of fraxetin as function of pH allowing the estimation of the pK_a using equation (1) in Supplemental Method 1. Red cross represents the graphical determination of p*Ka*

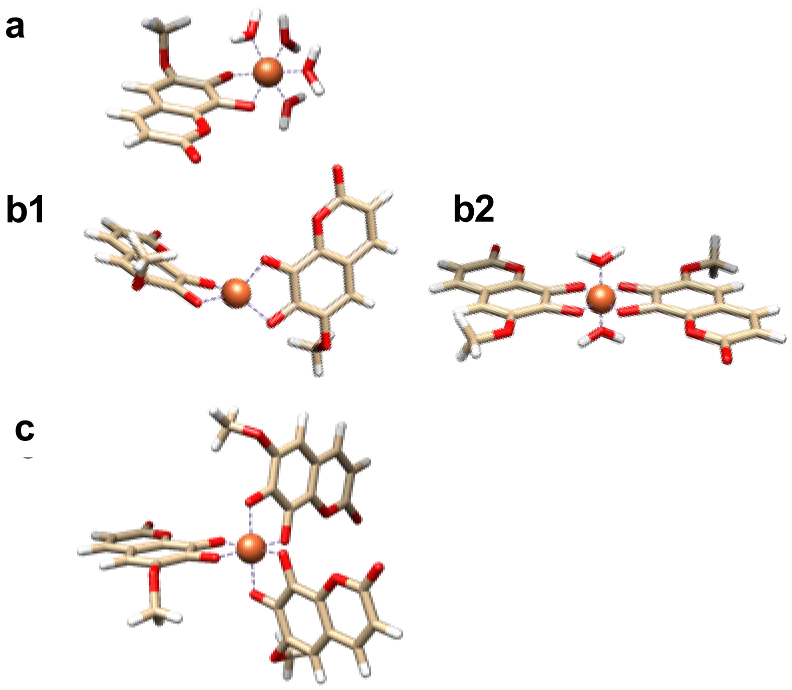

Figure S5. Proposed structures of Fe-fraxetin complexes. **a**: Fe:Fraxetin 1:1 complex, **b1**: Fe:Fraxetin 1:2 tetrahedral complex, **b2**: Fe:Fraxetin 1:2 octahedral complex; **c**: Fe:Fraxetin 1:3 octahedral complex. When needed the coordination sphere was completed with water molecules.

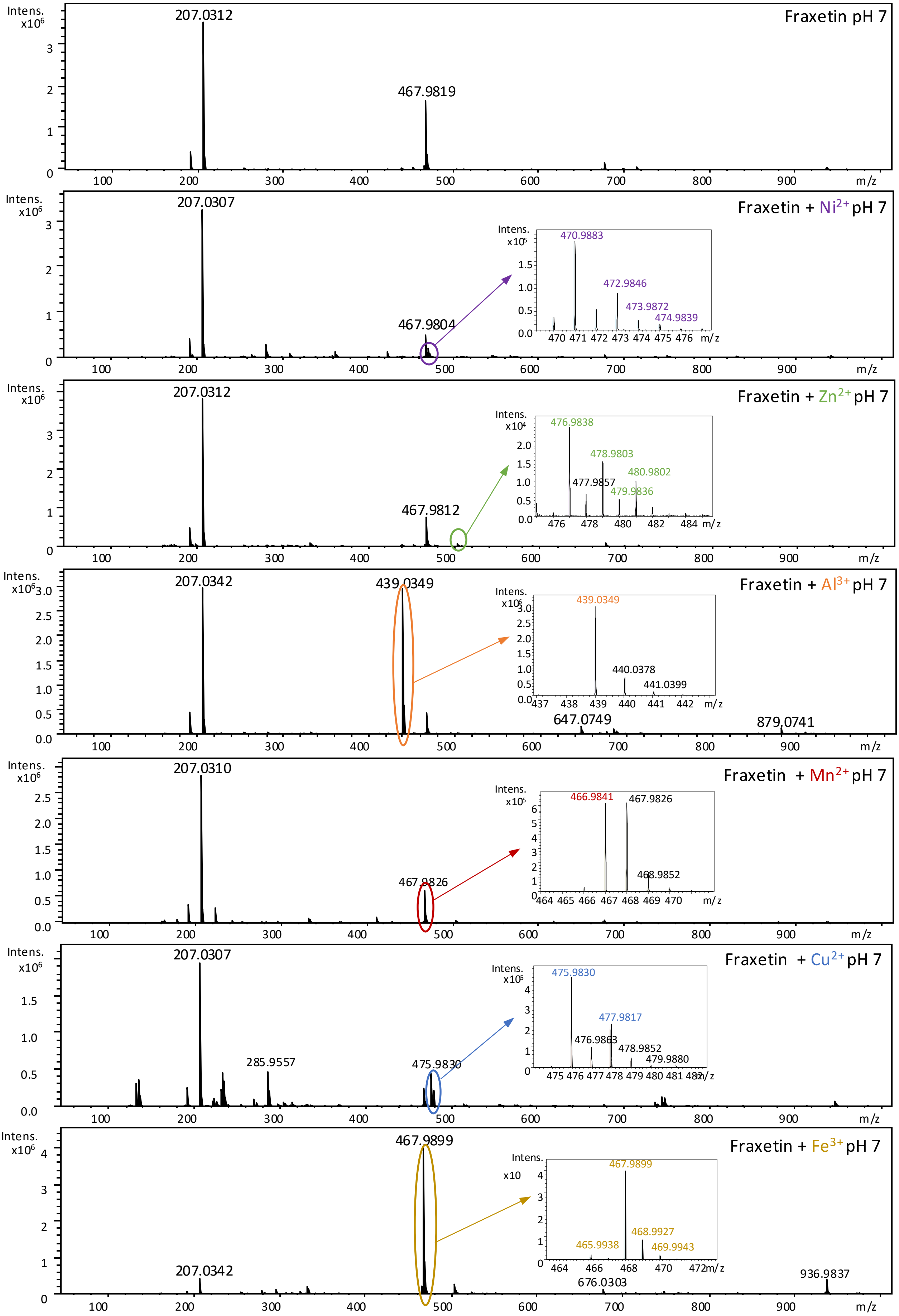

Figure S6. Identification of non-ferric metals and iron complexes with fraxetin.
Direct infusion ESI-QTOF MS analysis of a mixture of individual transition metals and synthetic fraxetin at pH 7 are presented. All tested metal solutions were prepared from chloride salts (ie: $NiCl_2$, $AlCl_3$, $MnCl_2$, $ZnCl_2$, $CuCl_2$ and $FeCl_3$). Color ellipses show a zoom of the MS signal of the formed metal-fraxetin complexes. Isotopic signatures of each metal are highlighted in colour. All spectra were obtained in negative ESI mode with Q-TOF Maxis Impact HD (Bruker Daltonics). MS intensity was measured in counts

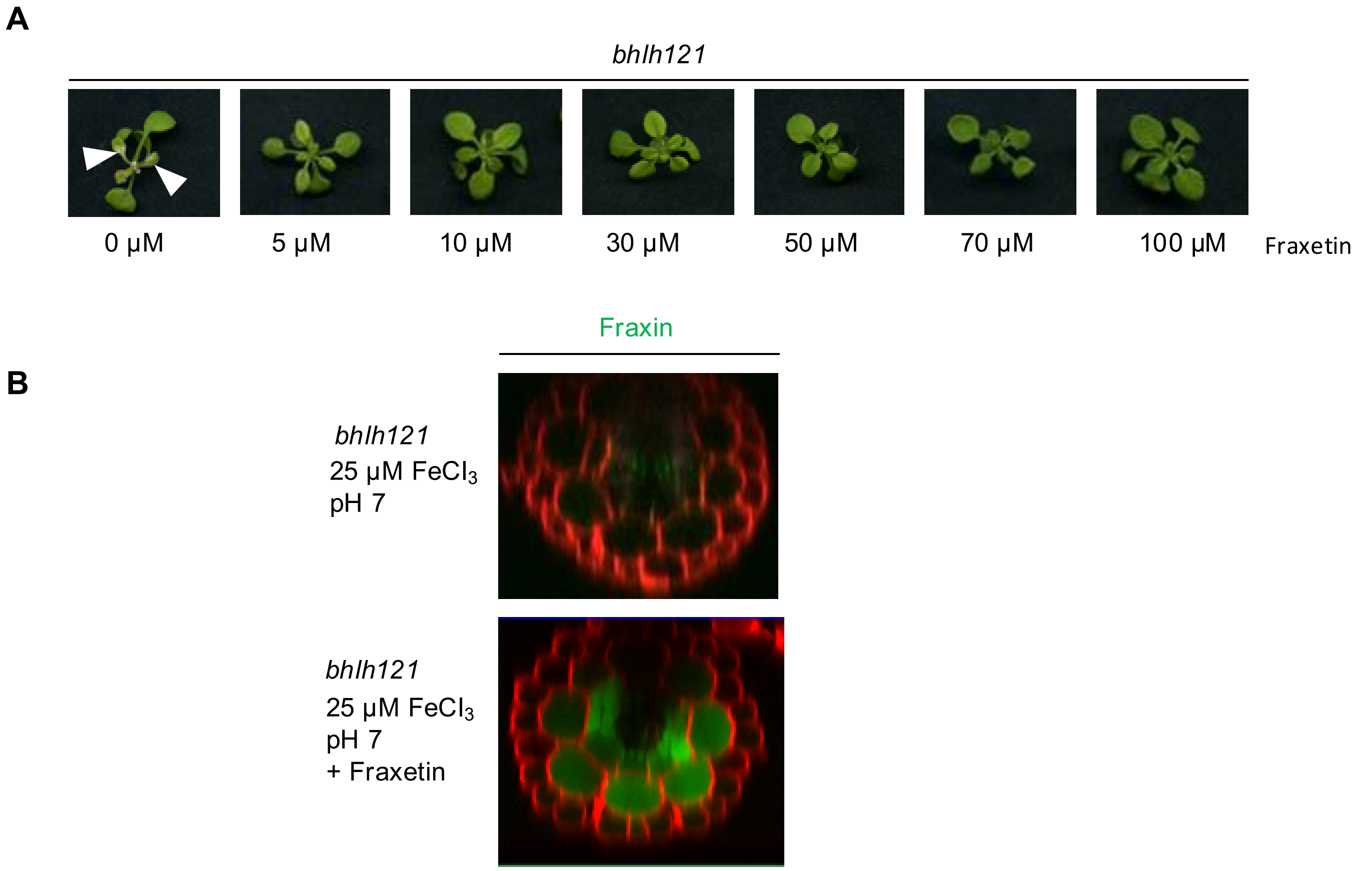

Figure S7. Phenotypic effect of fraxetin application on *bhlh121* mutant.
(A) Dose dependent phenotype complementation of *bhlh121* mutant grown with fraxetin. Fraxetin was added at different concentrations in Hoagland medium containing 25 μM $FeCl_3$ at pH 7. **(B)** Spectral deconvolution image of fraxin (green) and PI (red) in *bhlh121* mutant grown in the presence of fraxetin.

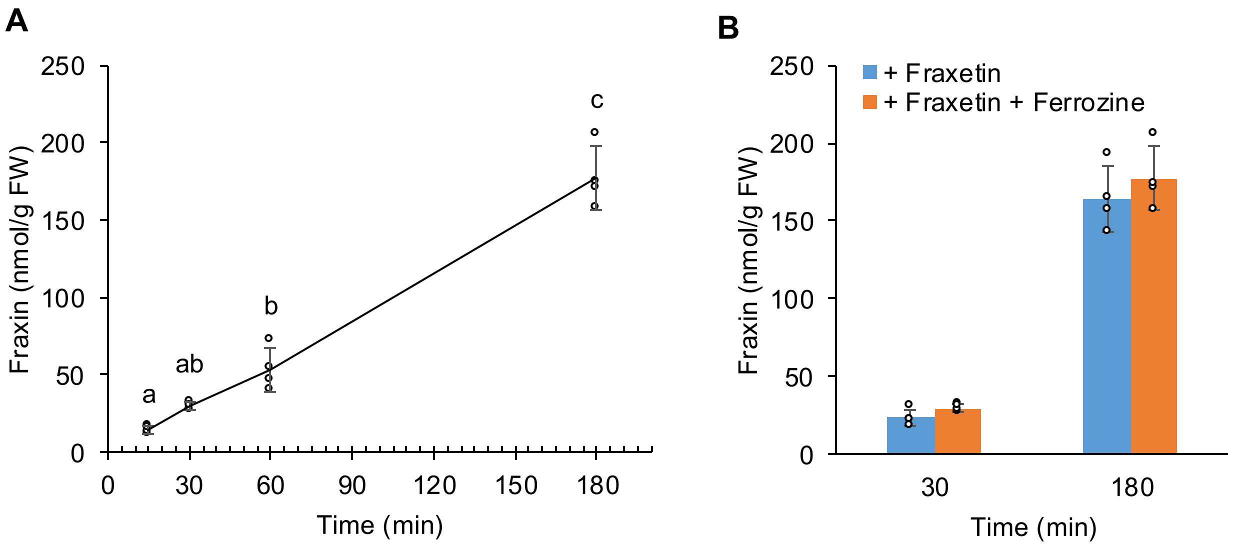

Figure S8. Fraxetin uptake in the presence of ferrozine.
(A) Three hours kinetic analysis of fraxetin uptake in *f6'h1* mutant grown in Hoagland plates containing 25 μM $FeCl_3$ at pH 7, 75μM ferrozine and 50 μM exogenous fraxetin. Time course root samples were collected and fraxin was analysed by HPLC. Means with the same letter are not significantly different according to one-way ANOVA followed by post hoc Tukey test, $P < 0.05$ (n = 4 pools of at least 50 seedlings each). Black lines are used to connect the means **(B)** Comparison of fraxetin uptake in the presence and in the absence of ferrozine at two time points (30 min and 180 min) of the kinetic study showed in (A). t test significant difference: * $P < 0.05$ (n = 4 biological repeats).

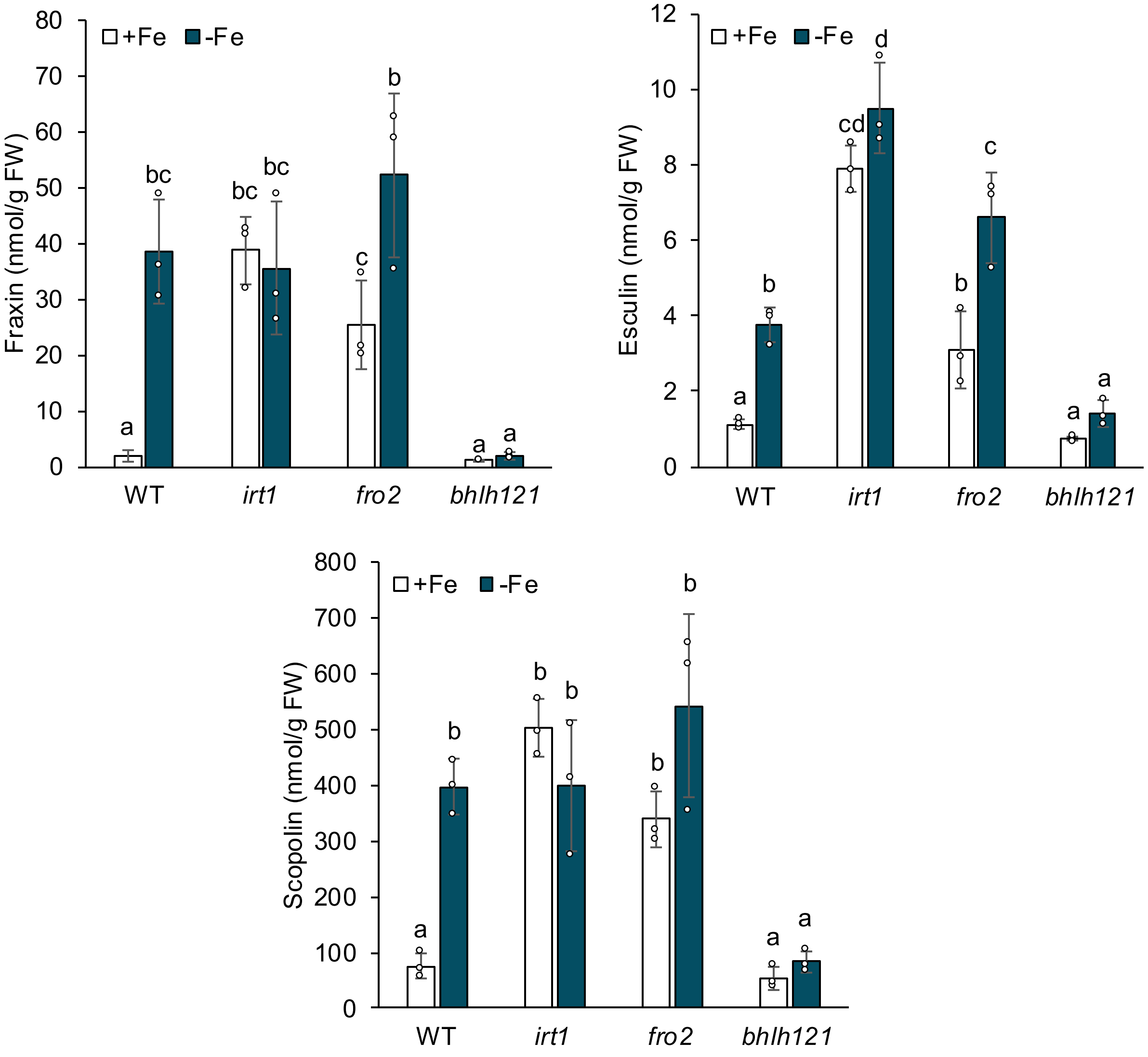

Figure S9. Coumarins accumulation in roots of iron acquisition mutants.
Analysis of fraxin, esculin and scopolin in roots of plants grown in Fe sufficient conditions (50 µM Fe-EDTA, pH 5.5) and Fe deficient conditions (0 µM Fe, pH 7). Means with the same letter are not significantly different according to one-way ANOVA followed by post hoc Tukey test, $P < 0.05$ ($n = 3$ biological repeats). Bars represent means ± SD.

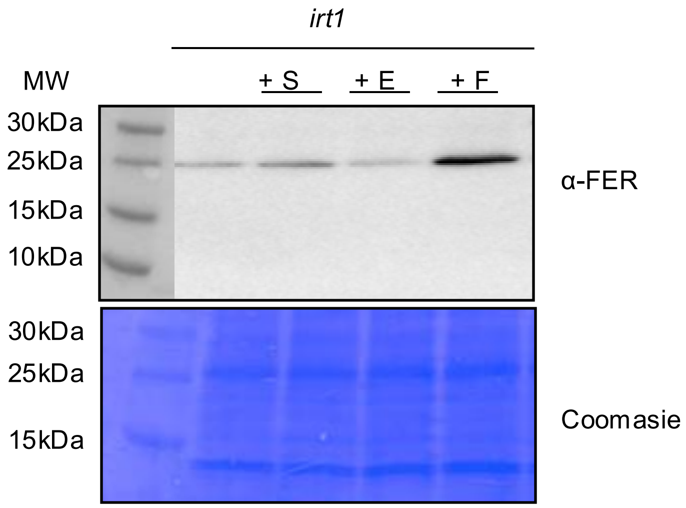

Figure S10. Western Blot of ferritins accumulation in *irt1* mutant supplemented or not with coumarins.
Ferritins accumulation in shoots of 10-day-old *irt1* mutants grown on medium containing 25 µM $FeCl_3$ and supplemented with scopoletin (+S), esculetin (+E) or fraxetin (+F). Each loaded sample was obtained by pooling approximately 10 seedling shoots. MW markers were imaged with white light and ferritins with chemiluminescence on the same membrane. Coomasie is shown as loading control.

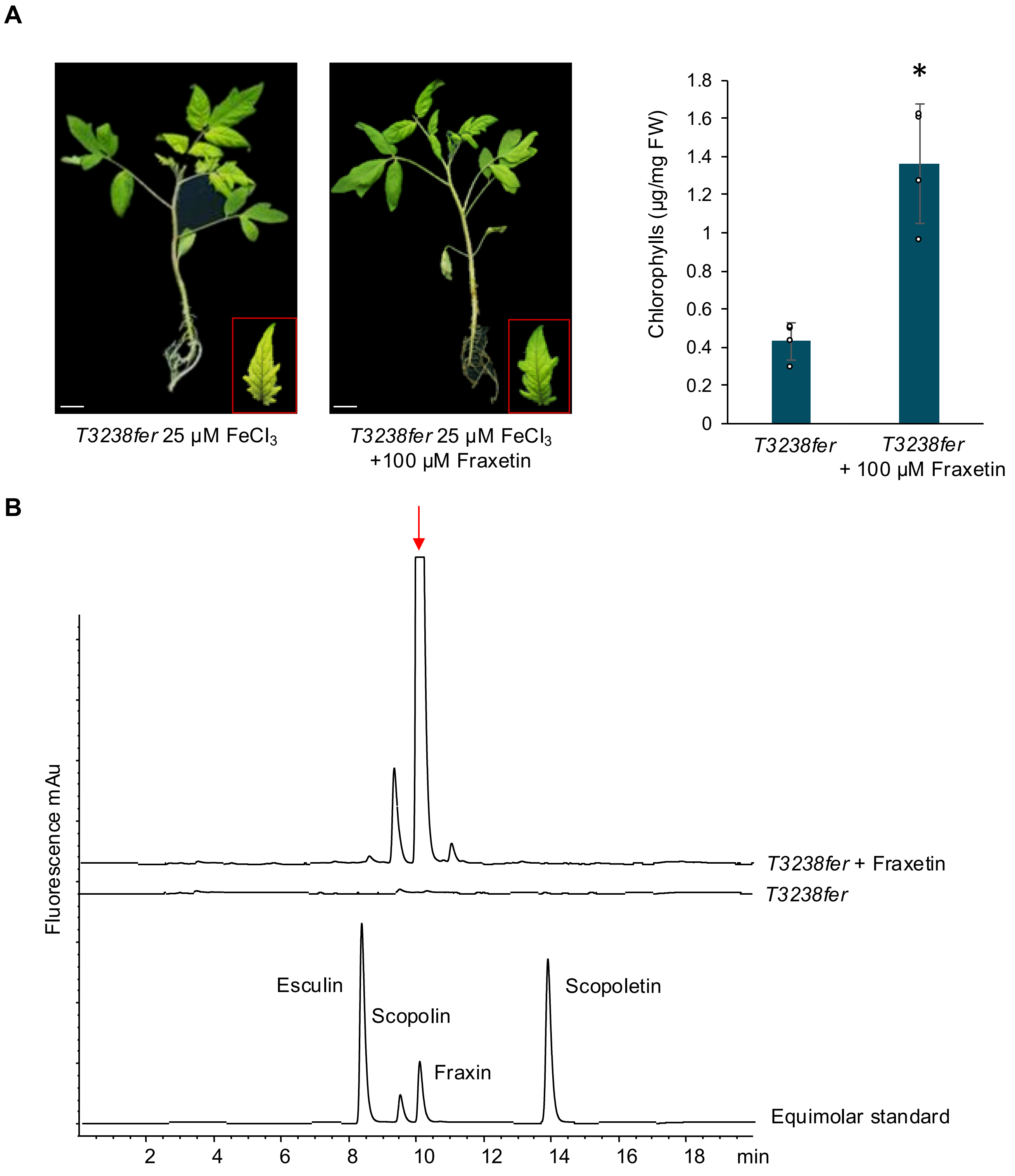

Figure S11. Effect of fraxetin on tomato *T3238fer* mutant.
(A) Complementation of *T3238fer* tomato (*Solanum lycopersicum*) mutant with fraxetin. *T3238fer* mutant was grown for 2 weeks on control Hoagland medium (pH 5.5) and then transferred to Hoagland medium containing 25 µM $FeCl_3$ at pH 7 and supplemented or not with 100 µM Fraxetin for 1 week. Central leaves were used for chlorophyll measurements. *t*-test significant difference: *P < 0.05 (n = 4 biological repeats). Bars represent means ± SD. **(B)** Representative fluorescence chromatograms obtained using λ_{exc} 365 and λ_{em} 460 nm for root extracts of *T3238fer* mutant grown as descibed in (A). The red arrow shows fraxin peak.

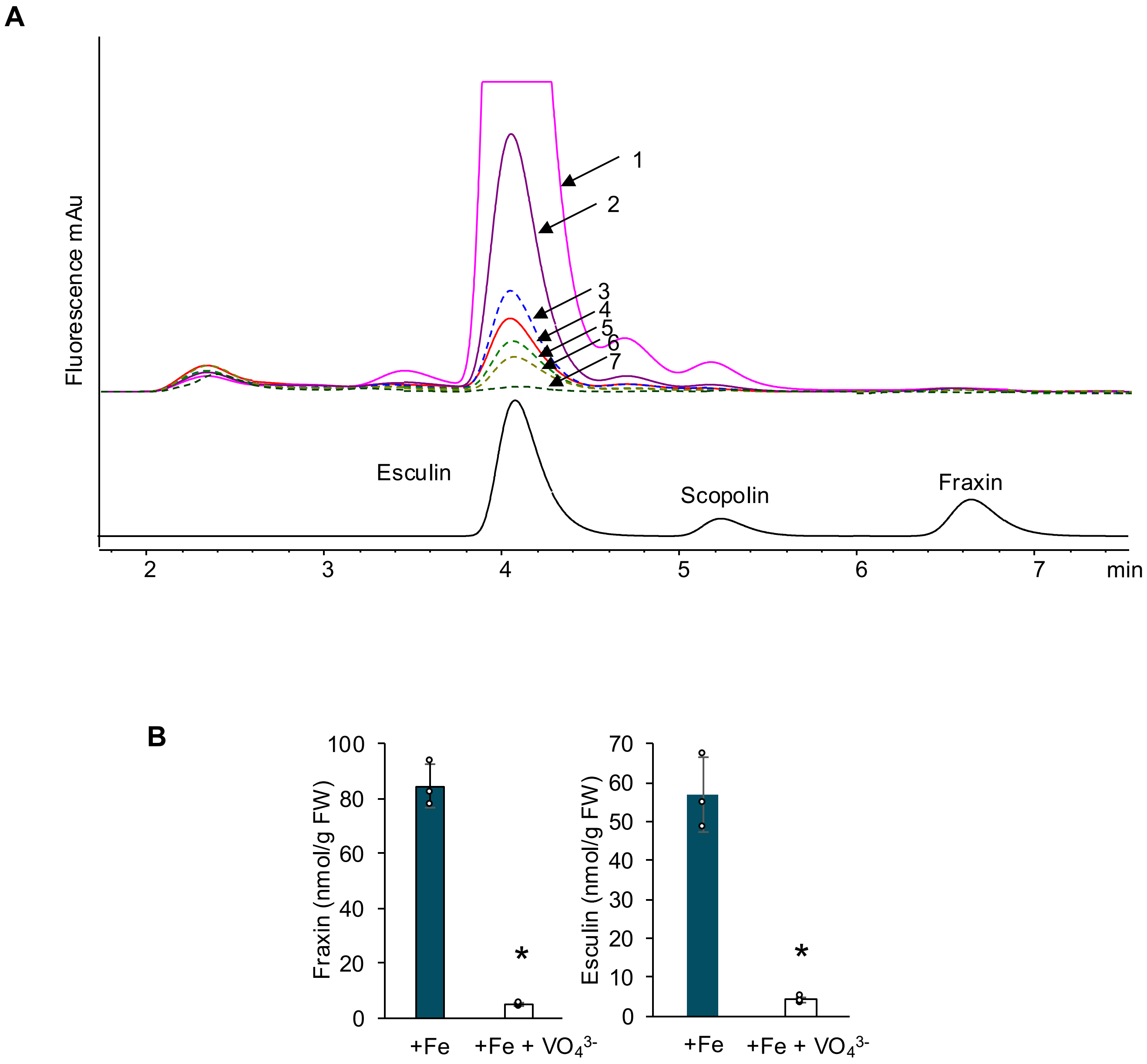

Figure S12 : **Inhibition of coumarin uptake by orthovanadate (VO_4^{3-}) addition.**
(A) Representative fluorescence chromatograms obtained using λ_{exc} 365 and λ_{em} 460 nm for root extracts of Arabidopsis *f6'h1* mutant seedlings grown for 7 days on Hoagland agar plates and then transferred 7 days on Hoagland agar containing poorly available Fe (25 µM $FeCl_3$) and supplemented with 50µM esculetin and different concentrations of orthovanadate (VO_4^{3-}). 1 : + 0 µM VO_4^{3-} , 2 : + 50µM VO_4^{3-}, 3: + 100 µM VO_4^{3-}, 4 : + 200 µM VO_4^{3-}, 5 : + 250 µM VO_4^{3-}, 6: + 500 µM VO_4^{3-}, 7: + 1 mM VO_4^{3-}. **(B)** Uptake of coumarins by roots grown in hydroponic Hoagland solution containing poorly available Fe (+Fe, 25 µM $FeCl_3$), supplemented or not with 50 µM orthovanadate (VO_4^{3-}). Plants were kept for 3 days in the presence of coumarins and orthovanadate. Coumarin glucosides were analysed by HPLC. *t*-test significant difference: *P < 0.05 (n = 3 biological repeats). Bars represent means ± SD.

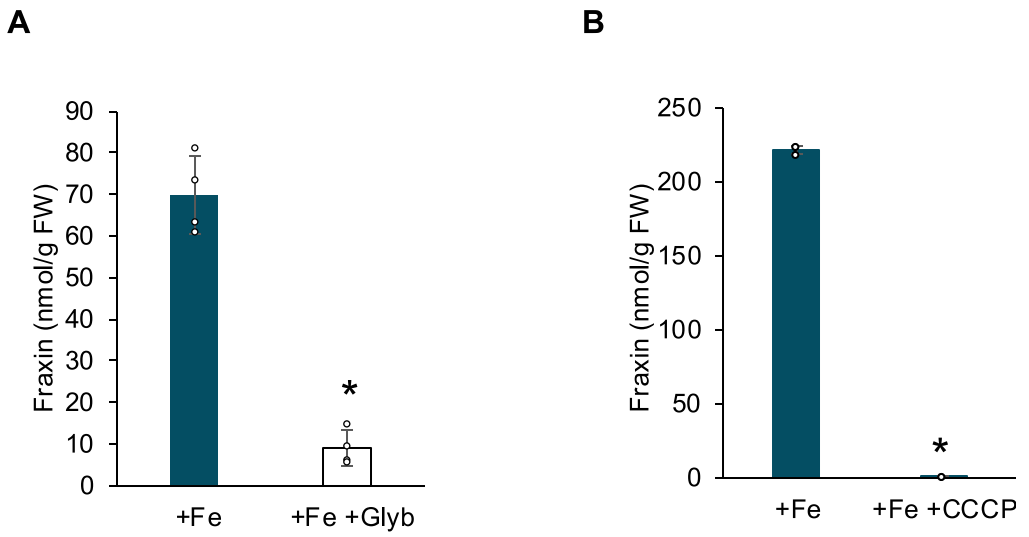

Figure S13. Inhibition of fraxetin uptake by the ATP-dependent transport inhibitor glibenclamide and the proton uncoupler CCCP.
(A) Uptake of fraxetin by roots of Arabidopsis *f6'h1* mutant seedlings grown in hydroponic Hoagland solution containing poorly available Fe (+Fe , 25 µM $FeCl_3$), supplemented or not with 150 µM glibenclamide (Glyb). Plants were kept for 3 days in the presence of fraxetin and glibenclamide and fraxin was analyzed by HPLC. **(B)** Uptake of fraxetin by roots of Arabidopsis *f6'h1* mutant seedlings grown as in (A), supplemented or not with 10 µM CCCP. Plants were kept for 3 days in the presence of fraxetin and CCCP and fraxin was analyzed by HPLC. *t* test significant difference: $^*P < 0.05$ ($n = 4$ biological repeats). Bars represent means ± SD.

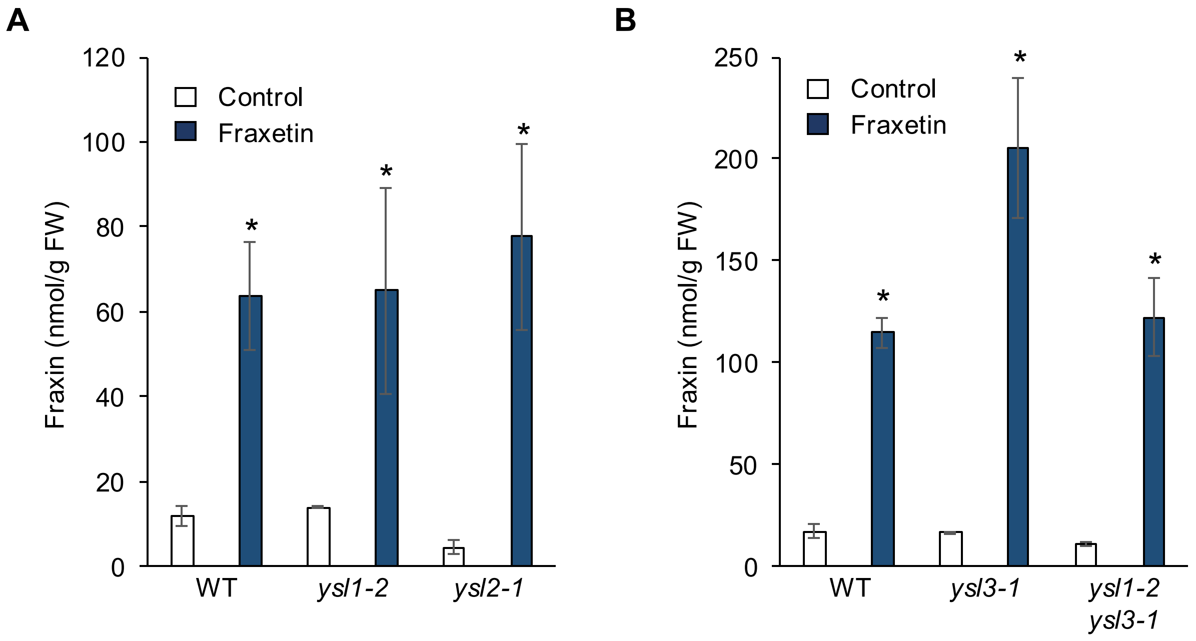

Figure S14. Fraxetin uptake is not inhibited in *ysl1*, *ysl2*, *ysl3* and *ysl1 ysl3* mutants.
(A) Uptake of fraxetin by roots of Arabidopsis wild type (WT) and *ysl1-2* and *ysl2-1* mutant seedlings grown on solid Hoagland medium containing poorly available Fe (+Fe , 25 µM $FeCl_3$) supplemented or not with 100 µM fraxetin. **(B)** Uptake of fraxetin by roots of Arabidopsis WT and *ysl3-1* and *ysl1-2 ysl3-1* mutant seedlings grown on solid Hoagland medium containing poorly available Fe (+Fe , 25 µM $FeCl_3$) supplemented or not with 100 µM fraxetin. **(A-B)** *t*-test significant difference: $^*P < 0.05$ ($n = 3$ biological repeats). Bars represent means ± SD.

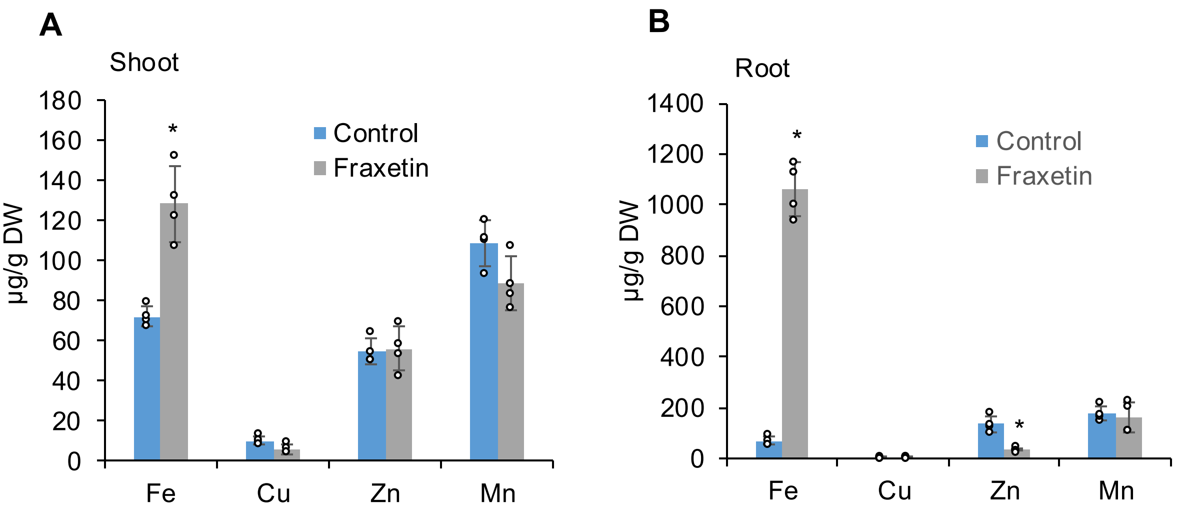

Figure S15. Effect of fraxetin treatment on the accumulation of iron in *Arabidopsis thaliana* WT plants.
(A) Shoots and **(B)** roots Fe and transition metals content of WT plants. Plants were grown for 10 days in control Hoagland agar plates and then transferred for 10 days to new plates containing poorly available Fe Hoagland medium supplemented or not with 100 μM fraxetin. *t* test significant difference: * $P < 0.05$ ($n = 4$ biological repeats). Bars represent means ± SD. Fe: iron, Cu: copper, Zn: zinc and Mn: manganese.

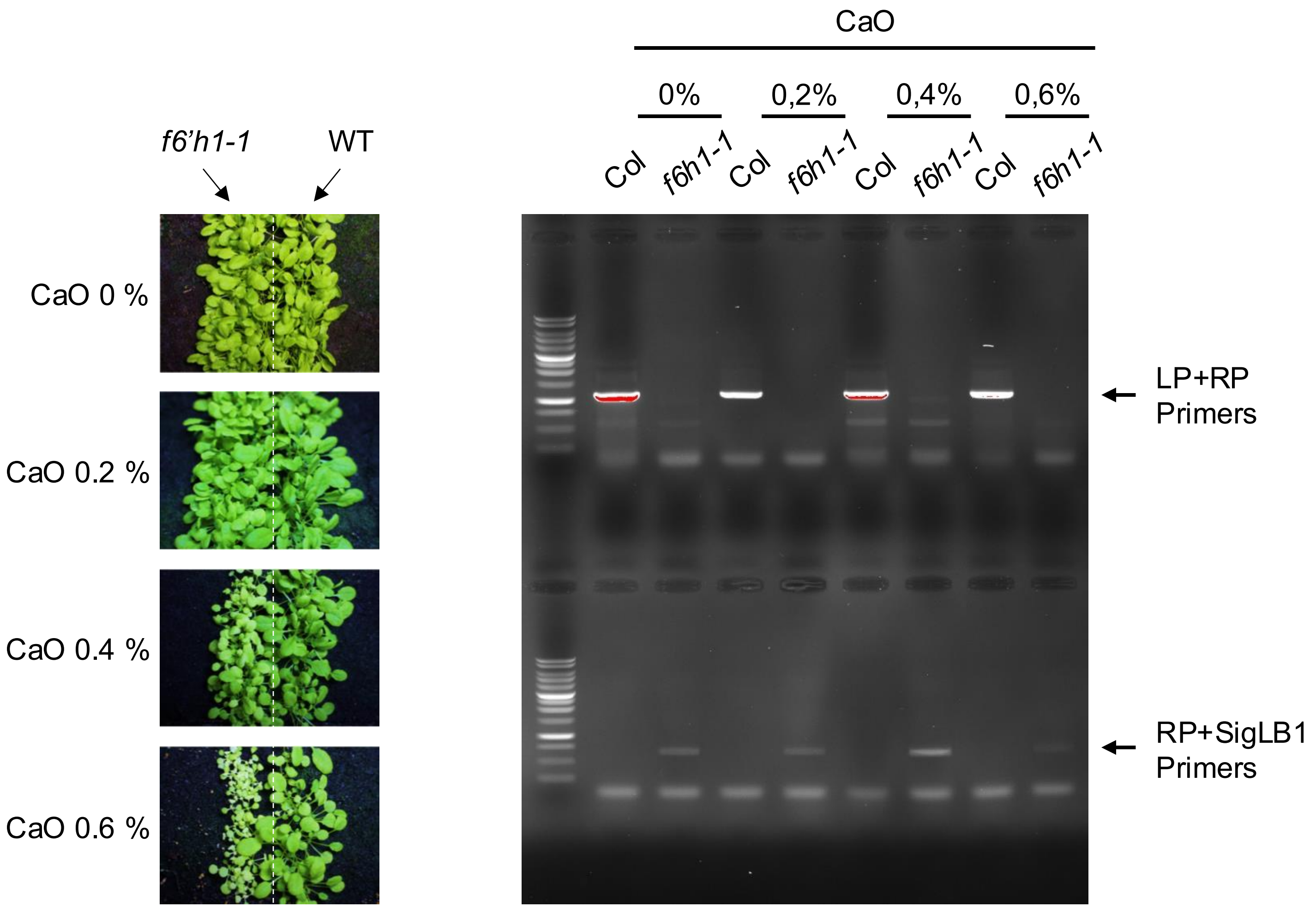

Figure S16. Genotyping of wild type (WT) and *f6'h1* mutant roots prior to coumarins LC-MS/MS analysis. The *F6'H1* wild type allele was amplified with the LP (5'-CCTGTTGCTGTGGAAGAGAAG-3') and RP (5'-GCAGATATCAGGCCAGAACTG-3') primers whereas the T-DNA insertion was assessed with the RP and SigLB1 (5'-CGGAACCACCATCAAACAG-3') primers.

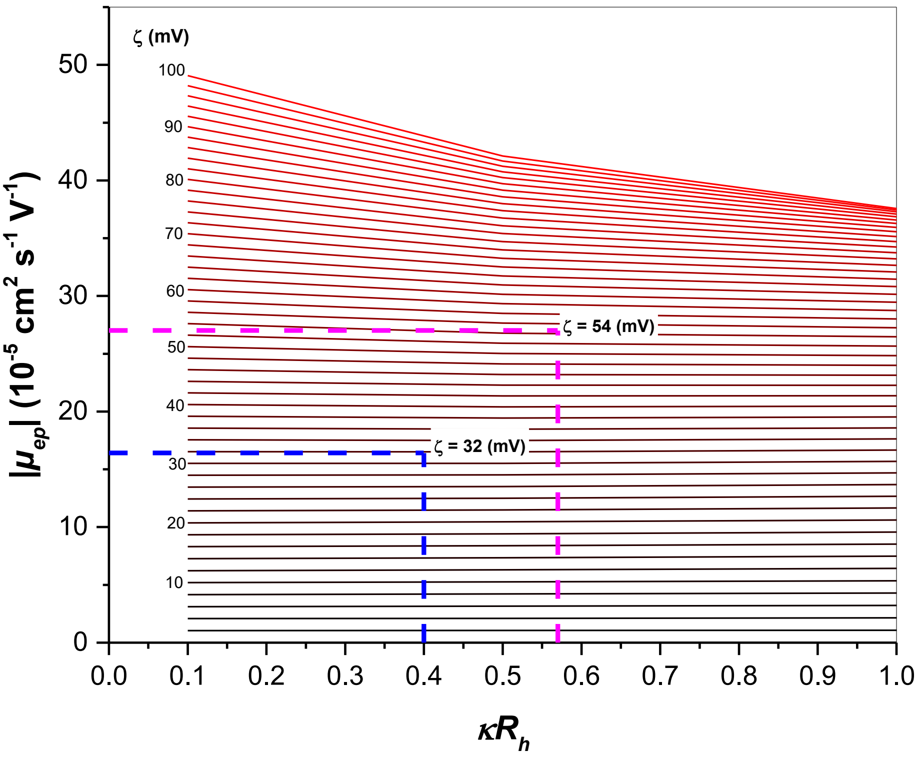

Figure S17. Graphical determination of zeta potential (*ζ*). *ζ* is obtained from the experimental effective mobility (*μep*) of a solute and depending on its hydrodynamic radius and on the ionic strength of the medium according to O'Brien-White-Ohshima model (OWO) (equation (2)), in a symmetrical electrolyte of NH_4HCO_3 ($m+$ = 0.189, $m-$ = 0.288). The dashed lines represent the graphical determination of *ζ* in a 50 mM NH_4HCO_3 buffer pH 7 for: Fraxetin (blue dashed lines, κR_h = 0.39, R_h = 0.49 nm, $|\mu_{ep}|$ = 16.42 × 10^{-5} cm^2 s^{-1} V^{-1}) and Fraxetin-Fe(III) complex (3:1) (magenta dashed lines, κR_h = 0.57, R_h = 0.77 nm, $|\mu_{ep}|$ = 27.02 × 10^{-5} cm^2 s^{-1} V^{-1})

Complex	Elemental composition	Theoretical m/z	Experimental m/z	Theoretical intensity (%)	Experimental intensity (%)	Δ intensity (%)
$[Fe(fraxetin)_2\text{-}4H^+]^-$	$C_{20}H_{12}O_{10}Fe$	465.9832	465.9843	6.4	5.2	1.2
		466.9866	466.9878	1.4	1.1	0.3
		467.9786	467.9804	100	100	0
		468.9816	468.9833	24.4	20.5	3.9
		469.9835	469.9847	5.2	4.1	1.1
$[Fe(fraxetin)_3\text{-}4H^+]^-$	$C_{30}H_{20}O_{15}Fe$	674.0204	674.0211	6.3	6.2	0.1
		675.0237	675.018	2.1	4.2	-2.1
		676.0158	676.0166	100	100	0
		677.0189	677.0198	35.5	35.4	0.1
		678.0211	678.0223	9.5	8.3	1.2

Table S1. Experimental and theoretical mass-to-charge ratios (*m/z*) and isotope abundance of 2:1 and 3:1 fraxetin-Fe complexes detected by Direct infusion ESI-QTOF MS analysis.
Theoretical isotope abundance was calculated using Data Analysis software from Bruker Daltonics

**Stoichiometry**	**Charge**		**R_h (nm)**	**Expected μ_{ep} (x10^{-5} cm^2 V^{-1} s^{-1})**	
**Fe:Fraxetin**	**Fe^{3+}**	**Fe^{2+}**		**Fe^{3+}**	**Fe^{2+}**
1:1	+1	0	0.58[a]	+5.3	0
1:2	-1	-2	0.67[b1] / 0.72[b2]	-12.1[b1] / -10.7[b2]	-23.9[B1] / -21.9[b2]
1:3	-3	-4	0.77[c]	**-27.2**	-32.9

Table S2. Charge, hydrodynamic radius and electrophoretic mobility of Fe:Fraxetin complexes.
The charge of each complex was determined in the case of Fe(III) and Fe(II) and the hydrodynamic radius was determined on the simulated structure. Using the hydrodynamic radius and the charge, the theoretical electrophoretic mobility of each hypothetical complex could be determined using the OWO model and this latter value was compared to the experimental value of 27.02×10^{-5} cm^2 s^{-1} V^{-1} obtained from the analysis of the Fe-Fraxetin mixture. Results show that only the 3:1 was in accordance with the experimental value and thus the other structures could be discarded.

Metal	Molar masses	Isotope abundances (%)
Al	26.9815	100
Mn	54.9380	100
Fe	55.9349	92
	53.9396	6
	56.9354	2
Ni	57.9353	68
	59.9308	26
Cu	62.9296	69
	64.9278	31
Zn	63.9291	48
	65.9260	28
	67.9248	19

Table S3. Natural isotope abundances of the analyzed metals

Supplemental experimental procedures

Methods S1. Detailed protocols for pKa of Fraxetin determination and Effective charge of fraxetin and Fe(III)-fraxetin complex determination

pK_a of Fraxetin.

The determination of the acid constant of fraxetin was realised by capillary electrophoresis by analysing fraxetin at different pH values between 2.5 and 11. The calculated electrophoretic mobility, obtained from the transformation of the temporal electropherogram into an electrophoretic mobility distribution as described in (Chamieh *et al.*, 2015), was then plotted against the pH value and using the equation for a monoprotic acid (Poole *et al.*, 2004) (equation (1) below) a pK_a value could be obtained as a fitting parameter. It is noteworthy that the second acidity of fraxetin was considered weak and not observed under the operating experimental conditions.

$$\mu_{ep} = \frac{\mu_{A^-} \times 10^{-pK_a}}{10^{-pK_a} + 10^{-pH}} \tag{1}$$

Were μ_{A-} is the electrophoretic mobility of fraxetin anion obtained at the plateau of the curve.

Effective charge of fraxetin and Fe(III)-fraxetin complex.

The determination of the effective charge of fraxetin and Fe-fraxetin complexes was achieved by using the method based on the O'Brien-White-Ohshima model (OWO) (Ohshima, 2001; Ibrahim *et al.*, 2013):

$$\mu_{ep} = \frac{2\varepsilon_r\varepsilon_0\zeta}{3\eta}\left[f_1(\kappa R_h) - \left(\frac{ze\zeta}{k_BT}\right)^2 f_3(\kappa R_h) - \frac{m_- + m_+}{2}\left(\frac{ze\zeta}{k_BT}\right)^2 f_4(\kappa R_h)\right] \tag{2}$$

Where ε_r is the relative electric permittivity of the media, ε_0 is the electric permittivity of vacuum (8.85 × 10^{-12} C^2 J^{-1} m^{-1}), ζ is the zeta potential (V), η is the viscosity of the media (Pa s), κ is the Debye–Hückel parameter (m^{-1}) which represents the reciprocal thickness of the ionic cloud, R_h is the hydrodynamic radius (m), k_B is the Boltzmann constant (1.38 × 10^{-23} J K^{-1}), T is the temperature (K), z is the charge number of the electrolyte ions, while f_1, f_3 and f_4 are functions of R_h and are given by:

$$f_1(\kappa R_h) = 1 + \frac{1}{2\left[1 + 2.5/\{\kappa R_h\}\left(1 + 2e^{-\kappa R_h}\right)\right]^3} \tag{3}$$

$$f_3(\kappa R_h) = \frac{\kappa R_h\left(\kappa R_h + 1.3e^{-0.18\kappa R_h} + 2.5\right)}{2\left(\kappa R_h + 1.2e^{-7.4\kappa R_h} + 4.8\right)^3} \tag{4}$$

$$f_4(\kappa R_h) = \frac{9\kappa R_h\left(\kappa R_h + 5.2e^{-3.9\kappa R_h} + 5.6\right)}{8\left(\kappa R_h - 1.55e^{-0.32\kappa R_h} + 6.02\right)^3} \tag{5}$$

While m_+ and m_- are dimensionless ionic drag coefficients, accessible from the limiting equivalent conductivities of the cation Λ_+^0 and the anion Λ_-^0 in the electrolyte and calculated using the following equation:

$$m_\pm = \frac{2\varepsilon_r\varepsilon_0 k_B T N_A}{3\eta z \Lambda_\pm} \tag{6}$$

The formalism suggested by Ohshima (Ohshima, 2001) requires the use of a symmetrical (z:z) electrolyte. If we consider the ammonium bicarbonate (NH_4HCO_3) buffer to be a 1:1 electrolyte with an ionic strength I = C(NH_4^+), the dimensionless ionic drag coefficients of the cations and the anions can be calculated from the corresponding limiting ion conductivities which were found to be of Λ_+^0 = 68.16 S cm^2 mol^{-1} for NH_4^+ at 25°C (calculated from data taken from (Tanaka & Hashitani, 1971) via the Einstein relationship) and Λ_-^0 = 44.71 S cm^2 mol^{-1} for HCO_3^- at 25°C (calculated from data taken from (Zeebe, 2011) via the Einstein relationship). From these limiting ion conductivities, the following ionic drag coefficients were obtained using equation (6): m_+ = 0.189 and m_- = 0.288.

This model can be applied for the effective charge determination of small ions, polyelectrolytes and nanoparticles (Ibrahim *et al.*, 2013) as long as the zeta potential ζ is lower than 100 mV and that κR_h is lower than 10 (Pyell *et al.*, 2009; Ibrahim *et al.*, 2012). Further, κ is a function of the ionic strength of the media, I (mol l^{-1}), and can be calculated using the following equation:

$$\kappa = \left(\sqrt{\frac{\varepsilon_0 \varepsilon_r k_B T}{2 N_A e^2 I 1000}} \right)^{-1} \tag{7}$$

Where N_A is the Avogadro number (6.02 × 10^{23} mol^{-1}), e is the elementary electric charge (-1.6 × 10^{-19} C) and 1000 is the conversion factor from l to m^3.
Next, analytical solutions of the OWO model (equation (2) and Supplemental Figure S16) relating the μ_{ep} to κR_h at different ζ values were plotted. Knowing, experimentally, the electrophoretic mobility μ_{ep} and the hydrodynamic radius of the analysed solute, ζ could be determined graphically and finally used for the calculation of the effective charge, z_{eff}, using the following equation (Makino & Ohshima, 2010):

$$z_{eff} = \frac{8\pi R_h^2 \varepsilon_r \varepsilon_0 \kappa k_B T}{z e^2} \sinh\left(\frac{z e \zeta}{2 k_B T} \right) \times \left[1 + \frac{1}{\kappa R_h} \frac{2}{\cosh^2\left(z e \zeta / 4 k_B T \right)} + \frac{1}{\left(\kappa R_h \right)^2} \frac{8 \ln\left[\cosh\left(z e \zeta / 4 k_B T \right) \right]}{\sinh^2\left(z e \zeta / 2 k_B T \right)} \right]^{1/2}$$

(8)

Supplemental references

Chamieh J, Martin M, Cottet H. 2015. Quantitative analysis in capillary electrophoresis: transformation of raw electropherograms into continuous distributions. *Analytical chemistry* **87**(2): 1050-1057.

Ibrahim A, Meyrueix R, Pouliquen G, Chan YP, Cottet H. 2013. Size and charge characterization of polymeric drug delivery systems by Taylor dispersion analysis and capillary electrophoresis. *Analytical and Bioanalytical Chemistry* **405**(16): 5369-5379.

Ibrahim A, Ohshima H, Allison SA, Cottet H. 2012. Determination of effective charge of small ions, polyelectrolytes and nanoparticles by capillary electrophoresis. *Journal of Chromatography A* **1247**(0): 154-164.

Makino K, Ohshima H. 2010. Electrophoretic Mobility of a Colloidal Particle with Constant Surface Charge Density. *Langmuir* **26**(23): 18016-18019.

Ohshima H. 2001. Approximate Analytic Expression for the Electrophoretic Mobility of a Spherical Colloidal Particle. *Journal of Colloid and Interface Science* **239**(2): 587-590.

Poole SK, Patel S, Dehring K, Workman H, Poole CF. 2004. Determination of acid dissociation constants by capillary electrophoresis. *Journal of Chromatography A* **1037**(1): 445-454.

Pyell U, B□cking W, Huhn C, Herrmann B, Merkoulov A, Mannhardt J, Jungclas H, Nann T. 2009. Calibration-free concentration determination of charged colloidal nanoparticles and determination of effective charges by capillary isotachophoresis. *Analytical and Bioanalytical Chemistry* **395**(6): 1681-1691.

Tanaka K, Hashitani T. 1971. Measurements of self-diffusion coefficients of ammonium ion in aqueous solutions. *Transactions of the Faraday Society* **67**(0): 2314-2317.

Zeebe RE. 2011. On the molecular diffusion coefficients of dissolved CO2,HCO3-, and CO32- and their dependence on isotopic mass. *Geochimica et Cosmochimica Acta* **75**(9): 2483-2498.
