# Supplementary material for: Coumarin-facilitated iron transport: An IRT1-independent strategy for iron acquisition in Arabidopsis thaliana
Source: Plant Commun. 2025 Jun 25;6(9):101431. doi: 10.1016/j.xplc.2025.101431 (PMC12447440; doi:10.1016/j.xplc.2025.101431)
Supplement: Document S2. Article plus supplemental information [file mmc2.pdf]

Plant Communications
Research article

CellPress
Partner Journal

Coumarin-facilitated iron transport: An IRT1-independent strategy for iron acquisition in *Arabidopsis thaliana*

Kevin Robe[1], Max J.J. Stassen[1], Shunsuke Watanabe[1], Javier Espadas[2], Philippe Gonzalez[3], Alice Rossille[1], Meijie Li[1], Sonia Hem[1], Aurélien Roux[2], Véronique Santoni[1], Joseph Chamieh[3], Christian Dubos[1,*] and Esther Izquierdo[1,*]

[1]IPSiM, University of Montpellier, CNRS, INRAE, Institut Agro, Montpellier, France

[2]Department of Biochemistry, University of Geneva, Geneva, Switzerland

[3]IBMM, University of Montpellier, CNRS, ENSCM, Montpellier, France

***Correspondence: Christian Dubos (christian.dubos@inrae.fr), Esther Izquierdo (ester.izquierdo-alegre@inrae.fr)**

https://doi.org/10.1016/j.xplc.2025.101431

ABSTRACT

Iron (Fe) is an essential micronutrient for plant growth and development. Despite its importance, Fe uptake in alkaline soils is challenging for most plants because of its poor bioavailability. Plants have evolved two main strategies to acquire Fe. Grass species release phytosiderophores (PS) into the rhizosphere and take up Fe as Fe(III)-PS complexes via specific transporters (strategy II). Non-grass species, such as *Arabidopsis thaliana*, reduce Fe(III) to Fe(II) at the root surface and transport Fe(II) into the root via the high-affinity transporter IRT1 (strategy I). Additionally, these species secrete catechol coumarins, such as fraxetin, into the rhizosphere to enhance Fe acquisition. Although the role of catechol coumarins in Fe reduction has been clearly demonstrated in acidic soils, their functions under alkaline conditions remain unclear. In this study, we demonstrate that, at circumneutral pH, the catechol coumarin fraxetin forms stable complexes with Fe (III). We also demonstrate that fraxetin significantly improves Fe nutrition, even in mutant plants lacking IRT1 and in the presence of the strong Fe(II) chelator ferrozine, suggesting that plants can bypass the conventional Fe(II)-dependent uptake pathway. These findings support the hypothesis that Fe-coumarin complexes are taken up by plant roots in a manner analogous to Fe(III)-PS complexes in grass species, thereby challenging the current paradigm for plant Fe uptake and suggesting a more unified and flexible model in which strategy I plants can utilize Fe(III)-chelating mechanisms similar to strategy II.

Key words: *Arabidopsis thaliana*, coumarins, fraxin, iron, nutrition

Robe K., Stassen M.J.J., Watanabe S., Espadas J., Gonzalez P., Rossille A., Li M., Hem S., Roux A., Santoni V., Chamieh J., Dubos C., and Izquierdo E. (2025). Coumarin-facilitated iron transport: An IRT1-independent strategy for iron acquisition in *Arabidopsis thaliana*. Plant Comm. **6**, 101431.

INTRODUCTION

Iron (Fe) is a major micronutrient required for plant growth and development, and its availability influences both crop productivity and the quality of derived products (Briat et al., 2015). Although Fe is one of the most abundant elements in soil, plants often experience Fe deficiency (Chen and Barak, 1982), particularly in alkaline soils where Fe solubility is low. In aerated soils, Fe solubility decreases by a factor of 1000 for each pH unit increase between 4 and 9 (Lindsay, 1997). The limited bioavailability of Fe in soil has driven plants to evolve strategies for Fe mobilization and uptake, known as strategy I and strategy II (Gao and Dubos, 2021; Chao and Chao, 2022; Huang et al., 2024).

Grass species (strategy II plants) release phytosiderophores from the mugineic acid (MA) family to solubilize Fe. MAs form stable complexes with both Fe(III) (ferric iron) and Fe(II) (ferrous iron). MA-Fe complexes are then taken up into the root epidermis by transporters of the YELLOW STRIPE 1 (YS1) family (Curie et al., 2001; Weber et al., 2006; Xuan et al., 2006). When expressed in the Fe uptake-deficient yeast mutant *fet3fet4*, the maize (*Zea mays*) ZmYS1 restores yeast growth in MA-containing medium, even in the presence of the Fe(II) chelator bathophenanthroline disulfonic acid (Meda et al., 2007). Intriguingly, despite the physiological importance of phytosiderophore uptake at neutral and alkaline pH, it has been shown that uptake of phytosiderophore-Fe complexes into maize roots is a pH-sensitive process. This sensitivity is attributed to the proton-coupled

 © 2025 The Author(s). Published by Elsevier Inc. on behalf of CAS Center for Excellence in Molecular Plant Sciences, Chinese Academy of Sciences, and Chinese Society for Plant Biology.
This is an open access article under the CC BY license (http://creativecommons.org/licenses/by/4.0/).

symporter function of ZmYS1 (Schaaf et al., 2004). It is notable that other micronutrients, such as copper (Cu), zinc (Zn), nickel (Ni), and cobalt (Co), also form complexes with phytosiderophores and are taken up in this form by the plant (Xuan et al., 2006).

In non-grass species (strategy I), efficient Fe uptake from soil is mediated by a reduction-based mechanism, at least in acidic soils (Kobayashi et al., 2012; Brumbarova et al., 2015). This process involves acidification of the rhizosphere through proton release, reduction of Fe(III) to Fe(II) by a membrane-localized reductase, and subsequent transport of Fe(II) across the plasma membrane by high-affinity Fe transporters. In *Arabidopsis thaliana*, these steps depend on the activities of AHA2 (ATPase2), FRO2 (FERRIC REDUCTION OXIDASE2), and IRT1 (IRON-REGULATED TRANSPORTER1), respectively (Robinson et al., 1999; Vert et al., 2002).

At alkaline pH, FRO2 activity is negligible (Terés et al., 2019); as a result, Fe(III) cannot be effectively reduced by FRO2, leading to low Fe uptake. To compensate for the inactivity of FRO2 under high pH, Fe-mobilizing coumarins (i.e., catechol coumarins such as fraxetin and sideretin in *Arabidopsis thaliana*) are secreted into the rhizosphere by the ABCG transporter PDR9 (PLEIOTROPIC DRUG RESISTANCE 9) (Fourcroy et al., 2014). Coumarins are specialized metabolites derived from the phenylpropanoid pathway (Rodríguez-Celma et al., 2013; Fourcroy et al., 2014; Schmidt et al., 2014; Robe et al., 2021b). In *Arabidopsis thaliana*, F6′H1 (FERULOYL-COA 6-HYDROXYLASE 1), a 2-oxoglutarate-dependent dioxygenase, catalyzes the ortho-hydroxylation of feruloyl-CoA into 6-hydroxyferuloyl-CoA, the common precursor of coumarins, which is then converted into scopoletin by COSY (COUMARIN SYNTHASE) (Rodríguez-Celma et al., 2013; Schmid et al., 2014; Vanholme et al., 2019). Because scopoletin lacks catechol groups, it cannot bind Fe and is therefore not directly involved in Fe acquisition (Rajniak et al., 2018). The two primary catechol coumarins, fraxetin and sideretin, are biosynthesized by S8H (SCOPOLETIN 8-HYDROXYLASE), a 2-oxoglutarate-dependent dioxygenase (Siwinska et al., 2018; Tsai et al., 2018), and CYP82C4, a P450-dependent monooxygenase, respectively. Both genes are upregulated in response to Fe deficiency (Murgia et al., 2011; Rajniak et al., 2018). Whereas alkaline pH favors the biosynthesis of fraxetin, the predominant catechol coumarin secreted under acidic pH is sideretin. The importance of catechol coumarin secretion for Fe nutrition has been demonstrated in multiple studies (Schmid et al., 2014; Rajniak et al., 2018; Tsai et al., 2018; Paffrath et al., 2024). There is evidence that catechol coumarins can reduce Fe(III) to Fe(II) and form stable complexes with Fe(III). However, the relative importance of each process for the plant and the precise role of these metabolites in Fe nutrition remain incompletely understood, particularly in alkaline soils where the reduction of Fe by coumarins is negligible (Paffrath et al., 2024). Three hypotheses can be proposed regarding the role of catechol coumarins in plant Fe nutrition under high pH conditions: (1) catechol coumarins form stable complexes with Fe(III) that are translocated into the roots through the activity of an unidentified mechanism at the root epidermal cells; (2) Fe(III) is reduced by catechol coumarins and directly transported by IRT1; or (3) Fe(III) solubilized by coumarins serves as a substrate for the FRO2 reductase (Rodríguez-Celma and Schmidt, 2013). These hypotheses are not mutually exclusive and may coexist, although the third appears less likely in high pH soils because FRO2 activity is negligible. The uptake of catechol coumarins by strategy I plants grown under Fe-deficient conditions has been previously reported (Robe et al., 2021a); however, the biological implications of this process require elucidation. These findings have led to the consideration of coumarins as putative siderophores, based on their strong similarities to MA.

In this study, we demonstrate that at circumneutral pH, the catechol coumarin fraxetin forms stable complexes with Fe(III). Our data indicate that Fe-coumarin complexes are taken up by plant roots and significantly enhance Fe nutrition, even in mutants lacking the high-affinity Fe(II) transporter IRT1 and in the presence of the strong Fe(II) chelator ferrozine. Remarkably, this newly identified mechanism of coumarin-mediated Fe uptake in *Arabidopsis* closely parallels the uptake of Fe-phytosiderophore complexes in grasses. Collectively, these findings support the notion that boundaries between strategy I and strategy II plants are much narrower than typically presumed.

RESULTS

Coumarin uptake in the presence of iron

The uptake and glycosylation of coumarins by *Arabidopsis* roots under Fe-deficient conditions on agar plates have been previously demonstrated (Robe et al., 2021a). However, this mechanism has not been evaluated in plants grown in soil, where large amounts of insoluble iron hydroxides are expected to accumulate. Accordingly, we investigated the mechanism using *f6′h1* mutant plants grown near wild-type (WT) plants in potting soil supplemented with various concentrations of calcium oxide (CaO; i.e., 0%, 0.2%, 0.4%, and 0.6%). CaO was utilized to increase soil pH and stimulate the production of coumarins such as fraxetin and esculetin (Figure 1A; Rajniak et al., 2018). Because coumarins in roots are predominantly present in their glycosylated form, only coumarin glucosides were quantified in this tissue. Given that no commercial standards are available for sideretin glucosides, mass spectrometry (MS) was employed to unequivocally identify the coumarins produced and taken up (Figure 1B). CaO addition strongly reduced root length; thus, contamination of *f6′h1* roots by the co-cultivated WT roots can be excluded. In WT plants, scopolin and fraxin accumulation increased in a CaO concentration-dependent manner, whereas sideretin glucoside accumulation exhibited the opposite trend. As expected, none of the analyzed coumarins were detected in the roots of *f6′h1 mutants* grown alone across all tested CaO concentrations. However, MS analysis of *f6′h1* roots co-cultivated with WT plants confirmed that fraxetin and the non-catechol coumarin scopoletin were taken up by *Arabidopsis* roots grown in soil. Esculin levels in this experiment were very low and could not be quantified by MS. Sideretin glucosides were detected in *f6′h1* roots co-cultivated with WT plants but not in *f6′h1* roots grown alone, indicating that sideretin can also be taken up by plant roots (Figure 1B). To examine the kinetics of this uptake process, a time-course experiment was performed using *f6′h1* seedlings grown on control medium and then transferred to a medium containing synthetic fraxetin. Samples were collected at 1, 3, 7, 24, 48, and 72 h after transfer; fraxin accumulation was

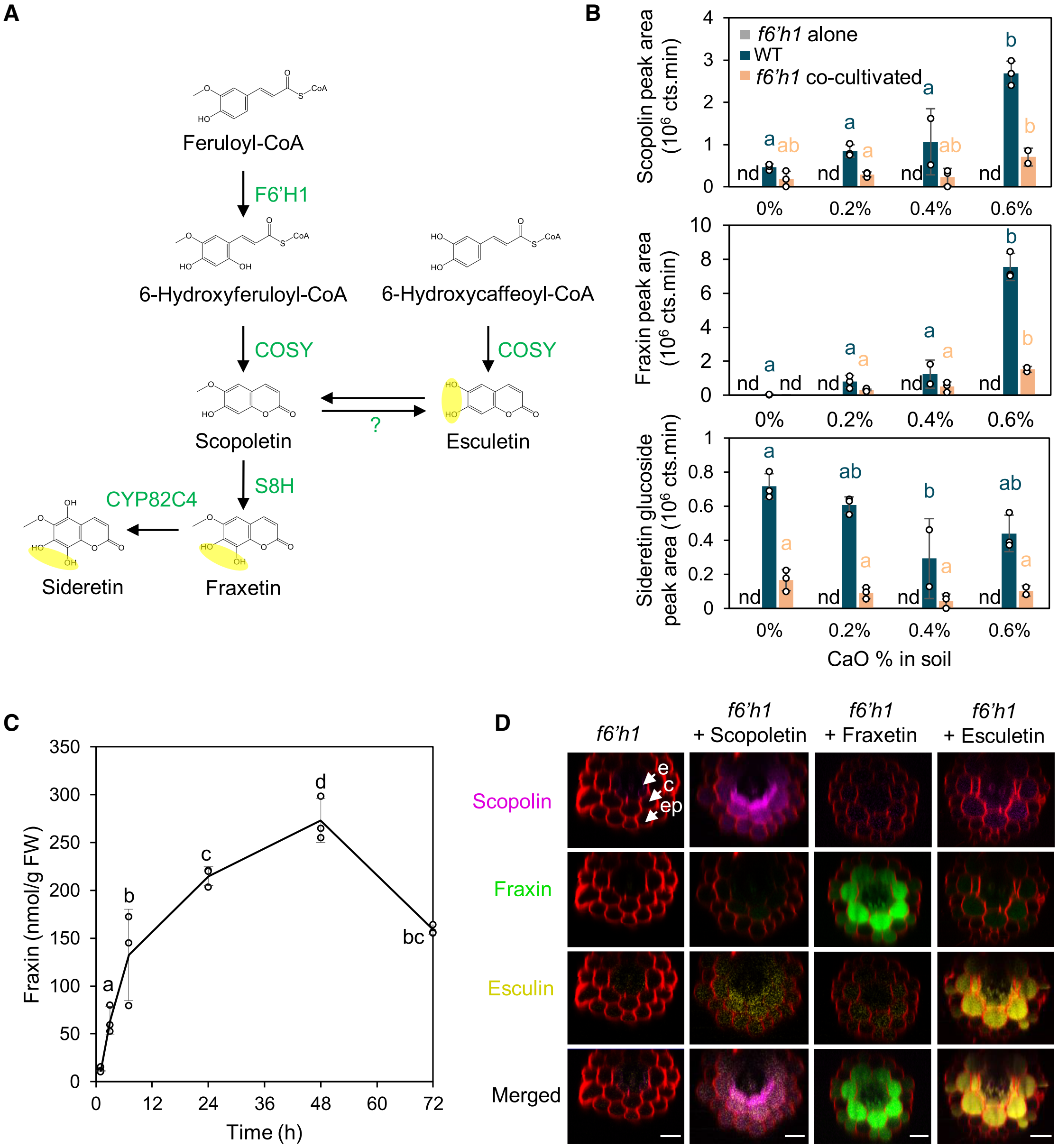

Figure 1. Uptake of coumarins in the presence of iron.

(A) Coumarin biosynthetic pathway. Enzymes are indicated in uppercase green letters. Catechol groups are highlighted in yellow. Note that the conversion of 6-hydroxycaffeoyl-CoA into esculetin has only been demonstrated *in vitro*. Question marks denote putative unknown enzymes.

(B) Scopolin, fraxin, and sideretin glucoside contents in the roots of 3-week-old *Arabidopsis thaliana* wild-type (WT) and *f6′h1* mutants, co-cultivated or not with WT plants, in soil supplemented with varying CaO concentrations (%, w/w). Coumarin quantification was performed using liquid chromatography–tandem mass spectrometry (LC–MS/MS). Coumarins were identified based on their retention times, MS, and MS/MS profiles and quantified by integrating the extracted ion chromatogram (EIC) signals of their aglycone counterparts. EIC values used for quantification were as follows: scopolin, *m/z* 193.04 ± 0.02; sideretin glycoside, *m/z* 225.03 ± 0.02; fraxin, *m/z* 209.05 ± 0.02. nd, not detected. Means within the same genotype sharing the same letter are not significantly different according to one-way analysis of variance (ANOVA) followed by post hoc Tukey test, $p < 0.05$ ($n = 3$ biological replicates of at least 20 seedlings each). Bars represent means ± standard deviation (SD).

(C) Three-day kinetic analysis of fraxetin uptake in *f6′h1* mutants grown in the presence of poorly available Fe (25 μM $FeCl_3$) and exogenous fraxetin. Root samples were collected at the indicated time points, and fraxin was analyzed by HPLC. Means sharing the same letter are not significantly different according to one-way ANOVA followed by post hoc Tukey test, $p < 0.05$ ($n = 3$ biological replicates of at least 50 seedlings each). Error bars represent means ± SD. Lines connect the means.

(D) Localization of coumarins (scopolin, purple; fraxin, green; esculin, dark yellow) in *f6′h1* mutant seedlings grown for 3 days in the presence of synthetic scopoletin, fraxetin, or esculetin. Images were obtained through spectral deconvolution and are representative of three independent experiments, each analyzing roots from five to six seedlings. Scale bar corresponds to 25 μm. e, endodermis; c, cortex; ep, epidermis.

analyzed by high-performance liquid chromatography (HPLC) (Figure 1C). Fraxin was detectable in the roots as early as 1 h after transfer, although at low levels, indicating that both uptake and glycosylation, which converts fraxetin to fraxin, are rapid processes. Fraxin accumulation in the roots increased steadily over the first 48 h and began to decline 72 h after transfer. Spectral imaging was used to investigate the localization of scopolin, fraxin, and esculin after the uptake of their respective aglycone forms by *f6′h1* mutants in the presence of poorly available Fe. For this purpose, *f6′h1 mutants were* grown on a medium supplemented with 25 μM iron chloride ($FeCl_3$) at pH 7 and either catechol or non-catechol coumarins. Spectral imaging confirmed that seedlings were capable of taking up coumarins in the presence of Fe, glycosylating them, and storing the glucosides in vacuoles (Figure 1D). Similar to the accumulation pattern observed under Fe deficiency (Robe et al., 2021a), fraxin and esculin accumulated intensely in the cortex and, to a lesser extent, in the epidermis and endodermis. In contrast, scopolin accumulated predominantly in the endodermis, with weaker signals in the cortex and minimal presence in the epidermis. The absence of fraxetin and esculetin in root hairs (Figure 1D) suggests that coumarins are either not taken up by trichoblasts or, if taken up, are secreted back into the rhizosphere.

Identification and characterization of Fe-catechol coumarin complexes

Previous studies demonstrated the formation of Fe-coumarin complexes, but their characterization remained incomplete (Rajniak et al., 2018; Baune et al., 2020). To investigate Fe-coumarin complexes at circumneutral to alkaline pH, solutions of scopoletin, esculetin, and fraxetin were prepared and mixed with $FeCl_3$ in an ammonium bicarbonate buffer at pH 7. Each mixture was analyzed by direct infusion in positive and negative electrospray ionization (ESI) modes. In positive ESI mode, only free coumarins ionized efficiently, and Fe complexes were barely detectable. In negative ESI mode, Fe(III) complexes with fraxetin and esculetin were identified as singly charged species (Figure 2A and 2B). Fe complexes were readily recognized by the characteristic isotopic profile of Fe (Tsednee et al., 2016); the relative abundances of naturally occurring stable Fe isotopes are approximately ^{54}Fe (5.8%), ^{56}Fe (91.7%), ^{57}Fe (2.2%), and ^{58}Fe (0.3%). When fraxetin/$FeCl_3$ mixtures were infused, the predominant species detected was the 1:2 stoichiometry complex $[Fe^{III}(fraxetin)_2–4H^+]^-$ (m/z = 467.99). Additional complexes, including the 1:3 $[Fe^{III}(fraxetin)_3–4H^+]^-$ (m/z = 676.03) and the 2:4 $[Fe^{III}_2(fraxetin)_4–8H^+]^-$ (m/z = 936.98), were also observed (Figure 2A). Analysis of esculetin/$FeCl_3$ solutions revealed the presence of $[Fe^{III}(esculetin)_2–4H^+]^-$ (m/z = 407.96) (Figure 2B). No Fe complexes with scopoletin were detected. Notably, even in the absence of Fe addition, small quantities of fraxetin-Fe complexes were identified (supplemental Figure 1), most likely due to Fe contamination in the infusion system. To confirm that Fe-fraxetin complexes formed prior to introduction into the MS system, fraxetin was incubated with isotopically labeled $^{57}Fe^{3+}$. In this experiment, the expected 1 Da MS shift was observed for species containing one Fe ($[Fe^{III}(fraxetin)_2–4H^+]^-$ (m/z = 468.98) and $[Fe^{III}(fraxetin)_3–4H^+]^-$ (m/z = 677.02), and a 2 Da MS shift for species containing two Fe atoms ($[Fe^{III}_2(fraxetin)_4–8H^+]^-$ (m/z = 938.97)). Additionally, the isotopic profile of all major species identified as Fe-fraxetin complexes was altered, consistent with the incorporation of ^{57Fe} (supplemental Figure 2). The decrease in free coumarin signal intensity after Fe addition was considerably higher for fraxetin (90%) than for esculetin (25%) (Figure 2A and 2B compared with supplemental Figure 1), indicating that fraxetin is a stronger Fe chelator than esculetin at circumneutral pH. Fraxetin is mostly produced in alkaline soil (Figure 1B), suggesting that Fe-fraxetin complexes exhibit greater stability under these conditions. Intriguingly, upon Fe addition, substantial amounts of oxidized free fraxetin (fraxetin semiquinone, m/z = 206.0221) were detected (Figure 2A); only the reduced form of fraxetin was detected (m/z = 207.0351) in the absence of Fe (supplemental Figure 1). This finding suggests that whereas most solubilized Fe(III) forms stable chelates with fraxetin, redox dissociation of the complex may produce a small fraction of soluble Fe^{2+} and free oxidized fraxetin.

The stabilities of Fe-fraxetin complexes at various pH values were assessed by MS (supplemental Figure 3). No significant change in the signal intensity of the complex (m/z = 467.99) was observed at pH 8 and 7. At pH 6, the signal slightly decreased; at pH 5, 50% of the complex was lost; and at pH 3, almost no complex remained. The loss of complex signal was accompanied by an increase in the free fraxetin signal, confirming that the Fe-fraxetin complex dissociates under acidic conditions (supplemental Figure 3).

Contrary to previous findings (Baune et al., 2020), all Fe-coumarin complexes detected in our study contained Fe(III). Singly charged species of Fe(II) complexes would carry one additional proton compared with Fe(III) complexes and would therefore be 1 Da heavier. The isotopic profiles of the 467.99 and 676.01 m/z peaks closely matched the theoretical profiles (supplemental Table 1), making the presence of +1 Da signals corresponding to Fe(II) complexes highly unlikely. The ESI source represents an artificial environment that induces ionization and does not reflect the actual charge states of species under physiological conditions. Moreover, ionization efficiency varies among species. To further determine whether fraxetin chelates Fe(III) or Fe(II), Fe-fraxetin complexes were analyzed using capillary electrophoresis, a milder technique that preserves molecules in their native state. Figure 2C shows the electrophoretic mobility distributions of fraxetin and a fraxetin/$FeCl_3$ mixture, derived by converting the temporal experimental electropherogram at pH 7 into a mobility distribution (Chamieh et al., 2015). Fraxetin exhibited an average electrophoretic mobility (μ_{ep}) of -16.42×10^{-5} cm^2 s^{-1} V^{-1}, whereas the Fe(III)-fraxetin complex displayed a value of $\mu_{ep} = -27.02 \times 10^{-5}$ cm^2 s^{-1} V^{-1} (Figure 2C). Using these μ_{ep} values along with the hydrodynamic radii of fraxetin (R_h = 0. 49 nm, obtained by Taylor dispersion analysis) and the Fe(III)-fraxetin 1:3 complex (R_h = 0.77 nm, determined by modeling), the effective charges of both species were calculated and found to be −0.8 and −2.9 for fraxetin and the Fe(III)-fraxetin complex, respectively. The effective charge of fraxetin aligns with the pK_a value of 6.61 determined in the present study (supplemental Figure 4**)**. The effective charge of −2.9 for the Fe-fraxetin complex indicates that the main species at pH 7 is the 1:3 stoichiometry Fe(III)-fraxetin complex (Figure 2C and supplemental Figure 5; supplemental Table 2), in which one Fe^{3+} atom is coordinated by three fraxetin molecules, each lacking two protons,

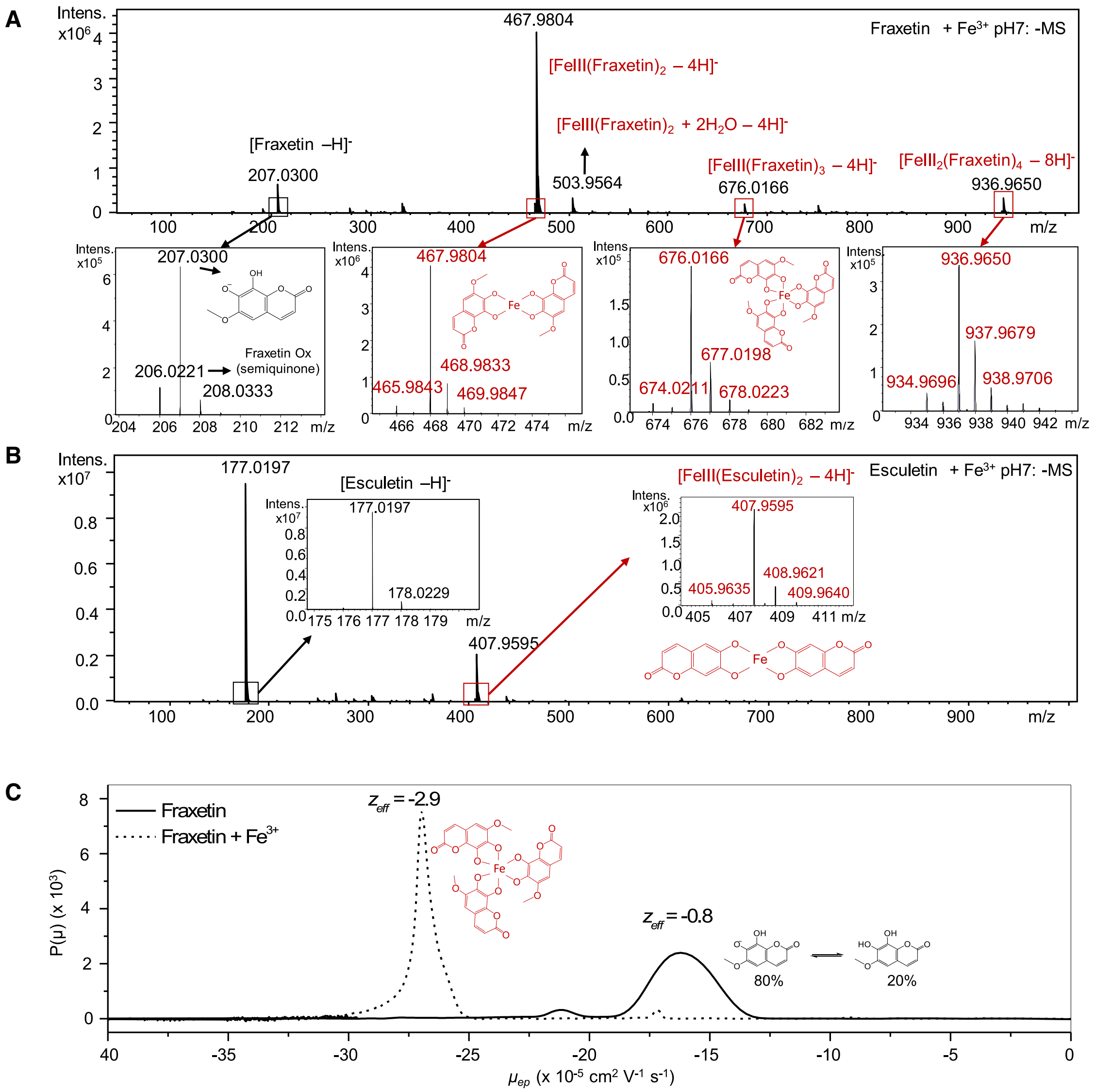

Figure 2. Identification of Fe-fraxetin and Fe-esculetin complexes.

(A) Direct infusion ESI–quadrupole time-of-flight (QTOF) MS analysis of a 2:1 (mol:mol) mixture of synthetic fraxetin and Fe(III) at pH 7.

(B) Direct infusion ESI–QTOF MS analysis of a 2:1 (mol:mol) mixture of synthetic esculetin and Fe(III) at pH 7. **(A–C)** ESI was operated in negative mode. Iron complexes were identified by their characteristic isotopic patterns and are highlighted in red. Boxes show a zoomed-in view of MS signals for the main coumarin species. MS intensity is expressed in counts.

(C) Electrophoretic mobility distributions for fraxetin (solid line) and a 2:1 (mol:mol) mixture of fraxetin and Fe(III) (dashed line) at pH 7 in 50 mM ammonium carbonate buffer. The effective charge (z_{eff}) was calculated using the model described by Ohshima (2001). The structure of the main iron-containing complex is highlighted in red. The relative abundances of protonated and deprotonated forms of fraxetin were calculated from its effective charge and align with its pK_a value.

resulting in an overall charge of −3. Other stoichiometry complexes might also be present at very low concentrations. The higher MS intensity observed for the 467.98 *m/z* peak, corresponding to the 1:2 complex, may reflect superior ionization efficiency of this species and/or *in source* dissociation of the 1:3 complex.

By analogy with phytosiderophores, we explored whether coumarins could form complexes with other transition metals. In particular, we investigated interactions of fraxetin with the divalent cations Ni^{2+}, Zn^{2+}, Mn^{2+}, and Cu^{2+}, as well as the trivalent cation Al^{3+}. The identification of metal-coumarin complexes relied, as with Fe, on metal-specific isotopic signatures observed in the metal complex spectra (supplemental Table 3). Low-intensity signals corresponding to fraxetin complexes with Zn, Al, Ni, Mn, and Cu were detected, with a metal-ligand stoichiometry of 1:2 (supplemental Figure 6). However, in all cases, most fraxetin remained in free ligand form, indicating that the chelation efficiency of these metals by fraxetin is very low relative to Fe. These results demonstrate that fraxetin is a strong and highly specific Fe chelator compared with other transition metals.

Fe-coumarin complexes can rescue *irt1* and *bhlh121* mutants defective in Fe^{2+} uptake

Previous studies showed that the growth defects of *f6′h1 mutants* could be complemented by growing WT plants in close proximity or by adding exogenous coumarins to the culture medium (Tsai et al., 2018; Vanholme et al., 2019). However, the mechanism by which coumarins complement *f6′h1 mutants remains unresolved.* The stability of Fe(III)-fraxetin complexes characterized in this study, as well as in recent work (Kang et al., 2023), supports the hypothesis that secreted catechol coumarins re-enter the root as Fe-coumarin complexes, similar to MAs in grass species. To test this hypothesis, we analyzed *irt1* and *bhlh121* mutants. The *irt1* mutant is unable to transport Fe^{2+} ions from the soil, leading to severe chlorosis (Vert et al., 2002). The recently described *bhlh121* mutant is defective in activating Fe deficiency responses, including the biosynthesis of coumarins (Kim et al., 2019; Gao et al., 2020a). Both mutants exhibit pronounced chlorosis and fail to grow properly unless supplied with high concentrations of Fe (Vert et al., 2002; Kim et al., 2019; Gao et al., 2020a). We first investigated the effects of various fraxetin concentrations on the *bhlh121* mutant phenotype. For this purpose, *bhlh121* mutants were grown for 1 week on Hoagland medium containing 75 μM Fe-EDTA, then transferred to Hoagland medium supplemented with poorly available Fe. As expected, when *bhlh121* mutants were grown in the presence of 25 μM $FeCl_3$ at pH 7, the plants exhibited severe chlorosis with necrotic spots on younger leaves (Figure 3A). The addition of as little as 5 μM fraxetin to the medium partially rescued the phenotype. At this concentration, necrotic spots were no longer detectable, although the leaves remained chlorotic. Increasing the fraxetin concentration progressively alleviated the chlorotic phenotype in a dose-dependent manner. Optimal growth was observed at fraxetin concentrations ranging from 70 to 100 μM (Figure 3A and supplemental Figure 7A). Spectral imaging confirmed that fraxetin was taken up by the roots and glycosylated into fraxin in *bhlh121* mutants (supplemental Figure 7B).

Although the chlorotic phenotype of *bhlh121* was rescued by external addition of fraxetin, we could not exclude the possibility that this effect resulted from the uptake of fraxetin-reduced Fe^{2+} ions by alternative ferrous iron transport systems. To confirm that coumarins directly enter the roots bound to Fe(III), we utilized the strong Fe(II) chelator ferrozine (3-(2-pyridyl)-5,6-diphenyl-1,2,4-triazine-4′,4″-disulfonic acid) to sequester all free Fe^{2+} ions potentially reduced by fraxetin. The ability of fraxetin to rescue the Fe-deficiency phenotypes of *bhlh121 and irt1 mutants* was not diminished in the presence of ferrozine. Plants displayed phenotypes identical to those grown without ferrozine (Figure 3B and 3C). Moreover, ferrozine did not affect fraxetin uptake (supplemental Figure 8). These findings indicate that the complementation of *bhlh121-2* and *irt1* mutant phenotypes by fraxetin does not rely on Fe^{2+} ions and is most likely due to the direct uptake of Fe(III)-fraxetin complexes by plant roots.

The Fe-deficiency phenotypes of *bhlh121*, *irt1*, and other Fe acquisition mutants such as *fro2* and *f6′h1*, grown directly on Hoagland medium containing poorly available Fe, were rescued by the addition of fraxetin and, to a lesser extent, esculetin (Figure 3D–3F). In contrast, scopoletin, which lacks the catechol moiety, was unable to complement any of the mutants (Figure 3D–3F). Notably, *irt1* produces catechol coumarins under both Fe-sufficient and Fe-deficient growth conditions, unlike the *bhlh121* mutant (supplemental Figure 9).

Because ferritin accumulation serves as a proxy for Fe content in plants, western blot analysis was performed on *irt1* seedlings grown on media containing poorly available Fe and supplemented with various coumarins. *irt1* mutants accumulated very low levels of ferritins in leaves. Ferritin accumulation increased when *irt1* mutants were grown with fraxetin but not in the presence of scopoletin or esculetin, confirming that the addition of fraxetin enhanced Fe content in the symplasm (supplemental Figure 10). Considering that esculetin only partially complemented the phenotypes of *irt1* and *bhlh121* mutants and its uptake did not result in ferritin accumulation, it is likely that the primary role of this coumarin is not Fe transport into the roots. Moreover, esculetin secretion is relatively limited (Ziegler et al., 2017; Tsai et al., 2018; Terés et al., 2019); its specific function in plant Fe nutrition, if any, remains to be elucidated.

Uptake of Fe(III)-fraxetin is not restricted to *Arabidopsis*

Several eudicots, such as tomato (*Solanum lycopersicum*), are capable of taking up fraxetin into their roots (Robe et al., 2021a). To determine whether the uptake of Fe-coumarin complexes observed in *Arabidopsis* can also rescue Fe acquisition mutants in other strategy I plants, we grew the T3238*fer* tomato mutant, which is unable to activate the Fe deficiency response, with or without fraxetin supplementation (Ling et al., 2002). After 3 weeks of growth on Hoagland medium containing 75 μM Fe-EDTA, plants were transferred to Hoagland medium containing poorly available Fe, supplemented or not with 100 μM fraxetin. One week after transfer under alkaline conditions, T3238*fer* mutants exhibited pronounced chlorosis. However, T3238*fer* mutants grown in the presence of fraxetin did not display a chlorotic phenotype (supplemental Figure 11A), strongly suggesting that Fe(III)-fraxetin complexes are also taken up in tomato. Furthermore, HPLC analysis of root extracts demonstrated that the T3238*fer* mutant glycosylated the absorbed fraxetin, converting it to fraxin (supplemental Figure 11B). These findings indicate that fraxetin prevents chlorosis in eudicots other than *Arabidopsis thaliana*. The presence of S8H orthologs in numerous eudicot species strongly supports the notion that the uptake of Fe(III)-fraxetin complexes is a widespread Fe acquisition strategy among eudicot plants (Rajniak et al., 2018).

Catechol coumarin uptake is an active mechanism

Several specialized metabolites, such as alkaloids, have been reported to passively diffuse into plant roots from the soil (Selmar et al., 2015). Based on their octanol-water partition coefficient ($logK_{ow}$) values (∼1.5) (Mercer et al., 2013), the coumarins studied here are also predicted to passively diffuse across the plant plasma membrane (Limmer and Burken, 2014; Selmar et al., 2015). However, at pH 7, fraxetin is deprotonated and thus negatively charged (Figure 2C and supplemental Figure 4), which would strongly limit its diffusion capacity. Additionally, the inability of rice and maize to take up fraxetin (Robe et al., 2021a) suggests that catechol coumarin uptake is not passive but requires a specific molecular mechanism present only in strategy I plants. To test this hypothesis, we assessed fraxetin diffusion in liposomes at pH 7 via confocal microscopy. Liposomes were incubated for 1 h in the presence of 1 mM

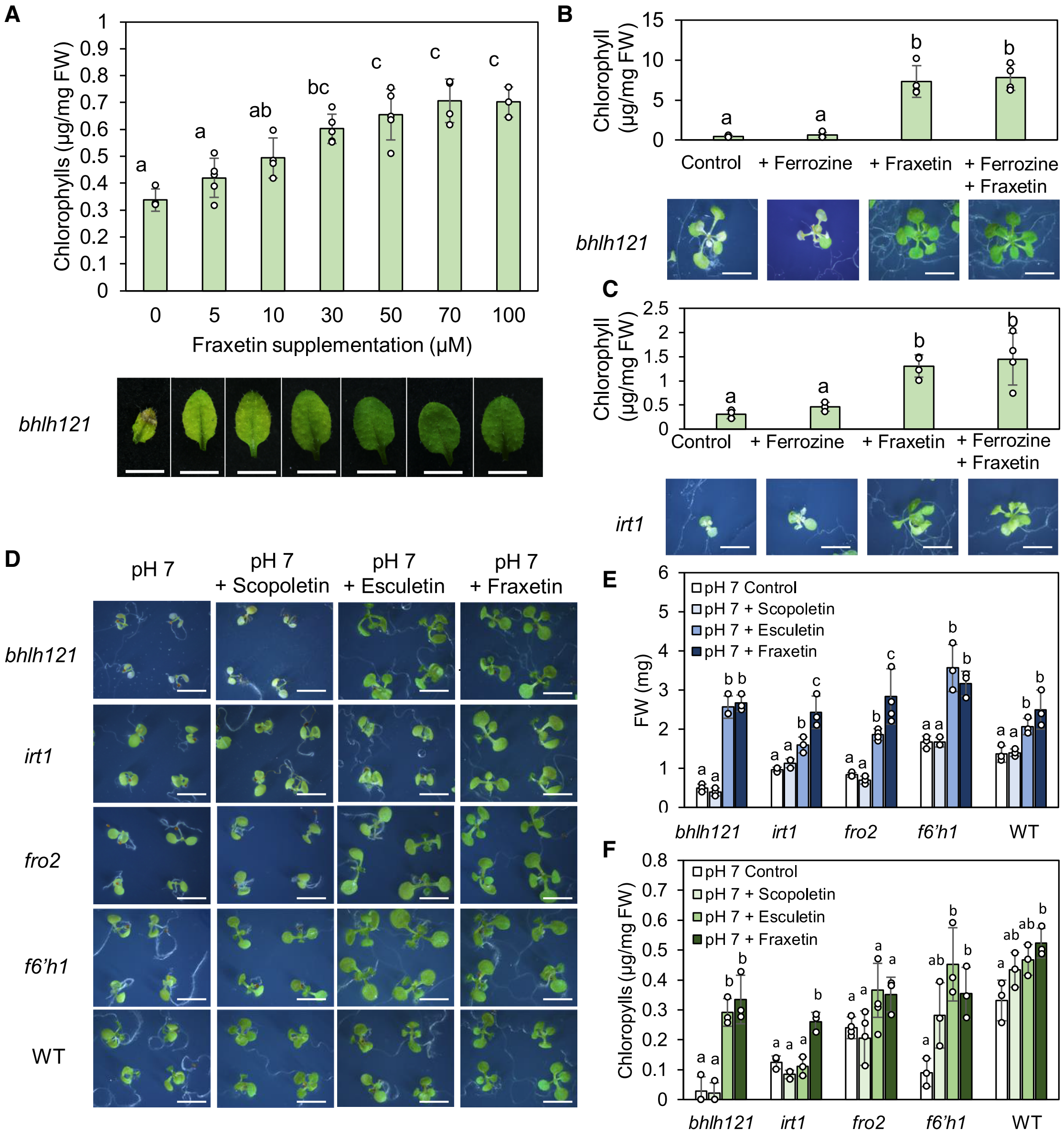

Figure 3. Effects of coumarins on *Arabidopsis thaliana* iron acquisition mutants.

(A) Dose-dependent chlorophyll contents (top) and leaf phenotypes (bottom) of *bhlh121* mutants supplemented with fraxetin. Fraxetin was added at various concentrations to Hoagland medium containing poorly available Fe (25 µM $FeCl_3$).

(B) Chlorophyll contents (top) and phenotypes (bottom) of *bhlh121* mutants grown in the absence of Fe^{2+} ions.

(C) Chlorophyll contents (top) and phenotypes (bottom) of *irt1* mutants grown in the absence of Fe^{2+} ions. **(B and C)** The strong Fe^{2+} chelator ferrozine was added to the medium at 75 µM. **(A–C)** Plants were grown for 1 week on control Hoagland agar plates, then transferred for 1 week to new agar plates containing fraxetin and/or ferrozine.

(D) Phenotypes of several Fe acquisition mutants grown for 10 days on Hoagland medium with poorly available Fe and supplemented with scopoletin, esculetin, or fraxetin. Plants were directly sown on coumarin-supplemented medium.

(E) Fresh weights of shoots from 10-day-old seedlings shown in **(D)**.

(F) Chlorophyll contents of shoots from 10-day-old seedlings shown in **(D)**. **(A–C, E, and F)** Means within each genotype sharing the same letter are not significantly different according to one-way ANOVA followed by post hoc Tukey test, $p < 0.05$ ($n = 3–4$ biological replicates of at least 3 seedlings each). Bars represent means ± SD.

fraxetin prior to observation. We did not detect fraxetin within liposomes, indicating that fraxetin cannot diffuse through lipid membranes (Figure 4A). We next investigated fraxetin lipophilicity by comparison with Fe-chelating molecules previously described as capable of diffusing through lipid membranes (hinokitiol) or not (deferiprone) (Grillo et al., 2017). All three ligands rapidly bound Fe to form colored complexes. Unlike the membrane-permeable hinokitiol-Fe neutral complexes, the negatively charged fraxetin-Fe complexes predominantly partitioned into water rather than polar solvents (Figure 4B). A similar observation was made for Fe chelates with deferiprone, which served as a model for hydrophilic

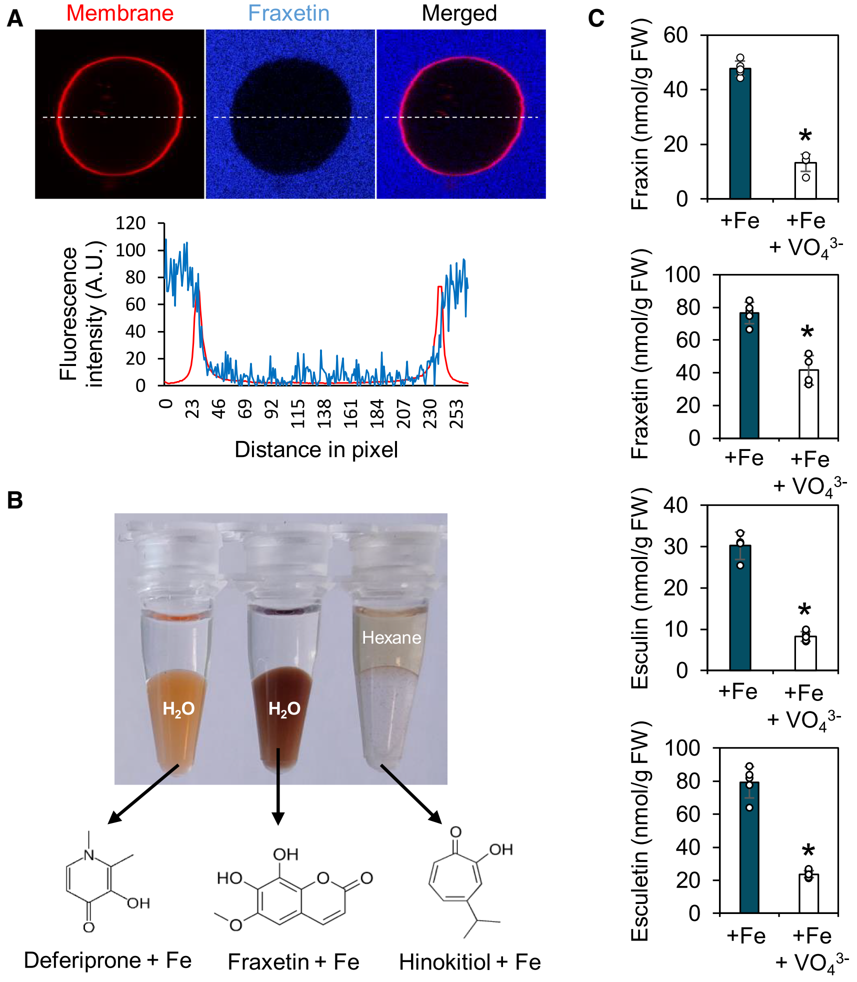

Figure 4. Characterization of the membrane diffusion capacity of fraxetin.
(A) Fraxetin diffusion assay in liposomes. Fraxetin (1 mM) was incubated for 1 h with liposomes. Left panel: red, liposome membrane; blue, fraxetin. Right panel: fluorescence intensity plot of fraxetin (blue) and Atto 647N (lipid labeling, red) across the dashed line.
(B) Phase partitioning of deferiprone, fraxetin, and hinokitiol Fe complexes into hexane (upper phase) and water (lower phase).
(C) Inhibition of coumarin uptake by orthovanadate (VO_4^{3-}). Uptake of coumarins by *f6′h1* roots grown hydroponically in Hoagland solution containing poorly available Fe (+Fe; 25 μM $FeCl_3$), with or without 50 μM VO_4^{3-}. Plants were incubated for 3 h in the presence of coumarins and orthovanadate. Coumarins were quantified by HPLC. Significant differences were determined by *t*-test: $^*p < 0.01$ (n = 5 biological replicates). Bars represent means ± SD.

chelates, showing that both ligands (i.e., fraxetin and deferiprone) form hydrophilic complexes. This finding further supports the conclusion that, due to their biophysical properties, fraxetin-Fe complexes are unlikely to passively diffuse through lipid membranes.

We also investigated the uptake of Fe-mobilizing coumarins using sodium orthovanadate (Na_3VO_4), an inhibitor of plasma membrane ATPases that blocks ABC transporter activities and disrupts the formation of a proton gradient across the plasma membrane (Kang et al., 2011). For this purpose, *f6′h1* mutants were grown under hydroponic conditions and supplemented with two catechol coumarins (fraxetin and esculetin) in the presence or absence of 50 μM orthovanadate for either 3 h or 3 days. The corresponding glucosides (i.e., fraxin and esculin, respectively) were quantified by HPLC. These analyses showed that orthovanadate treatment almost completely inhibited root uptake of fraxetin and esculetin, given that fraxin and esculin contents were reduced by approximately 70% after 3 h of treatment (Figure 4C). Notably, after 3 days, almost no fraxin or esculin was detected in roots by HPLC (supplemental Figure 12), demonstrating that orthovanadate blocks the uptake of fraxetin and esculetin, rather than their glycosylation. There is evidence that orthovanadate treatment does not interfere with vacuolar loading of glycosylated coumarins (Robe et al., 2021a). After 3 h, non-glycosylated coumarins were detectable in hydroponically grown roots (Figure 4C); their levels, similar to those of the glycosylated forms, were strongly reduced by orthovanadate treatment. The active uptake of fraxetin was confirmed using the sulfonylurea glibenclamide, another inhibitor of adenosine triphosphate (ATP)-dependent transport (Forestier et al., 2003), and the proton uncoupler carbonyl cyanide *m*-chlorophenylhydrazone (CCCP) (Schaaf et al., 2004). Both inhibitors, similar to orthovanadate, suppressed fraxetin uptake (supplemental Figure 13A and 13B). These findings demonstrate that catechol coumarin uptake by *Arabidopsis* roots cannot be attributed to passive diffusion and depends on an unidentified ATP-dependent mechanism.

Due to the similarities between MA and coumarin uptake, we speculated that AtYSL (YELLOW STRIPE LIKE) transporters, the closest homologs of ZmYS1, might be involved in fraxetin uptake. Within the AtYSL family, AtYSL1, AtYSL2, and AtYSL3 are the closest homologs to ZmYS1 and thus were selected for further analysis. To test whether these AtYSL transporters mediate fraxetin-Fe uptake, we analyzed *ysl1-2*, *ysl2-1*, *ysl3-1*, and *ysl1-2 ysl3-1* mutants grown on Hoagland agar plates for 1 week before transfer to a medium containing poorly available Fe (25 μM $FeCl_3$), with or without fraxetin supplementation. The amount of fraxetin taken up was then quantified by HPLC. None of the tested *ysl* mutants exhibited reduced fraxetin uptake compared with WT plants (supplemental Figure 14), suggesting that AtYSL1, AtYSL2, and AtYSL3 are not involved in fraxetin-Fe uptake, although functional redundancy among transporters could not be excluded.

Fraxetin uptake increases iron content in plant tissues

To confirm that fraxetin is selectively taken up by roots in the form of Fe(III)-fraxetin complexes, we measured the metal contents of leaves and roots in *bhlh121* and *irt1* mutants supplemented with fraxetin. The analysis revealed that Fe content increased in both

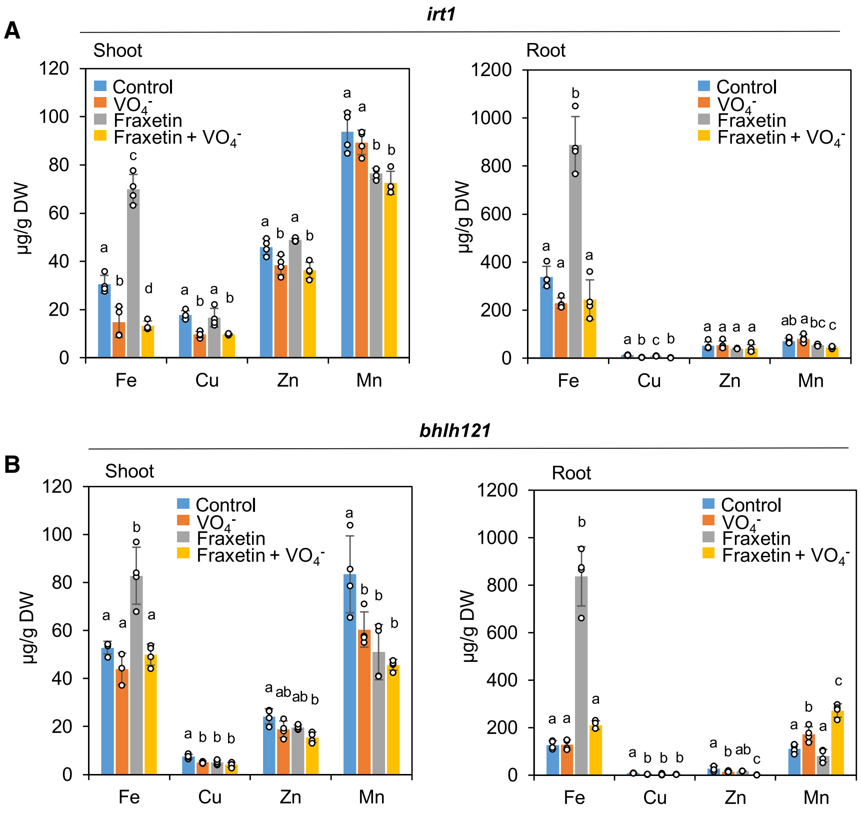

Figure 5. Effect of fraxetin treatment on iron accumulation in *Arabidopsis thaliana* iron acquisition mutants.

(A) Shoot and **(B)** root Fe and transition metal contents in *irt1* and *bhlh121* mutants. Mutants were germinated and grown for 10 days on control agar plates, then transferred for another 10 days to agar plates containing poorly available Fe (25 µM $FeCl_3$) supplemented or not with 100 µM fraxetin and/or 50 µM orthovanadate (VO_4^{3-}). Means within the same metal sharing the same letter are not significantly different according to one-way ANOVA followed by post hoc Tukey test, $p < 0.05$ ($n = 4$ biological replicates of at least 20 seedlings each). Bars represent means ± SD. Fe, iron; Cu, copper; Zn, zinc; Mn, manganese.

roots and shoots of *irt1* and *bhlh121* mutants grown with fraxetin (Figure 5). Notably, this Fe content increase was completely abolished when orthovanadate was also present in the culture medium. Because we previously demonstrated that fraxetin uptake is blocked by orthovanadate (Figure 4B and supplemental Figure 12), the present findings strongly suggest that an unknown ATP-dependent transport system mediates the joint uptake of Fe and fraxetin. To evaluate the contribution of Fe-fraxetin uptake to overall plant Fe acquisition, we compared the metal contents of WT plants grown with and without exogenous fraxetin. The addition of fraxetin resulted in an Fe content increase in WT plants comparable to that observed in *irt1* mutants (supplemental Figure 15A and 15B), indicating that, at circumneutral pH, Fe-fraxetin uptake plays a major role in plant Fe nutrition. Importantly, fraxetin application did not lead to increased levels of other non-ferric metals, consistent with its limited capacity to form complexes with non-ferric metals.

DISCUSSION

Uptake of Fe(III)-coumarin complexes as an alternative iron uptake system

Coumarins have emerged as key metabolites in iron acquisition by non-grass species (Fourcroy et al., 2014; Schmidt et al., 2014; Rajniak et al., 2018; Tsai et al., 2018); however, the mechanisms by which these compounds enhance iron nutrition have remained unclear. Several studies have shown that catechol coumarins participate in Fe reduction and/or Fe chelation (Fourcroy et al., 2016; Sisó-Terraza et al., 2016; Rajniak et al., 2018; Tsai et al., 2018; Paffrath et al., 2024). We recently demonstrated that the catechol coumarin fraxetin is taken up by several non-grass species, suggesting that this process represents a widespread phenomenon within the plant kingdom (Robe et al., 2021a). Whether this uptake is accompanied by Fe uptake has remained an open question (Rodríguez-Celma and Schmidt, 2013; Tsai et al., 2018). By complementing mutants defective in Fe (II) uptake (i.e., *irt1* and *bhlh121*) with synthetic fraxetin, we provide strong genetic evidence that *Arabidopsis* can directly take up Fe(III)-fraxetin complexes from the growth medium. Intriguingly, other Fe(III) chelates (i.e., Fe(III)-EDDHA [Fe-ethylenediamine-*N*,*N*′-bis(2-hydroxyphenylacetic acid)], and Fe (III)-HSAL [Fe-hydrothermal sulfuric acid lignin]) failed to complement such mutants (i.e., *irt1*), supporting the hypothesis that a specific uptake transport mechanism for Fe(III)-fraxetin complexes exists (Liu et al., 2023; Paffrath et al., 2024). The *fro2* mutant was also complemented by exogenous fraxetin at pH 7, demonstrating that the role of coumarins at high pH is not limited to providing chelated Fe(III) for FRO2 activity, as recently suggested (Paffrath et al., 2024). Complementation assays conducted in the presence of ferrozine confirmed this conclusion. Additionally, we showed that at pH 7, fraxetin and Fe interact *in vitro* to form highly specific and stable Fe(III)-fraxetin complexes with a 1:3 metal-to-ligand stoichiometry, similar to those observed for grass MAs. Given that both fraxetin uptake and fraxetin-mediated Fe uptake are abolished in the presence of orthovanadate, our results strongly suggest that fraxetin contributes to plant iron nutrition by forming stable Fe(III) chelates that are directly taken up by the root system (Figure 6) through a specific ATP-dependent mechanism.

Several pathways for re-entry into the root after secretion

Although the secretion of catechol coumarins via the PDR9 transporter has been clearly established, the mechanism underlying their re-uptake remains far more elusive (Fourcroy et al., 2014). In this study, we provide evidence for a long-suspected parallel iron acquisition system in non-grass species. This IRT1/FRO2-independent system was previously proposed by Vansuyt et al. (2007), who demonstrated that iron from pyoverdine, a bacterial siderophore, is not incorporated through the classical IRT1 transporter. Similar to fraxetin-Fe complexes,

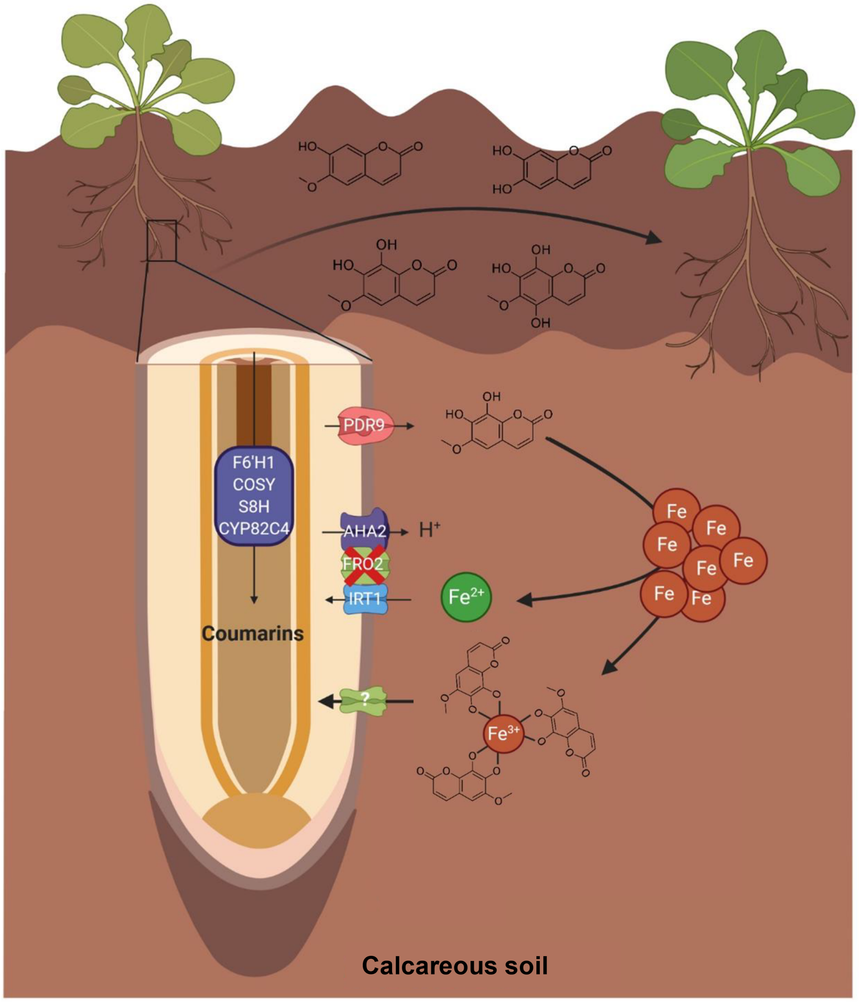

Figure 6. Proposed model for Fe-coumarin uptake by *Arabidopsis thaliana* roots.

In calcareous or alkaline soils, the FRO2/IRT1 high-affinity Fe^{2+} transport system is nearly inactive because FRO2 activity is strongly inhibited at high pH. In response to these growth conditions, *Arabidopsis* roots produce and secrete several coumarins, including scopoletin, esculetin, fraxetin, and sideretin. Once secreted via PDR9, catechol coumarins solubilize Fe hydroxides by forming stable complexes with Fe(III) and by reducing Fe(III) to Fe(II). Reduced Fe is then taken up by IRT1, whereas Fe(III)–coumarin complexes are transported by an unidentified transporter localized in the plasma membrane of root epidermal cells. Fraxetin is the primary catechol coumarin involved in Fe(III) uptake, predominantly acting in a 3:1 fraxetin-to-Fe ratio.

several Fe-ligand complexes have been suggested to enter plants through alternative pathways. Tipton and Buell (1970) proposed that benzoxazinoids, secreted into the rhizosphere by maize roots and capable of strongly binding iron, can function as phytosiderophores. Similarly, Honda and Borthakur (2020) provided evidence that mimosine acts as a siderophore in the leguminous nitrogen-fixing tree *Leucaena leucocephala*. Notably, the peanut AhYSL1 transporter mediates the uptake of maize-derived siderophores, indicating that at least some strategy I plants can utilize strategy II siderophores (Xiong et al., 2013).

Two potential mechanisms could explain the entry of Fe-coumarins into roots: passive diffusion across the plasma membrane or active transport through a specific transporter. The negative charge of Fe-fraxetin complexes and the phase partitioning results presented here strongly argue against passive diffusion. Moreover, orthovanadate treatment substantially impaired the uptake of catechol coumarins, and fraxetin uptake was associated with increased Fe accumulation, supporting the likelihood of an active transport mechanism. ABC transporters are plausible candidates because they transport a wide variety of ions and metabolites, and they are inhibited by orthovanadate. Alternatively, the inhibition of catechol coumarin uptake by orthovanadate could result from the inhibition of plasma membrane-localized ATPases, such as AHA2, whose activity maintains the proton gradient required for transport. The inhibitory effect of CCCP on coumarin uptake further supports this hypothesis, although an indirect effect due to disrupted ATP synthesis cannot be ruled out. Overall, given the broad effects of the inhibitors used—particularly in *in vivo* systems—and their inability to exclusively block ATP-dependent processes, further studies are necessary to determine the precise molecular components responsible for Fe-coumarin complex import. Nevertheless, the data presented here strongly suggest the involvement of a proton-coupled transport mechanism for Fe-coumarin complexes, analogous to the Fe-MA uptake mediated by ZmYS1. Considering the substrates of YSL transporters (nicotianamine bound to various metals) and the inability of ZmYS1's closest homologs to transport fraxetin, the AtYSL family seems unlikely to be involved in Fe-fraxetin uptake. AtNRAMP1 activity in *irt1* and *bhlh121* mutants could potentially explain the positive effect of fraxetin supplementation in these mutants, although the *in planta* role of AtNRAMP1 in Fe uptake has not been clearly demonstrated. Moreover, fraxetin supplementation continues to complement *irt1* and *bhlh121* mutants even in the presence of ferrozine. However, because NRAMP1 transports Fe (II), this hypothesis would require Fe(III) to be reduced by fraxetin, an activity that is negligible at neutral pH (Paffrath et al., 2024). Extensive studies will be required to identify and characterize the mechanism responsible for Fe-coumarin complex uptake. It is also probable that the uptake mechanism for non-catechol coumarins, such as scopoletin, differs from that of fraxetin and occurs independently of Fe availability.

The advantage of a parallel iron uptake system in strategy I plants

From an ecological perspective, the presence of a chelation-based Fe(III) acquisition system in strategy I plants likely confers a strong advantage under soil conditions—such as high pH (Römheld and Marschner, 1983; Terés et al., 2019)—that inhibit Fe reduction by FRO2. This reduction step is critical for Fe

acquisition via the high-affinity Fe(II) transporter IRT1 and, to a lesser extent, NRAMP1. In contrast, grasses utilize a proton-coupled, high-affinity MA-Fe transport system that enables them to thrive in high-pH soils. Proton-coupled MA-Fe transport remains functional as long as the local pH gradient generated by proton extrusion is maintained and the overall electrochemical potential difference remains negative. The data presented here support the existence of a fraxetin-Fe complex uptake system in non-grass species that is highly similar to MA-Fe uptake in grasses. This system may explain how non-grass species, including *Arabidopsis*, are able to grow in carbonate-rich soils with limited bioavailable Fe.

We have found that fraxetin, unlike MAs, has limited capacity to chelate non-ferric metals. This limitation arises because fraxetin contains a catechol moiety that strongly coordinates Fe(III) due to its high charge density, hard acid character, and preference for octahedral coordination, resulting in highly stable tris-catecholate complexes. In contrast, divalent metals such as Cu (II), Ni(II), and Mn(II), which are softer and less charged, interact more weakly with catechol moieties. Such selectivity enables fraxetin to mobilize Fe(III) efficiently without facilitating the uptake of non-target metals, which likely provides plants with an advantage in maintaining metal homeostasis under Fe-limiting conditions.

It has been shown that fraxetin secretion in 22 different *Arabidopsis* accessions is strongly correlated with plant Fe content, and accessions that secrete large amounts of fraxetin exhibit superior growth parameters (Tsai et al., 2018). Additionally, local *Arabidopsis* populations adapted to alkaline soils (pH 7.5) display high fraxin content in roots and a strong capacity for fraxetin secretion (Terés et al., 2019). In contrast, root ferric reductase activities are comparable between these populations and pH-sensitive ones (Terés et al., 2019).

The identification of a second Fe acquisition system represents a major step toward fully understanding Fe uptake in non-grass plant species and may have important implications for improving agricultural practices to prevent Fe deficiency. Selecting plants that secrete higher levels of fraxetin could significantly reduce Fe-induced chlorosis in the field without the need for synthetic Fe chelates, thereby improving cultivation and yields in soils with low Fe availability. Previous studies have demonstrated that grass species can enhance Fe nutrition in non-grass plants within intercropping systems (Brown et al., 1991; Hopkins et al., 1992; Kamal et al., 2000; Gunes et al., 2007; Xiong et al., 2013). It is therefore plausible to hypothesize that the transporter involved in Fe-coumarin complex uptake also mediates the uptake of phytosiderophores. However, previous evidence suggests that the reverse scenario is unlikely (Robe et al., 2021a).

MATERIALS AND METHODS

Plant materials

Arabidopsis thaliana ecotype Columbia (Col-0) was used as the WT. *Arabidopsis thaliana* mutant lines used in this study were as follows: *f6′h1-1*(Kai et al., 2008), *irt1-2* (Varotto et al., 2002), *fro2* (*frd1-1*) (Robinson et al., 1999), *bhlh121-2* (Gao et al., 2020a, 2020b), *ysl1-2* (Le Jean et al., 2005), *ysl2-1* (DiDonato et al., 2004), *ysl3-1*, and *ysl1-2 ysl3-1* (Waters et al., 2006). The Fe-inefficient tomato (*Solanum lycopersicum*) mutant used in this study was T3238*fer* (Brown et al., 1971).

Growth conditions

For *in vitro* culture, seeds were surface sterilized in a solution of bleach, water, and ethanol (12.5:37.5:50, v/v/v) for 5 min with vigorous agitation. Seeds were rinsed three times with 96% ethanol and allowed to dry. They were then germinated on control Hoagland medium (50 μM Fe-EDTA [pH 5.5]) solidified with 0.7% agar and grown under long-day conditions (16 h light/8 h dark).

For all experiments, control Hoagland medium contained 50 μM Fe-EDTA. Poorly available Fe Hoagland medium contained 25 μM $FeCl_3$, supplemented with 0.5 g L^{-1} 4-(2-hydroxyethyl)-1-piperazineethanesulfonic acid (HEPES) buffer, and adjusted to pH 7 with KOH. For agar plates, pH was adjusted after autoclaving using pH indicator paper (±0.25 pH units).

For coumarin uptake assays, *f6′h1* mutant plants were grown hydroponically for 4 weeks in control Hoagland solution. Coumarins were then added for either 3 h or 3 days at a concentration of 50 μM in fresh Hoagland solution containing poorly available Fe (25 μM $FeCl_3$ [pH 7]). For orthovanadate and glibenclamide treatments, seedlings were transferred for 3 h or 3 days into Hoagland solution containing poorly available Fe, coumarins, and either 100 μM sodium orthovanadate or 150 μM glibenclamide (both from Sigma-Aldrich).

For coumarin uptake kinetics and CCCP treatments, 1-week-old seedlings were transferred to Hoagland agar plates containing poorly available Fe and 50 μM fraxetin.

For *in vitro* mutant complementation, plants were germinated directly on Hoagland plates containing poorly available Fe (25 μM $FeCl_3$ [pH 7]) supplemented with synthetic coumarins (scopoletin, fraxetin, or esculetin; all from Sigma-Aldrich). Scopoletin was supplied at 25 μM, esculetin at 50 μM, and fraxetin at 100 μM. Higher concentrations of scopoletin or esculetin (100 μM) were avoided due to observed detrimental effects on seedlings.

Tomato seeds were germinated for 1 week on moist tissue paper under long-day conditions. When cotyledons were fully developed, seedlings were transferred to Hoagland solution containing 25 μM $FeCl_3$ (pH 7.5), with or without 100 μM fraxetin supplementation. Plants were subsequently grown under short-day conditions (8 h light/16 h dark).

Coumarin imaging

Seedlings were grown for 1 week on control Hoagland agar plates (50 μM Fe-EDTA [pH 5.5]) prior to transfer for 3 days to low Fe availability medium (25 μM $FeCl_3$ [pH 7]) supplemented with 25 μM scopoletin, fraxetin, or esculetin. Root cell walls were stained with 10 μg mL^{-1} propidium iodide for 10 min. Coumarins were imaged using an LSM 780 multiphoton (two-photon) microscope (Zeiss, Oberkochen, Germany) equipped with a Chameleon Ultra II laser (Coherent, Santa Clara, CA, USA) and a W Plan-Apochromat 20×/1.0 objective (Koyyappurath et al., 2015; Talamond et al., 2015). Spectral imaging was performed using laser

excitation at 720 nm and the 32-channel GaAsP spectral detector, as previously described (Robe et al., 2021a). The Linear Unmixing function in Zeiss Zen microscope software (version 2.10) was used to separate the signals of the autofluorescent compounds scopolin, fraxin, esculin, and propidium iodide.

Chlorophyll content

Chlorophyll was extracted from 2–5 mg of leaf tissue (fresh weight) in 1 mL of 100% acetone in the dark with agitation. Absorbance (A) was measured at 661.8 and 644.8 nm. Total chlorophyll content was calculated using the following equation: Chl a + Chl b = $7.05 \times A_{661.6} + 18.09 \times A_{644.8}$. Content was expressed as micrograms per gram of fresh weight. Three seedlings were pooled for each biological replicate.

HPLC analysis of root and leaf extracts

Coumarin extraction from roots, sample preparation for HPLC, and coumarin quantification were performed as previously described (Robe et al., 2021a). HPLC–UV and HPLC–fluorescence analyses were conducted using a 1220 Infinity II LC system (Agilent Technologies) coupled to a 1260 Infinity II fluorescence detector (Agilent Technologies). Separation was carried out on an analytical HPLC column (Kinetex 2.6 μm XB-C18 100 Å, 150 × 4.6 mm; Phenomenex) with a gradient mobile phase consisting of 0.1% (v/v) formic acid in water (A) and 0.1% (v/v) formic acid in acetonitrile (B) at a flow rate of 0.5 mL min^{-1}. The gradient program was as follows: 8% B for 1 min, increasing linearly to 33% B over 11.5 min, then to 50% B in 0.5 min. This proportion was maintained for 3 min before returning linearly to the initial conditions in 0.5 min. The column was equilibrated for 5 min at the starting conditions. Absorbance was monitored at λ = 338 nm; fluorescence was monitored with excitation at λ_{exc} = 365 nm and emission at λ_{em} = 460 nm.

LC–MS/MS analysis of soil grown roots

WT and *f6′h1* plants were co-cultivated for 3 weeks in soil supplemented with 0–0.6% (w/w) CaO. Plants were sufficiently spaced to prevent root intermixing between genotypes. Soils containing *Arabidopsis* plants were rinsed roughly with tap water, and roots from both genotypes were carefully washed with distilled water to remove residual soil particles. The absence of cross-contamination between the roots of the two genotypes was verified by polymerase chain reaction genotyping (supplemental Figure 16). Coumarins were then extracted from roots as previously described for HPLC analysis (Robe et al., 2021a).

LC–MS/MS analyses were performed using an Ultimate 3000 nano HPLC system (Thermo Fisher Scientific) coupled to a quadrupole time-of-flight mass spectrometer (Maxis Impact; Bruker Daltonics), as previously described (Robe et al., 2021a). The LC conditions were identical to those used for HPLC analysis, except that the flow rate was reduced to 200 μL min^{-1}. Glycosylated coumarins often lose their sugar moiety in the ESI source. Therefore, after their identities had been verified according to retention time, MS, and MS/MS profiles, fraxin, esculin, scopolin, and sideretin glycosides were quantified by integrating the extracted ion chromatogram (EIC) peaks of their aglycone counterparts. Given that no commercial standards are available for sideretin glycosides, calibration curves could not be established; thus, relative quantification was utilized for all coumarins in this experiment. Results are expressed as relative intensity peak areas (counts min^{-1}).

Direct infusion MS

To investigate the *in vitro* interactions of Fe and other transition metals with coumarins, 50 μM methanolic solutions of each ligand (fraxetin, scopoletin, and esculetin) and 50 μM aqueous solutions of each metal salt ($FeCl_3$, $MnCl_2$, $ZnCl_2$, $NiCl_2$, $AlCl_3$, and $CuCl_2$) were freshly prepared. $^{57}Fe^{3+}$ was obtained from $^{57}Fe_2O_3$ (Eurisotop, Saint-Aubin, France) via overnight solubilization in 37% HCl, followed by neutralization with 1 M ammonium bicarbonate. Metal/ligand mixtures at a 1:2 ratio were prepared and diluted 1:1000 in a solution of 50% acetonitrile and 50% 25 mM ammonium bicarbonate, adjusted to pH 8, 7, 6, 5, or 3 using formic acid. The resulting solutions were injected into the mass spectrometer through a syringe pump at a flow rate of 200 μL h^{-1}. Source settings were the same as those used for LC–MS analysis, except for the mass range (50–1000 *m/z*) and with ESI operated in negative mode.

Capillary electrophoresis and Taylor dispersion analysis

Capillary electrophoresis experiments were performed using an Agilent 7100 capillary electrophoresis instrument equipped with a built-in diode array UV detector (Waldbronn). Detection was carried out at 214 nm. Fused silica capillaries (50 μm inner diameter/375 μm outer diameter) were obtained from Polymicro Technologies. The capillaries were 33.5 cm in length (25 cm to the detection window). New capillaries were conditioned with sequential washes at 1 bar: 1 M NaOH for 30 min, followed by water for 15 min. Before each sample injection, the capillary was flushed with buffer for 2 min. Samples were hydrodynamically injected at the inlet side of the capillary by applying 50 mbar pressure for 3 s. Separations were conducted under a −15 kV voltage. Each injection was repeated three times, with a 30-s buffer flush between repetitions. Samples were prepared at a concentration of 1 μM fraxetin in 50 mM ammonium bicarbonate (NH_4HCO_3) buffer adjusted to various pH levels (5–8) with formic acid, or in 50 mM formic acid solution at pH 2.5.

The hydrodynamic radius of fraxetin was determined via Taylor dispersion analysis (Chamieh and Cottet, 2014). Briefly, using the same capillary, instrument, and buffers as in the capillary electrophoresis experiments, samples were hydrodynamically injected by applying a 50 mbar pressure for 5 s and mobilized with the buffer under 100 mbar pressure. Elution profiles were analyzed as previously described (Chamieh and Cottet, 2014) to calculate the diffusion coefficient and determine the hydrodynamic radius (R_h). For Fe(III)–fraxetin complexes, R_h was estimated from the diffusion coefficient using HydroPro software (Ortega et al., 2011) and structures drawn in Marvin Sketch (ChemAxon).

The effective charges of fraxetin and Fe-fraxetin complexes were determined using a method based on the O'Brien-White-Ohshima model (Ohshima, 2001; Ibrahim et al., 2013). Detailed protocols and equations for determining the effective charge of fraxetin and Fe(III)–fraxetin complexes, as well as the pKa of fraxetin, are provided in supplemental Methods 1 (supplemental Figure 17).

Fraxetin diffusion in liposomes

Giant unilamellar vesicles were prepared as previously described (Velasco-Olmo et al., 2019). Dioleoyl-phosphatidylcholine, dioleoyl-phosphatidylserine, and dioleoyl-phosphoethanolamine labeled with Atto 647N (Atto 647N DOPE) lipid stocks dissolved in chloroform were mixed at 59.9:40:0.1 molar percentages, respectively, in a glass vial and dried under vacuum for 2–3 h. The dried lipid film was then hydrated in a buffer containing 25 mM HEPES (pH 7.4) to form multilamellar vesicles (MLVs) at a final concentration of 1 g L^{-1}. Twenty microliters of the MLV solution were mixed with 2 μL of silica beads (40 μm in diameter) (Microspheres-Nanospheres) and deposited on parafilm. The MLV–silica bead mixture was dried under vacuum for 2 h to form supported bilayers on the silica beads. The supported silica beads were collected using a glass capillary and placed in a hydration chamber containing 1 M trehalose at 60°C for 15 min as a membrane pre-hydration step. The pre-hydrated model membranes were transferred to a 1.5-mL tube containing the working buffer (25 mM HEPES [pH 7.4]) and further hydrated for 20 min. The mixture was gently stirred for 20–30 s to detach the hydrated model membranes from the supporting silica beads. The supernatant, containing detached giant unilamellar vesicles, was transferred to a new tube containing fraxetin dissolved in the working buffer at a final concentration of 1 mM. After 20 min of incubation, samples were imaged using a scanning confocal microscope (inverted Nikon Eclipse Ti A1R microscope with a ×100, 1.3 NA oil immersion objective [Nikon]).

Lipophilicity determination

To investigate hexane–water partitioning of iron chelates, 40 μM solutions of each ligand (fraxetin, deferiprone, and hinokitiol; Grillo et al., 2017) and 13 μM $FeCl_3$ were prepared in 25 mM carbonate buffer (pH 8). The solutions were mixed with an equal volume of hexane, shaken for 5 min, and centrifuged for 5 min to separate the phases.

Metals measurements

Approximately 2.5 mg of dry root tissue or 7.5 mg of dry shoot tissue per sample were mixed with 750 μL of nitric acid (65% [v/v]) and 250 μL of hydrogen peroxide (30% [v/v]). After incubation overnight at room temperature, samples were mineralized at 85°C for 24 h. Following mineralization, 4 mL of Milli-Q water were added to each sample. Metal contents were then measured using microwave plasma atomic emission spectroscopy (MP-AES; Agilent Technologies). Approximately 100 seedlings were pooled for each biological replicate.

Western blot analysis

Western blotting was performed as previously described (Robe et al., 2021a). Immunodetection of ferritins was carried out using a rabbit polyclonal antiserum raised against purified FER1 (Dellagi et al., 2005). The antibody dilutions used were 1:10 000 for rabbit anti-ferritin primary antibody and 1:10 000 for horseradish peroxidase-conjugated anti-rabbit secondary antibody (Promega).

ACCESSION NUMBERS

Sequence data from this article can be found in the GenBank/EMBL databases under the following accession numbers: *bHLH121* (AT3G19860), *FRO2* (AT1G01580), *F6′H1* (AT3G13610), *IRT1* (AT4G19690), *YSL1* (AT4G24120), *YSL2* (AT5G24380), and *YSL3* (AT5G53550).

FUNDING

We thank Sandrine Chay (IPSiM, SAME platform, Montpellier, France) for technical support for plant Fe determination, Dr. Marie Lopez (IBMM, Montpellier, France) for help with coumarin chemistry, and Dr. Brigitte Touraine (IPSiM) for help with HPLC analysis of coumarins. We also thank Dr. Catherine Curie (IPSiM) for kindly providing seeds for *ysl* mutants. We thank the imaging facility MRI, a member of the national infrastructure France-BioImaging, supported by the French National Research Agency (ANR-10-INBS-04, "Investments for the Future"). Support for this work was provided to C.D. by the Agence Nationale de la Recherche (ANR-17-CE20-0008 and ANR-22-CE20-0006) and the National Research Institute for Agriculture, Food and the Environment (INRAE, BAP Department). K.R. was supported by a fellowship from the Agence Nationale de la Recherche (ANR-17-CE20-0008) and the BAP Department of INRAE. A.R. was supported by a fellowship from the Agence Nationale de la Recherche (ANR-22-CE20-0006) and the BAP department of INRAE. M. L. was supported by the China Scholarship Council, and S.W. by a Marie Skłodowska-Curie Individual Fellowship in Horizon (2020) from the European Council (MSCA-IF-2020). A.R. acknowledges funding from the Swiss National Fund for Research (nos. CRSII5_189996 and 310030_200793) and the European Research Council Synergy Grant (no. 951324-R2-TENSION). J.E. acknowledges a long-term EMBO fellowship (EMBO ALTF 989-2022).

ACKNOWLEDGMENTS

No conflict of interest declared.

AUTHOR CONTRIBUTIONS

K.R., J.C., C.D., and E.I. conceived and designed the experiments. K.R., M.J.J.S., S.W., P.G., A.R., M.L., J.C., and E.I. performed the experiments. K.R., M.J.J.S., S.W., J.E., P.G., A.R., M.L., S.H., J.C., C.D., and E.I. analyzed the data. S.H. and V.S. provided access to MS facilities. K.R., J.C., C.D., and E.I. wrote the paper with the help of all the authors.

SUPPLEMENTAL INFORMATION

Supplemental information can be found online at https://doi.org/10.1016/j.xplc.2025.101431.

Received: November 20, 2024
Revised: April 28, 2025
Accepted: June 23, 2025
Published: June 25, 2025

REFERENCES

Baune, M., Kang, K., Schenkeveld, W.D.C., Kraemer, S.M., Hayen, H., and Weber, G. (2020). Importance of oxidation products in coumarin-mediated Fe(hydr)oxide mineral dissolution. Biometals **33**:305–321.

Briat, J.-F., Dubos, C., and Gaymard, F. (2015). Iron nutrition, biomass production, and plant product quality. Trends Plant Sci. **20**:33–40.

Brown, J.C., Chaney, R.L., and Ambler, J.E. (1971). A New Tomato Mutant Inefficient in the Transport of Iron. Physiol. Plant. **25**:48–53.

Brown, J.C., Von Jolley, D., and Lytle, C.M. (1991). Comparative evaluation of iron solubilizing substances (phytosiderophores) released by oats and corn: Iron-efficient and iron-inefficient plants. Plant Soil **130**:157–163.

Brumbarova, T., Bauer, P., and Ivanov, R. (2015). Molecular mechanisms governing Arabidopsis iron uptake. Trends Plant Sci. **20**:124–133.

Chamieh, J., and Cottet, H. (2014). Chapter 9 - Size-based characterisation of nanomaterials by Taylor dispersion analysis. In

Colloid and Interface Science in Pharmaceutical Research and Development, H. Ohshima and K. Makino, eds. (Amsterdam: Elsevier), pp. 173–192.

Chamieh, J., Martin, M., and Cottet, H. (2015). Quantitative analysis in capillary electrophoresis: transformation of raw electropherograms into continuous distributions. Anal. Chem. **87**:1050–1057.

Chao, Z.F., and Chao, D.Y. (2022). Similarities and differences in iron homeostasis strategies between graminaceous and nongraminaceous plants. New Phytol. **236**:1655–1660.

Chen, Y., and Barak, P. (1982). Iron Nutrition of Plants in Calcareous Soils. In Advances in Agronomy, *35*, N.C. Brady, ed. (Academic Press), pp. 217–240.

Curie, C., Panaviene, Z., Loulergue, C., Dellaporta, S.L., Briat, J.-F., and Walker, E.L. (2001). Maize yellow stripe1 encodes a membrane protein directly involved in Fe(III) uptake. Nature **409**:346–349.

Dellagi, A., Rigault, M., Segond, D., Roux, C., Kraepiel, Y., Cellier, F., Briat, J.-F., Gaymard, F., and Expert, D. (2005). Siderophore-mediated upregulation of Arabidopsis ferritin expression in response to Erwinia chrysanthemi infection. Plant J. **43**:262–272.

DiDonato, R.J., Jr., Roberts, L.A., Sanderson, T., Eisley, R.B., and Walker, E.L. (2004). Arabidopsis Yellow Stripe-Like2 (YSL2): a metal-regulated gene encoding a plasma membrane transporter of nicotianamine-metal complexes. Plant J. **39**:403–414.

Forestier, C., Frangne, N., Eggmann, T., and Klein, M. (2003). Differential sensitivity of plant and yeast MRP (ABCC)-mediated organic anion transport processes towards sulfonylureas. FEBS Lett. **554**:23–29.

Fourcroy, P., Sisó-Terraza, P., Sudre, D., Savirón, M., Reyt, G., Gaymard, F., Abadía, A., Abadia, J., Álvarez-Fernández, A., and Briat, J.-F. (2014). Involvement of the ABCG37 transporter in secretion of scopoletin and derivatives by Arabidopsis roots in response to iron deficiency. New Phytol. **201**:155–167.

Fourcroy, P., Tissot, N., Gaymard, F., Briat, J.-F., and Dubos, C. (2016). Facilitated Fe Nutrition by Phenolic Compounds Excreted by the Arabidopsis ABCG37/PDR9 Transporter Requires the IRT1/FRO2 High-Affinity Root Fe2+ Transport System. Mol. Plant **9**:485–488.

Gao, F., and Dubos, C. (2021). Transcriptional integration of plant responses to iron availability. J. Exp. Bot. **72**:2056–2070.

Gao, F., Robe, K., Bettembourg, M., Navarro, N., Rofidal, V., Santoni, V., Gaymard, F., Vignols, F., Roschzttardtz, H., Izquierdo, E., et al. (2020a). The Transcription Factor bHLH121 Interacts with bHLH105 (ILR3) and Its Closest Homologs to Regulate Iron Homeostasis in Arabidopsis. Plant Cell **32**:508–524.

Gao, F., Robe, K., and Dubos, C. (2020b). Further insights into the role of bHLH121 in the regulation of iron homeostasis in Arabidopsis thaliana. Plant Signal. Behav. **15**:1795582.

Grillo, A.S., SantaMaria, A.M., Kafina, M.D., Cioffi, A.G., Huston, N.C., Han, M., Seo, Y.A., Yien, Y.Y., Nardone, C., Menon, A.V., et al. (2017). Restored iron transport by a small molecule promotes absorption and hemoglobinization in animals. Science **356**:608–616.

Gunes, A., Bagci, E.G., and Inal, A. (2007). Interspecific Facilitative Root Interactions and Rhizosphere Effects on Phosphorus and Iron Nutrition Between Mixed Grown Chickpea and Barley. J. Plant Nutr. **30**:1455–1469.

Honda, M.D.H., and Borthakur, D. (2020). Mimosine facilitates metallic cation uptake by plants through formation of mimosine-cation complexes. Plant Mol. Biol. **102**:431–445.

Hopkins, B.G., Jolley, V.D., and Brown, J.C. (1992). Plant utilization of iron solubilized by oat phytosiderophore. J. Plant Nutr. **15**:1599–1612.

Huang, S., Yamaji, N., and Ma, J.F. (2024). Metal Transport Systems in Plants. Annu. Rev. Plant Biol. **75**:1–25.

Ibrahim, A., Meyrueix, R., Pouliquen, G., Chan, Y.P., and Cottet, H. (2013). Size and charge characterization of polymeric drug delivery systems by Taylor dispersion analysis and capillary electrophoresis. Anal. Bioanal. Chem. **405**:5369–5379.

Kai, K., Mizutani, M., Kawamura, N., Yamamoto, R., Tamai, M., Yamaguchi, H., Sakata, K., and Shimizu, B.-I. (2008). Scopoletin is biosynthesized via ortho-hydroxylation of feruloyl CoA by a 2-oxoglutarate-dependent dioxygenase in Arabidopsis thaliana. Plant J. **55**:989–999.

Kamal, K., Hagagg, L., and Awad, F. (2000). Improved Fe and Zn acquisition by guava seedlings grown in calcareous soils intercropped with graminaceous species. J. Plant Nutr. **23**:2071–2080.

Kang, J., Park, J., Choi, H., Burla, B., Kretzschmar, T., Lee, Y., and Martinoia, E. (2011). Plant ABC Transporters. Arabidopsis Book **9**: e0153.

Kang, K., Schenkeveld, W.D.C., Weber, G., and Kraemer, S.M. (2023). Stability of Coumarins and Determination of the Net Iron Oxidation State of Iron-Coumarin Complexes: Implications for Examining Plant Iron Acquisition Mechanisms. ACS Earth Space Chem. **7**:2339–2352.

Kim, S.A., LaCroix, I.S., Gerber, S.A., and Guerinot, M.L. (2019). The iron deficiency response in Arabidopsis thaliana requires the phosphorylated transcription factor URI. Proc. Natl. Acad. Sci. USA **116**:24933–24942.

Kobayashi, T., Itai, R.N., Aung, M.S., Senoura, T., Nakanishi, H., and Nishizawa, N.K. (2012). The rice transcription factor IDEF1 directly binds to iron and other divalent metals for sensing cellular iron status. Plant J. **69**:81–91.

Koyyappurath, S., Conéjéro, G., Dijoux, J.B., Lapeyre-Montès, F., Jade, K., Chiroleu, F., Gatineau, F., Verdeil, J.L., Besse, P., and Grisoni, M. (2015). Differential Responses of Vanilla Accessions to Root Rot and Colonization by Fusarium oxysporum f. sp. radicis-vanillae. Front. Plant Sci. **6**:1125.

Le Jean, M., Schikora, A., Mari, S., Briat, J.-F., and Curie, C. (2005). A loss-of-function mutation in AtYSL1 reveals its role in iron and nicotianamine seed loading. Plant J. **44**:769–782.

Limmer, M.A., and Burken, J.G. (2014). Plant Translocation of Organic Compounds: Molecular and Physicochemical Predictors. Environ. Sci. Technol. Lett. **1**:156–161.

Lindsay, W.L. (1997). Chemical Equilibria in Soils (New York, USA: John Wiley & Sons).

Ling, H.-Q., Bauer, P., Bereczky, Z., Keller, B., and Ganal, M. (2002). The tomato *fer* gene encoding a bHLH protein controls iron-uptake responses in roots. Proc. Natl. Acad. Sci. USA **99**:13938–13943.

Liu, Q., Kawai, T., Inukai, Y., Aoki, D., Feng, Z., Xiao, Y., Fukushima, K., Lin, X., Shi, W., Busch, W., et al. (2023). A lignin-derived material improves plant nutrient bioavailability and growth through its metal chelating capacity. Nat. Commun. **14**:4866.

Meda, A.R., Scheuermann, E.B., Prechsl, U.E., Erenoglu, B., Schaaf, G., Hayen, H., Weber, G., and von Wirén, N. (2007). Iron Acquisition by Phytosiderophores Contributes to Cadmium Tolerance. Plant Physiol. **143**:1761–1773.

Mercer, D.K., Robertson, J., Wright, K., Miller, L., Smith, S., Stewart, C.S., and O′Neil, D.A. (2013). A Prodrug Approach to the Use of Coumarins as Potential Therapeutics for Superficial Mycoses. PLoS One **8**:e80760.

Murgia, I., Tarantino, D., Soave, C., and Morandini, P. (2011). Arabidopsis CYP82C4 expression is dependent on Fe availability and circadian rhythm, and correlates with genes involved in the early Fe deficiency response. J. Plant Physiol. **168**:894–902.

Ohshima, H. (2001). Approximate Analytic Expression for the Electrophoretic Mobility of a Spherical Colloidal Particle. J. Colloid Interface Sci. **239**:587–590.

Ortega, A., Amorós, D., and García de la Torre, J. (2011). Prediction of Hydrodynamic and Other Solution Properties of Rigid Proteins from Atomic- and Residue-Level Models. Biophys. J. **101**:892–898.

Paffrath, V., Tandron Moya, Y.A., Weber, G., von Wirén, N., and Giehl, R.F.H. (2024). A major role of coumarin-dependent ferric iron reduction in strategy I-type iron acquisition in Arabidopsis. Plant Cell **36**:642–664.

Rajniak, J., Giehl, R.F.H., Chang, E., Murgia, I., von Wirén, N., and Sattely, E.S. (2018). Biosynthesis of redox-active metabolites in response to iron deficiency in plants. Nat. Chem. Biol. **14**:442–450.

Robe, K., Conejero, G., Gao, F., Lefebvre-Legendre, L., Sylvestre-Gonon, E., Rofidal, V., Hem, S., Rouhier, N., Barberon, M., Hecker, A., et al. (2021a). Coumarin accumulation and trafficking in Arabidopsis thaliana: a complex and dynamic process. New Phytol. **229**:2062–2079.

Robe, K., Izquierdo, E., Vignols, F., Rouached, H., and Dubos, C. (2021b). The Coumarins: Secondary Metabolites Playing a Primary Role in Plant Nutrition and Health. Trends Plant Sci. **26**:248–259.

Robinson, N.J., Procter, C.M., Connolly, E.L., and Guerinot, M.L. (1999). A ferric-chelate reductase for iron uptake from soils. Nature **397**:694–697.

Rodríguez-Celma, J., Lin, W.-D., Fu, G.-M., Abadía, J., López-Millán, A.-F., and Schmidt, W. (2013). Mutually Exclusive Alterations in Secondary Metabolism Are Critical for the Uptake of Insoluble Iron Compounds by Arabidopsis and Medicago truncatula. Plant Physiol. **162**:1473–1485.

Rodríguez-Celma, J., and Schmidt, W. (2013). Reduction-based iron uptake revisited. Plant Signal. Behav. **8**:e26116.

Römheld, V., and Marschner, H. (1983). Mechanism of Iron Uptake by Peanut Plants: I. Fe reduction, chelate splitting, and release of phenolics. Plant Physiol. **71**:949–954.

Schmid, N.B., Giehl, R.F.H., Döll, S., Mock, H.-P., Strehmel, N., Scheel, D., Kong, X., Hider, R.C., and von Wirén, N. (2014). Feruloyl-CoA 6′-Hydroxylase1-Dependent Coumarins Mediate Iron Acquisition from Alkaline Substrates in Arabidopsis. Plant Physiol. **164**:160–172.

Schmidt, H., Günther, C., Weber, M., Spörlein, C., Loscher, S., Böttcher, C., Schobert, R., and Clemens, S. (2014). Metabolome Analysis of Arabidopsis thaliana Roots Identifies a Key Metabolic Pathway for Iron Acquisition. PLoS One **9**:e102444.

Selmar, D., Radwan, A., and Nowak, M. (2015). Horizontal Natural Product Transfer: A so far Unconsidered Source of Contamination of Plant-Derived Commodities. J. Environ. Anal. Toxicol. **5**:1–3.

Sisó-Terraza, P., Luis-Villarroya, A., Fourcroy, P., Briat, J.-F., Abadía, A., Gaymard, F., Abadía, J., and Álvarez-Fernández, A. (2016). Accumulation and Secretion of Coumarinolignans and other Coumarins in Arabidopsis thaliana Roots in Response to Iron Deficiency at High pH. Front. Plant Sci. **7**:1711.

Siwinska, J., Siatkowska, K., Olry, A., Grosjean, J., Hehn, A., Bourgaud, F., Meharg, A.A., Carey, M., Lojkowska, E., and Ihnatowicz, A. (2018). Scopoletin 8-hydroxylase: a novel enzyme involved in coumarin biosynthesis and iron-deficiency responses in Arabidopsis. J. Exp. Bot. **69**:1735–1748.

Schaaf, G., Ludewig, U., Erenoglu, B.E., Mori, S., Kitahara, T., and von Wirén, N. (2004). ZmYS1 Functions as a Proton-coupled Symporter for Phytosiderophore- and Nicotianamine-chelated Metals. J. Biol. Chem. **279**:9091–9096.

Talamond, P., Verdeil, J.-L., and Conéjéro, G. (2015). Secondary metabolite localization by autofluorescence in living plant cells. Molecules **20**:5024–5037.

Terés, J., Busoms, S., Perez Martín, L., Luís-Villarroya, A., Flis, P., Álvarez-Fernández, A., Tolrà, R., Salt, D.E., and Poschenrieder, C. (2019). Soil carbonate drives local adaptation in Arabidopsis thaliana. Plant Cell Environ. **42**:2384–2398.

Tipton, C.L., and Buell, E.L. (1970). Ferric iron complexes of hydroxamic acids from maize. Phytochem **9**:1215–1217.

Tsai, H.H., Rodríguez-Celma, J., Lan, P., Wu, Y.C., Vélez-Bermúdez, I.C., and Schmidt, W. (2018). Scopoletin 8-Hydroxylase-Mediated Fraxetin Production Is Crucial for Iron Mobilization. Plant Physiol. **177**:194–207.

Tsednee, M., Huang, Y.-C., Chen, Y.-R., and Yeh, K.-C. (2016). Identification of metal species by ESI-MS/MS through release of free metals from the corresponding metal-ligand complexes. Sci. Rep. **6**:26785.

Vanholme, R., Sundin, L., Seetso, K.C., Kim, H., Liu, X., Li, J., De Meester, B., Hoengenaert, L., Goeminne, G., Morreel, K., et al. (2019). COSY catalyses trans – cis isomerization and lactonization in the biosynthesis of coumarins. Nat. Plants **5**:1066–1075.

Vansuyt, G., Robin, A., Briat, J.-F., Curie, C., and Lemanceau, P. (2007). Iron acquisition from Fe-pyoverdine by Arabidopsis thaliana. Mol. Plant Microbe Interact. **20**:441–447.

Varotto, C., Maiwald, D., Pesaresi, P., Jahns, P., Salamini, F., and Leister, D. (2002). The metal ion transporter IRT1 is necessary for iron homeostasis and efficient photosynthesis in Arabidopsis thaliana. Plant J. **31**:589–599.

Velasco-Olmo, A., Ormaetxea Gisasola, J., Martinez Galvez, J.M., Vera Lillo, J., and Shnyrova, A.V. (2019). Combining patch-clamping and fluorescence microscopy for quantitative reconstitution of cellular membrane processes with Giant Suspended Bilayers. Sci. Rep. **9**:7255.

Vert, G., Grotz, N., Dédaldéchamp, F., Gaymard, F., Guerinot, M.L., Briat, J.-F., and Curie, C. (2002). IRT1, an Arabidopsis Transporter Essential for Iron Uptake from the Soil and for Plant Growth. Plant Cell **14**:1223–1233.

Waters, B.M., Chu, H.H., Didonato, R.J., Roberts, L.A., Eisley, R.B., Lahner, B., Salt, D.E., and Walker, E.L. (2006). Mutations in Arabidopsis yellow stripe-like1 and yellow stripe-like3 reveal their roles in metal ion homeostasis and loading of metal ions in seeds. Plant Physiol. **141**:1446–1458.

Weber, G., von Wirén, N., and Hayen, H. (2006). Analysis of iron(II)/iron (III) phytosiderophore complexes by nano-electrospray ionization Fourier transform ion cyclotron resonance mass spectrometry. Rapid Commun. Mass Spectrom. **20**:973–980.

Xiong, H., Kakei, Y., Kobayashi, T., Guo, X., Nakazono, M., Takahashi, H., Nakanishi, H., Shen, H., Zhang, F., Nishizawa, N.K., et al. (2013). Molecular evidence for phytosiderophore-induced improvement of iron nutrition of peanut intercropped with maize in calcareous soil. Plant Cell Environ. **36**:1888–1902.

Xuan, Y., Scheuermann, E.B., Meda, A.R., Hayen, H., von Wirén, N., and Weber, G. (2006). Separation and identification of phytosiderophores and their metal complexes in plants by zwitterionic hydrophilic interaction liquid chromatography coupled to electrospray ionization mass spectrometry. J. Chromatogr. A **1136**:73–81.

Ziegler, J., Schmidt, S., Strehmel, N., Scheel, D., and Abel, S. (2017). Arabidopsis Transporter ABCG37/PDR9 contributes primarily highly oxygenated Coumarins to Root Exudation. Sci. Rep. **7**:3704.

Plant Communications, Volume *6*

Supplemental information

Coumarin-facilitated iron transport: An IRT1-independent strategy for iron acquisition in *Arabidopsis thaliana*

Kevin Robe, Max J.J. Stassen, Shunsuke Watanabe, Javier Espadas, Philippe Gonzalez, Alice Rossille, Meijie Li, Sonia Hem, Aurélien Roux, Véronique Santoni, Joseph Chamieh, Christian Dubos, and Esther Izquierdo

Supplemental information

Supplemental items

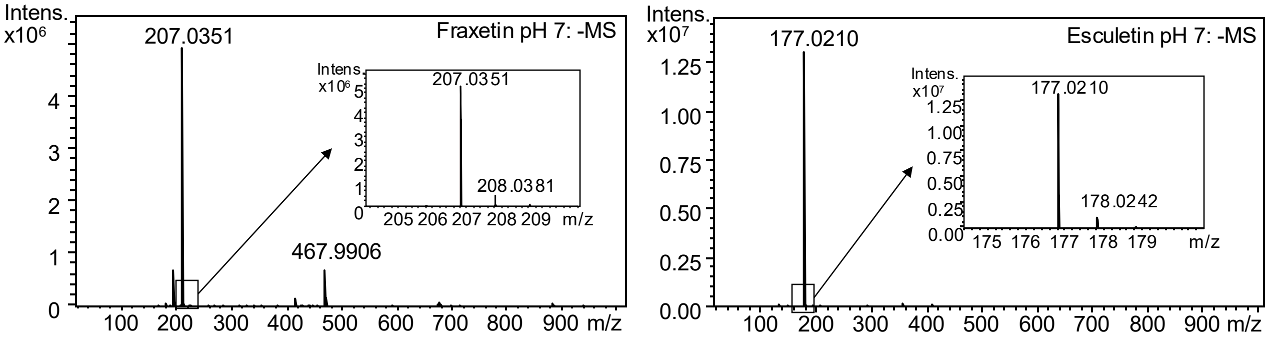

Figure S1. Direct infusion ESI-QTOF MS analysis of fraxetin (left) and esculetin (right) at pH 7 without any Fe addition. Note that [Fe(fraxetin)$_2$-4H$^+$]$^-$ can be detected even in the absence of an external Fe source, certainly due to Fe contamination.

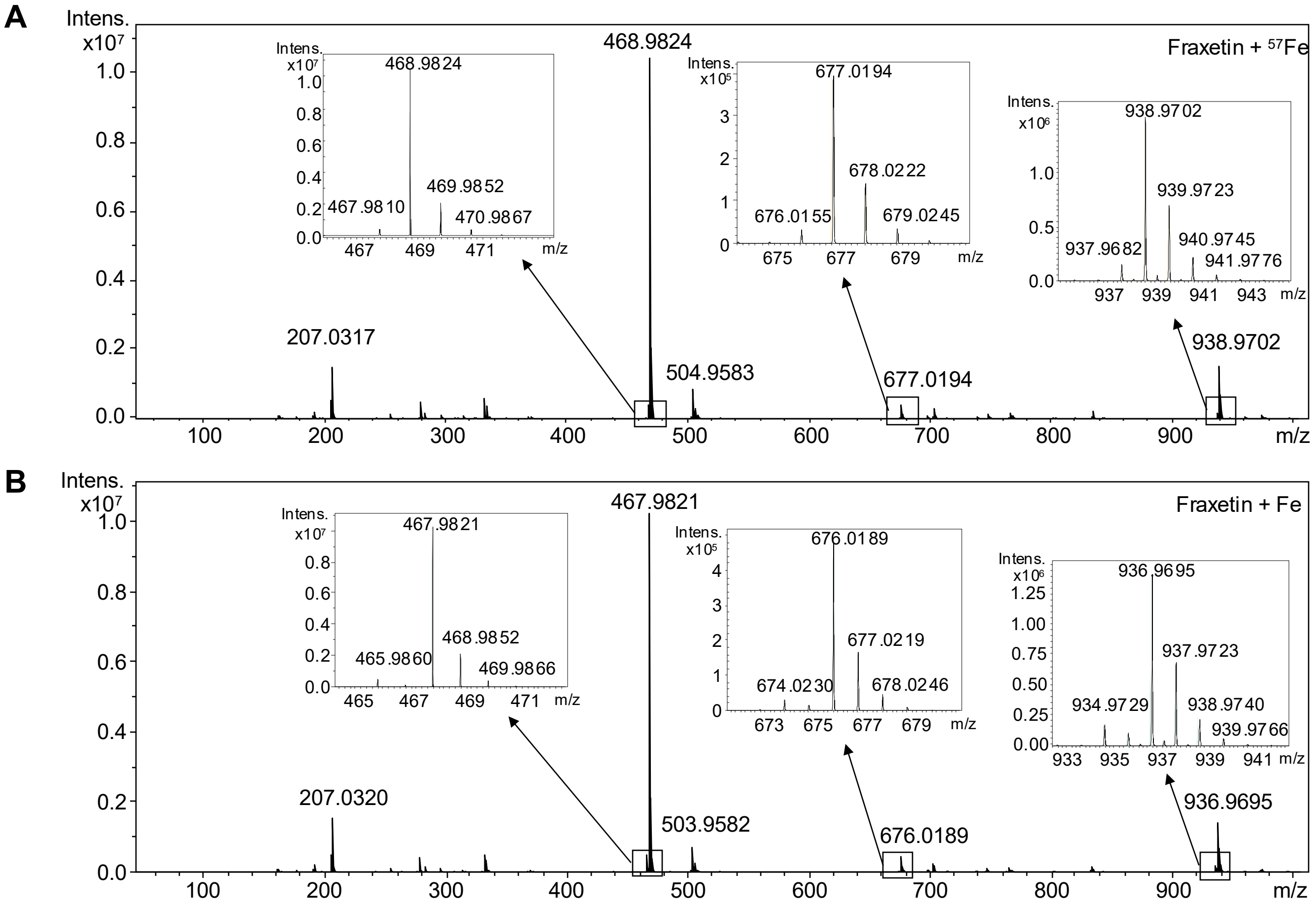

Figure S2. Analysis of isotopically labelled ^{57}Fe-fraxetin complexes.
(A) Direct infusion ESI-QTOF MS analysis of a 2:1 (mol:mol) mixture of synthetic fraxetin and $^{57}Fe^{3+}$ at pH 7. **(B)** Direct infusion ESI-QTOF MS analysis of a 2:1 (mol:mol) mixture of synthetic fraxetin and Fe^{3+} at pH 7. $^{57}Fe^{3+}$ and Fe^{3+} were obtained by acid hydrolysis of respectively $^{57}Fe_2O_3$ and Fe_2O_3. Black boxes show a zoom on the MS signal of the main coumarin species. MS intensity was measured in counts. All spectra were obtained in negative ESI mode with Q-TOF Maxis Impact HD (Bruker Daltonics). MS intensity was measured in counts.

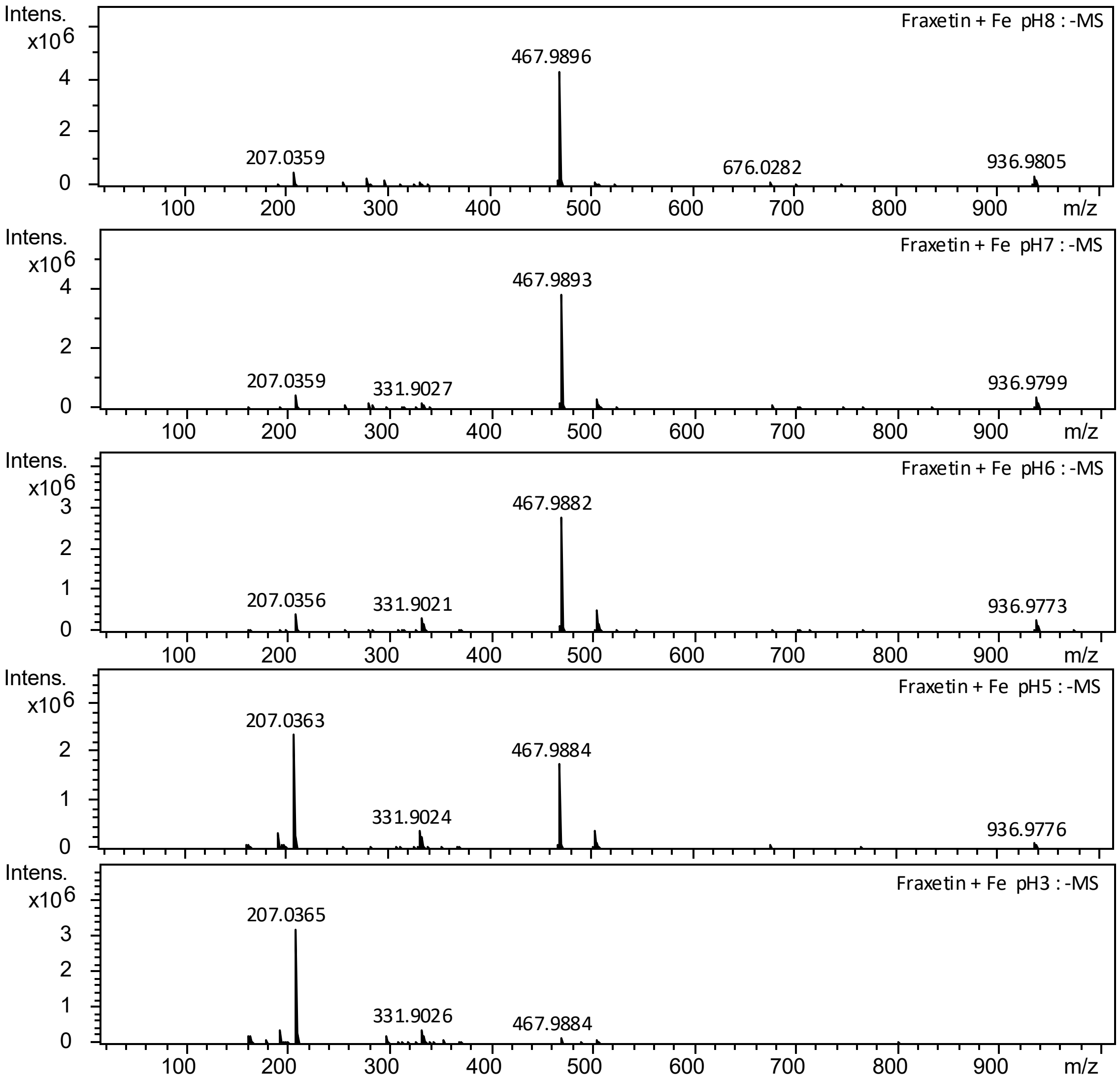

Figure S3. Analysis of the stability of Fe-fraxetin complexes at different pH. Direct infusion ESI-QTOF MS analysis. Stability of the complexes decreases when pH becomes more acidic. MS intensity was measured in counts. Note that free fraxetin signal increases in acidic conditions, confirming the dissociation of the complexes.

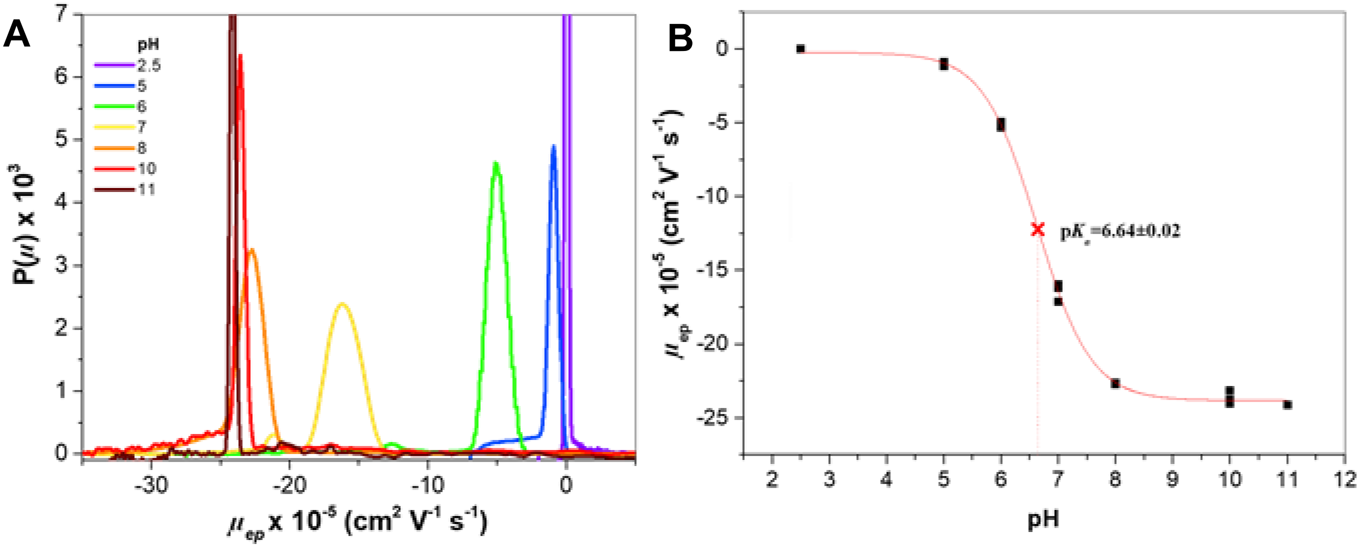

Figure S4. Determination of the pK_a of fraxetin by capillary electrophoresis.
(A) Electrophoretic mobility distributions for fraxetin as a function of pH (n=3). **(B)** Evolution of the effective mobility of fraxetin as function of pH allowing the estimation of the pK_a using equation (1) in Supplemental Method 1. Red cross represents the graphical determination of p*Ka*

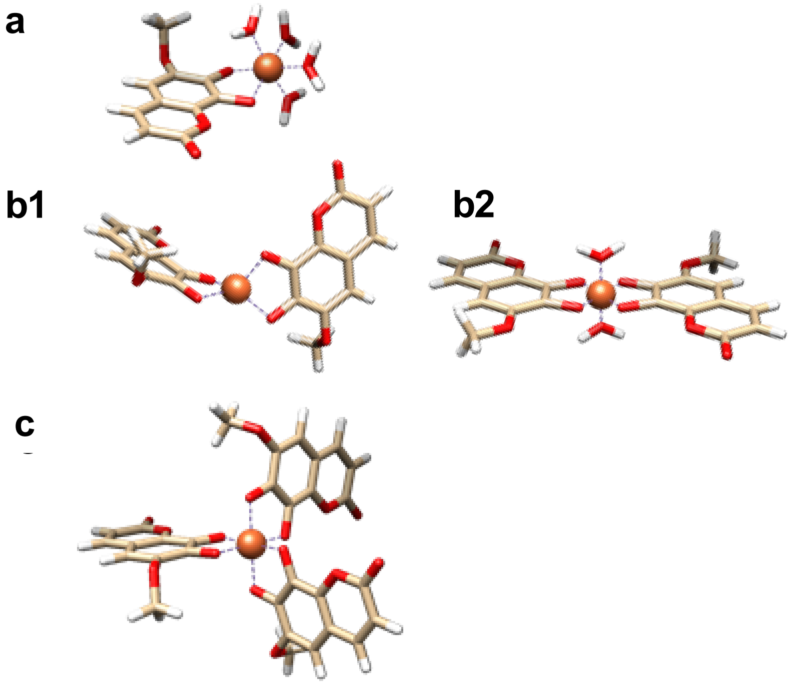

Figure S5. Proposed structures of Fe-fraxetin complexes. **a**: Fe:Fraxetin 1:1 complex, **b1**: Fe:Fraxetin 1:2 tetrahedral complex, **b2**: Fe:Fraxetin 1:2 octahedral complex; **c**: Fe:Fraxetin 1:3 octahedral complex. When needed the coordination sphere was completed with water molecules.

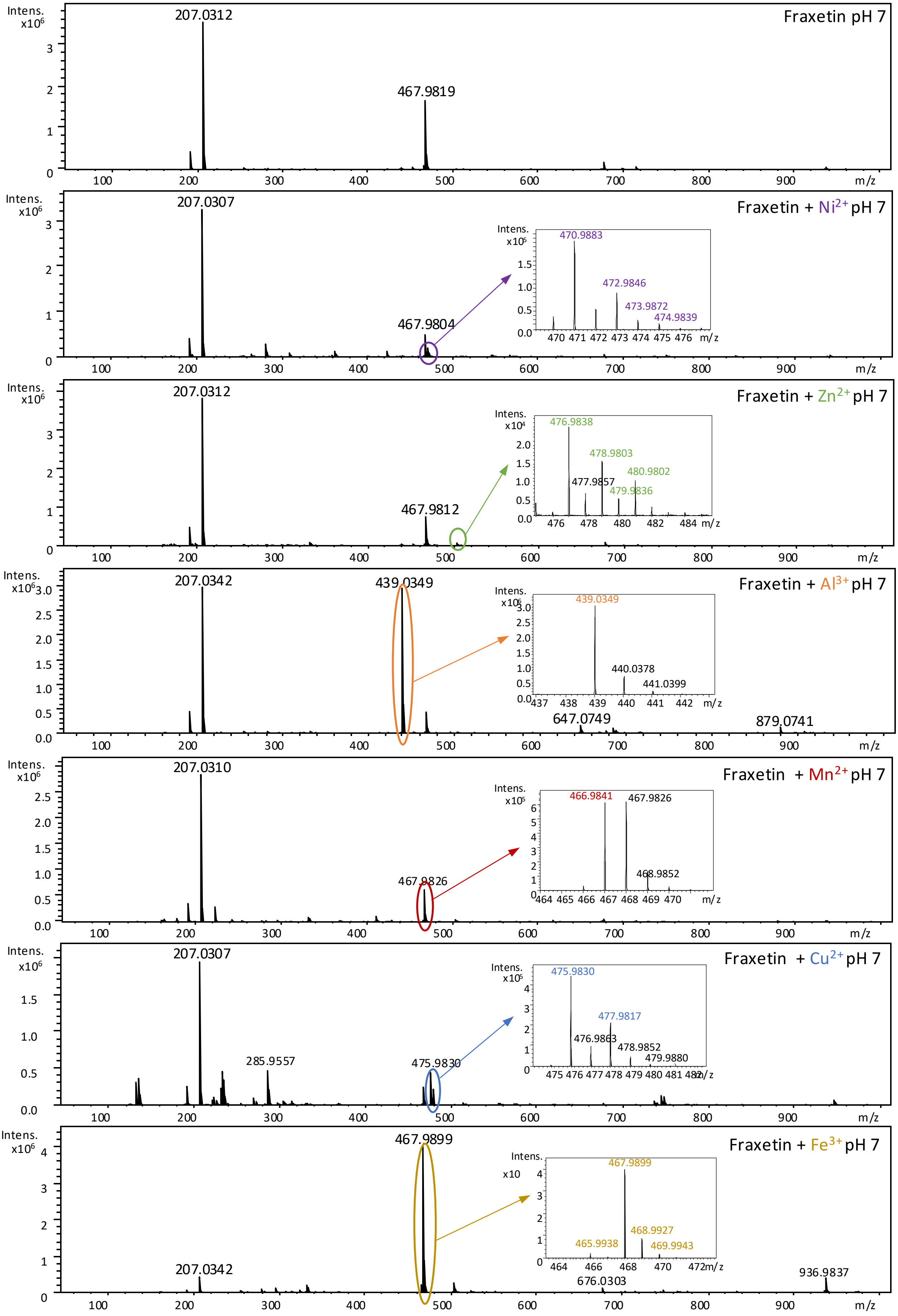

Figure S6. Identification of non-ferric metals and iron complexes with fraxetin.

Direct infusion ESI-QTOF MS analysis of a mixture of individual transition metals and synthetic fraxetin at pH 7 are presented. All tested metal solutions were prepared from chloride salts (ie: $NiCl_2$, $AlCl_3$, $MnCl_2$, $ZnCl_2$, $CuCl_2$ and $FeCl_3$). Color ellipses show a zoom of the MS signal of the formed metal-fraxetin complexes. Isotopic signatures of each metal are highlighted in colour. All spectra were obtained in negative ESI mode with Q-TOF Maxis Impact HD (Bruker Daltonics). MS intensity was measured in counts

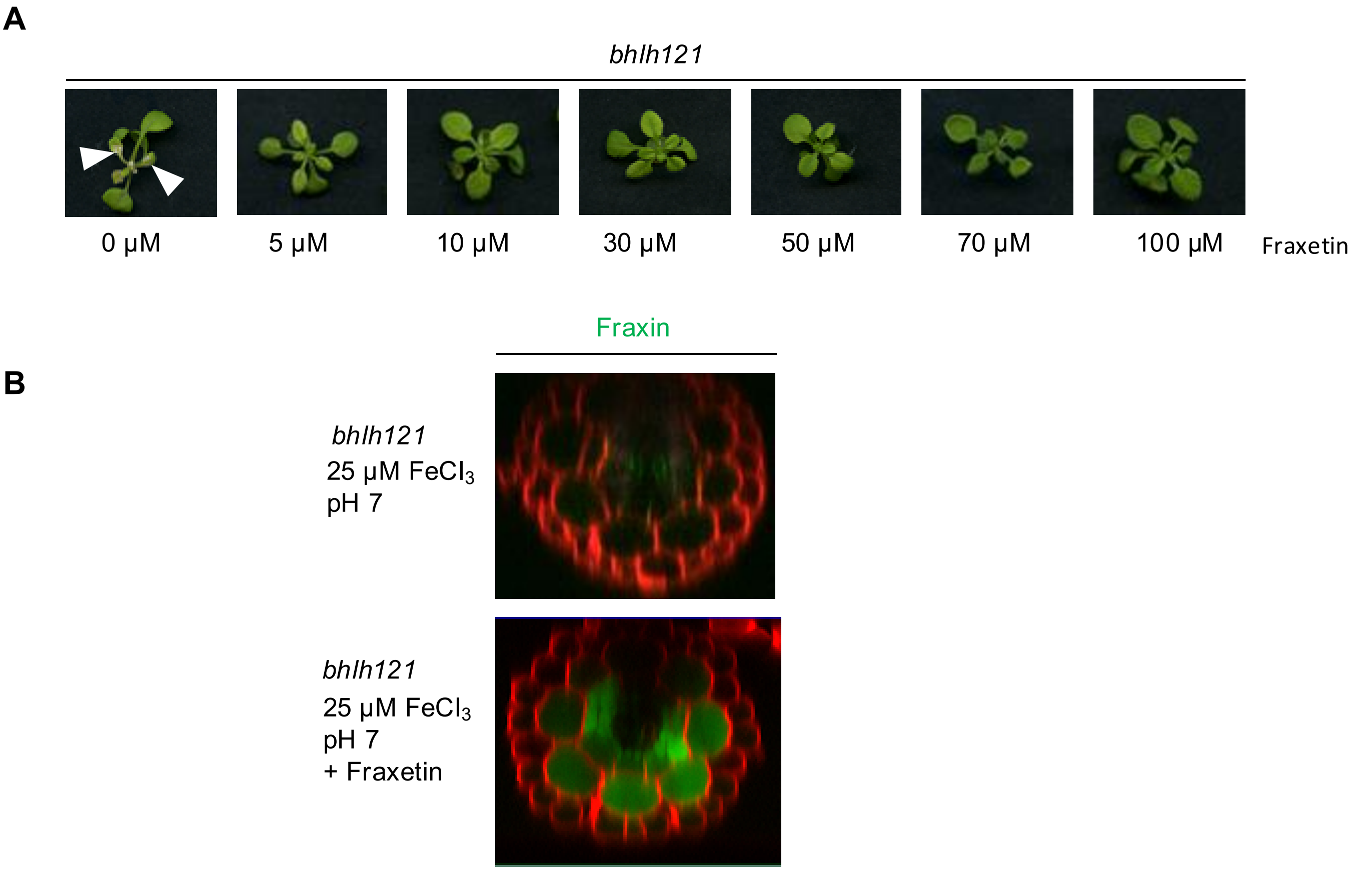

Figure S7. Phenotypic effect of fraxetin application on *bhlh121* mutant.
(A) Dose dependent phenotype complementation of *bhlh121* mutant grown with fraxetin. Fraxetin was added at different concentrations in Hoagland medium containing 25 μM $FeCl_3$ at pH 7. **(B)** Spectral deconvolution image of fraxin (green) and PI (red) in *bhlh121* mutant grown in the presence of fraxetin.

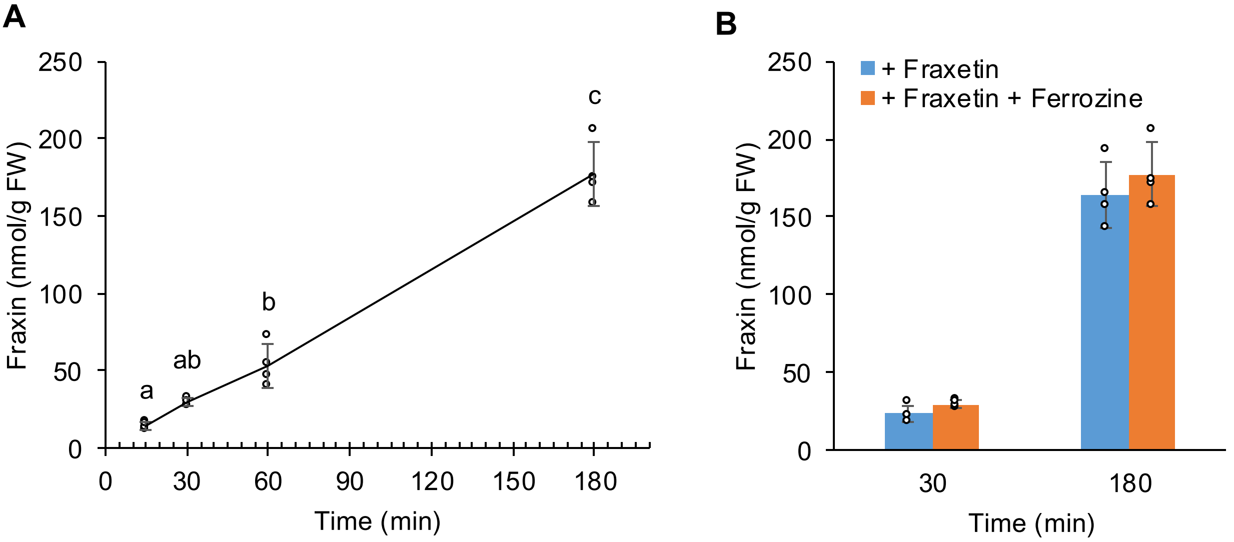

Figure S8. Fraxetin uptake in the presence of ferrozine.
(A) Three hours kinetic analysis of fraxetin uptake in *f6'h1* mutant grown in Hoagland plates containing 25 μM $FeCl_3$ at pH 7, 75μM ferrozine and 50 μM exogenous fraxetin. Time course root samples were collected and fraxin was analysed by HPLC. Means with the same letter are not significantly different according to one-way ANOVA followed by post hoc Tukey test, $P < 0.05$ (n = 4 pools of at least 50 seedlings each). Black lines are used to connect the means **(B)** Comparison of fraxetin uptake in the presence and in the absence of ferrozine at two time points (30 min and 180 min) of the kinetic study showed in (A). *t* test significant difference: * $P < 0.05$ (n = 4 biological repeats).

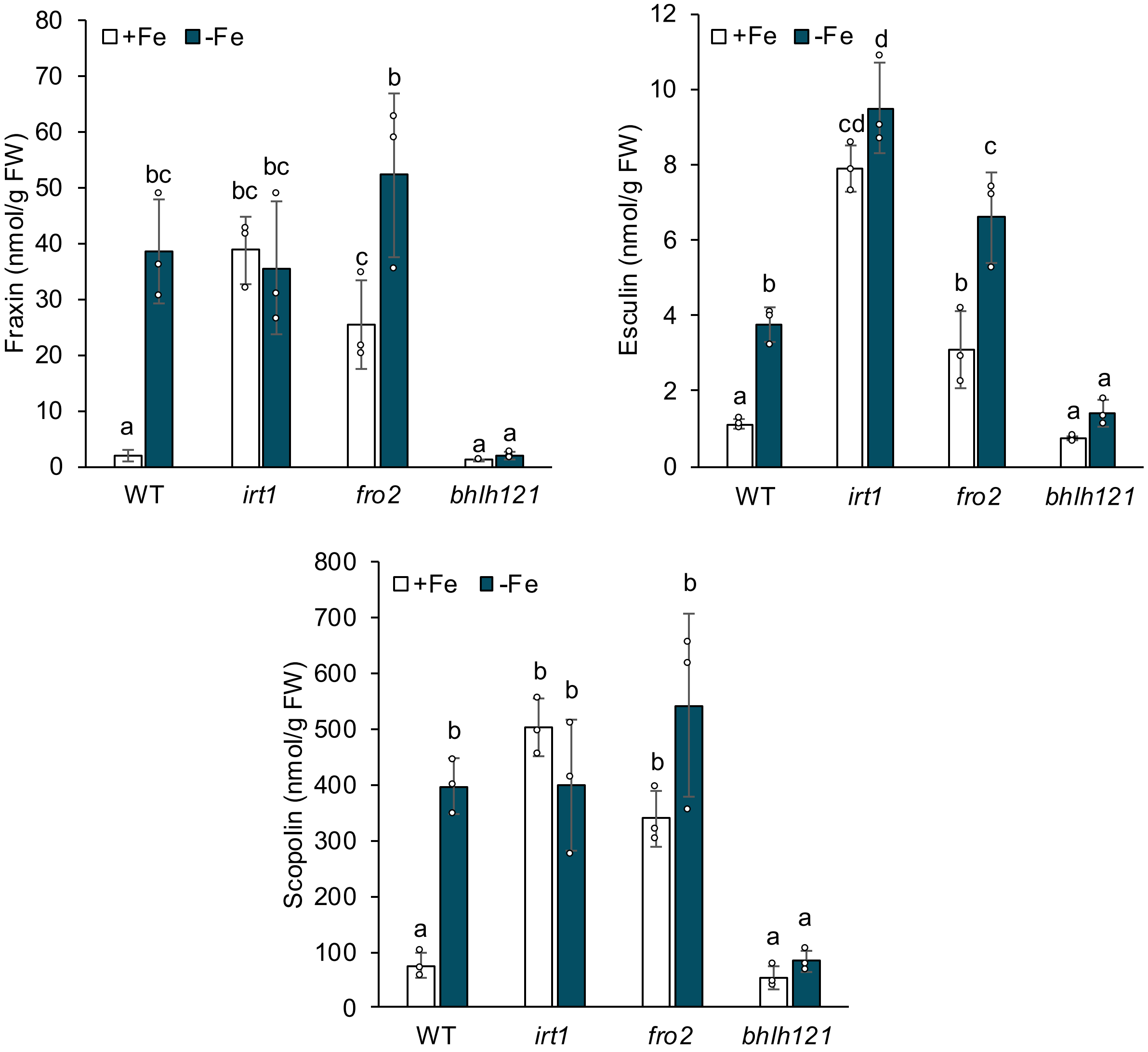

Figure S9. Coumarins accumulation in roots of iron acquisition mutants.
Analysis of fraxin, esculin and scopolin in roots of plants grown in Fe sufficient conditions (50 µM Fe-EDTA, pH 5.5) and Fe deficient conditions (0 µM Fe, pH 7). Means with the same letter are not significantly different according to one-way ANOVA followed by post hoc Tukey test, $P < 0.05$ (n = 3 biological repeats). Bars represent means ± SD.

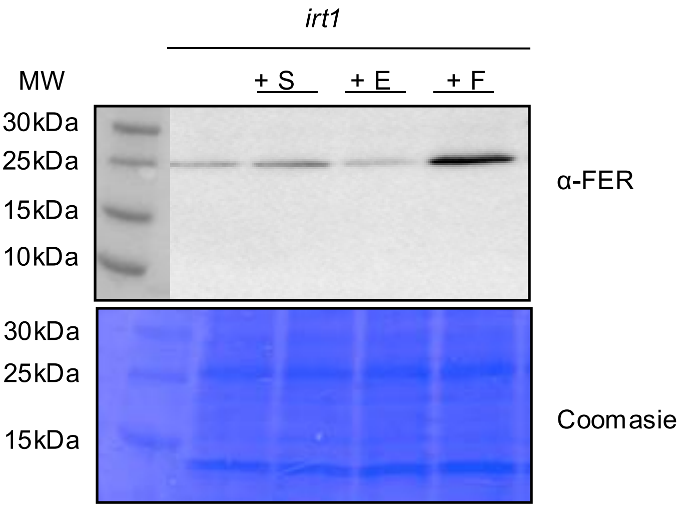

Figure S10. Western Blot of ferritins accumulation in *irt1* mutant supplemented or not with coumarins.
Ferritins accumulation in shoots of 10-day-old *irt1* mutants grown on medium containing 25 µM $FeCl_3$ and supplemented with scopoletin (+S), esculetin (+E) or fraxetin (+F). Each loaded sample was obtained by pooling approximately 10 seedling shoots. MW markers were imaged with white light and ferritins with chemiluminescence on the same membrane. Coomasie is shown as loading control.

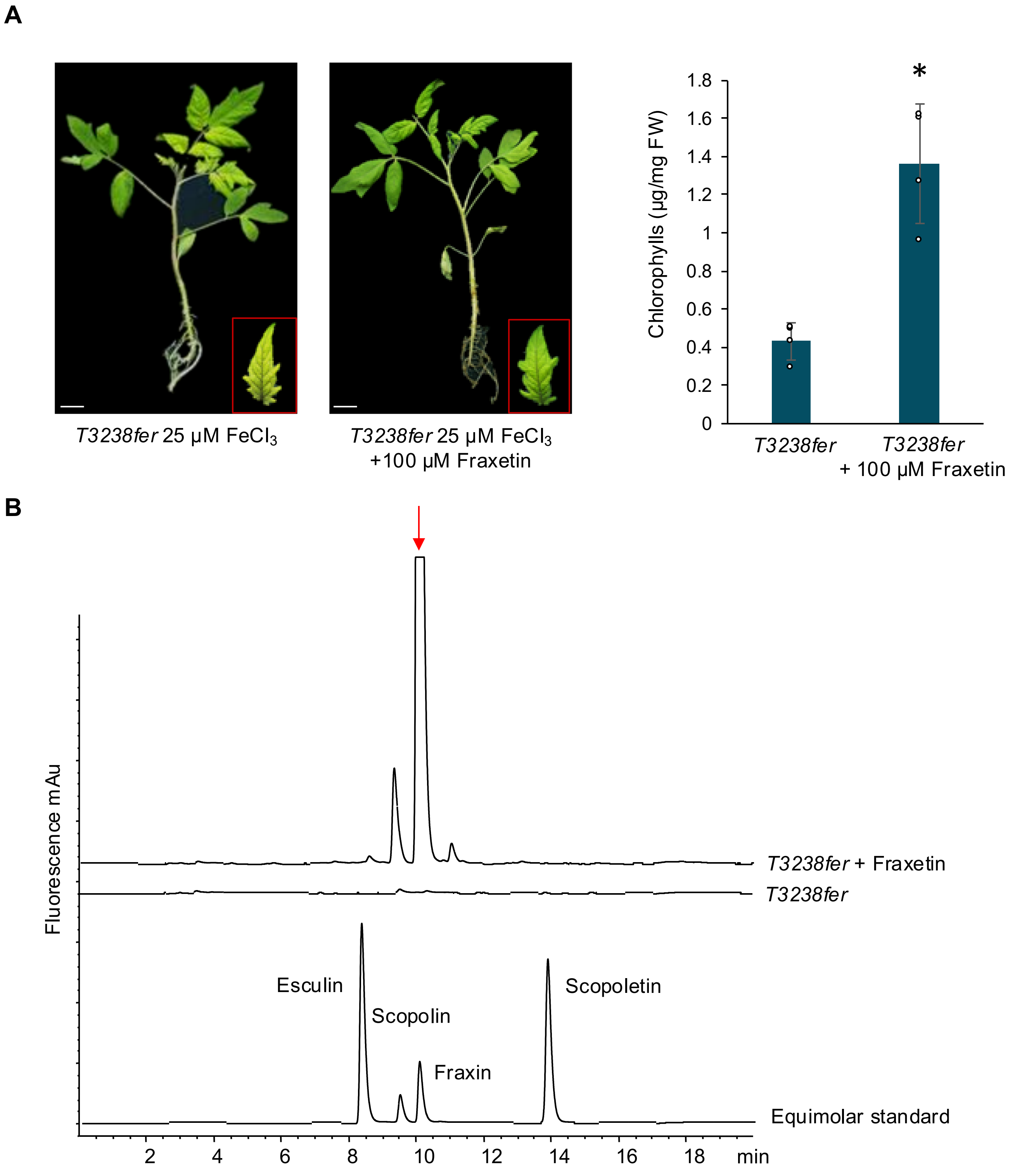

Figure S11. Effect of fraxetin on tomato *T3238fer* mutant.
(A) Complementation of *T3238fer* tomato (*Solanum lycopersicum*) mutant with fraxetin. *T3238fer* mutant was grown for 2 weeks on control Hoagland medium (pH 5.5) and then transferred to Hoagland medium containing 25 µM $FeCl_3$ at pH 7 and supplemented or not with 100 µM Fraxetin for 1 week. Central leaves were used for chlorophyll measurements. *t*-test significant difference: *P < 0.05 (n = 4 biological repeats). Bars represent means ± SD. **(B)** Representative fluorescence chromatograms obtained using λ_{exc} 365 and λ_{em} 460 nm for root extracts of *T3238fer* mutant grown as descibed in (A). The red arrow shows fraxin peak.

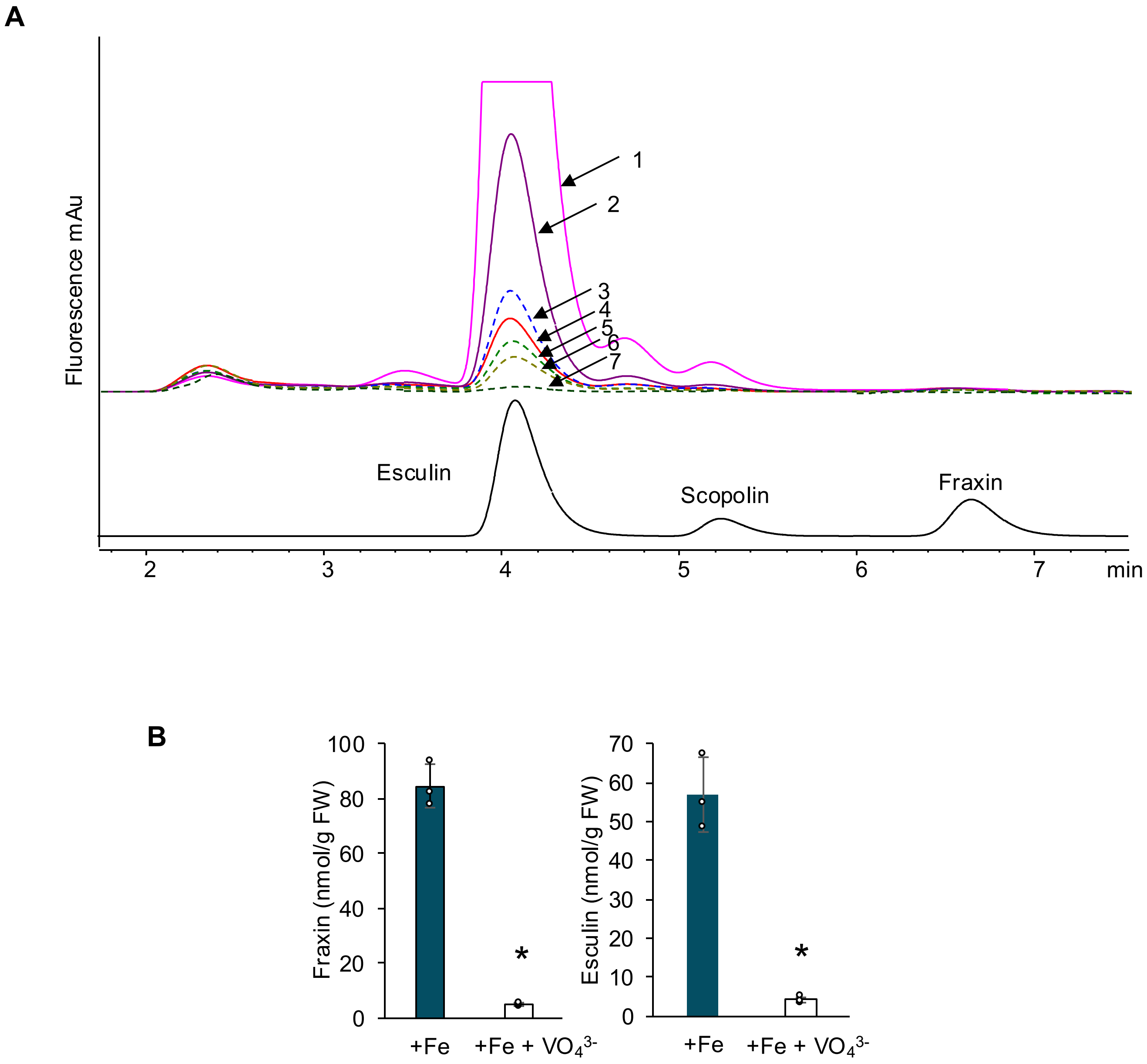

Figure S12 : **Inhibition of coumarin uptake by orthovanadate (VO_4^{3-}) addition.**
(A) Representative fluorescence chromatograms obtained using λ_{exc} 365 and λ_{em} 460 nm for root extracts of Arabidopsis *f6'h1* mutant seedlings grown for 7 days on Hoagland agar plates and then transferred 7 days on Hoagland agar containing poorly available Fe (25 µM $FeCl_3$) and supplemented with 50µM esculetin and different concentrations of orthovanadate (VO_4^{3-}). 1 : + 0 µM VO_4^{3-} , 2 : + 50µM VO_4^{3-}, 3: + 100 µM VO_4^{3-}, 4 : + 200 µM VO_4^{3-}, 5 : + 250 µM VO_4^{3-}, 6: + 500 µM VO_4^{3-}, 7: + 1 mM VO_4^{3-}. **(B)** Uptake of coumarins by roots grown in hydroponic Hoagland solution containing poorly available Fe (+Fe, 25 µM $FeCl_3$), supplemented or not with 50 µM orthovanadate (VO_4^{3-}). Plants were kept for 3 days in the presence of coumarins and orthovanadate. Coumarin glucosides were analysed by HPLC. *t*-test significant difference: *P < 0.05 (n = 3 biological repeats). Bars represent means ± SD.

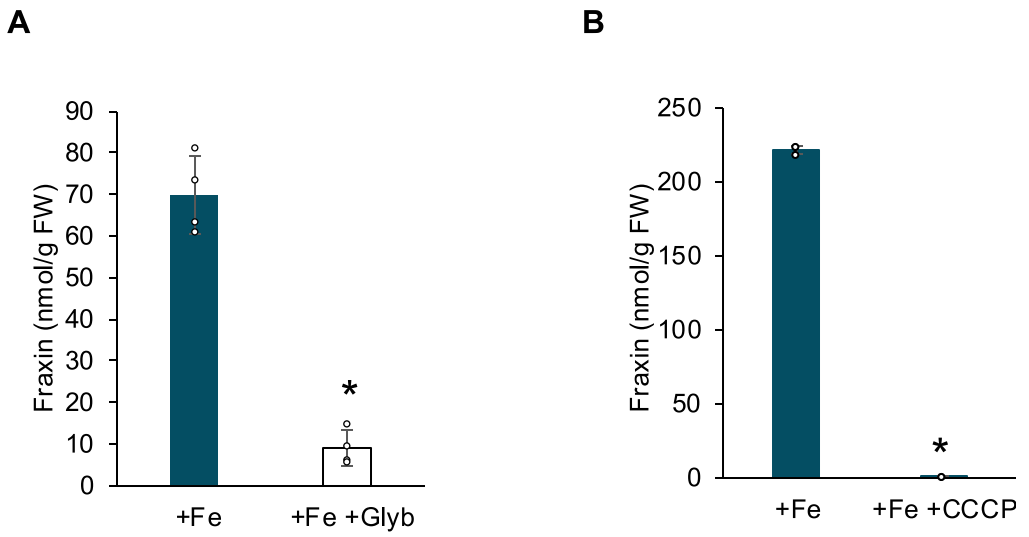

Figure S13. Inhibition of fraxetin uptake by the ATP-dependent transport inhibitor glibenclamide and the proton uncoupler CCCP.
(A) Uptake of fraxetin by roots of Arabidopsis *f6'h1* mutant seedlings grown in hydroponic Hoagland solution containing poorly available Fe (+Fe , 25 µM $FeCl_3$), supplemented or not with 150 µM glibenclamide (Glyb). Plants were kept for 3 days in the presence of fraxetin and glibenclamide and fraxin was analyzed by HPLC. **(B)** Uptake of fraxetin by roots of Arabidopsis *f6'h1* mutant seedlings grown as in (A), supplemented or not with 10 µM CCCP. Plants were kept for 3 days in the presence of fraxetin and CCCP and fraxin was analyzed by HPLC. *t* test significant difference: *P < 0.05 (*n* = 4 biological repeats). Bars represent means ± SD.

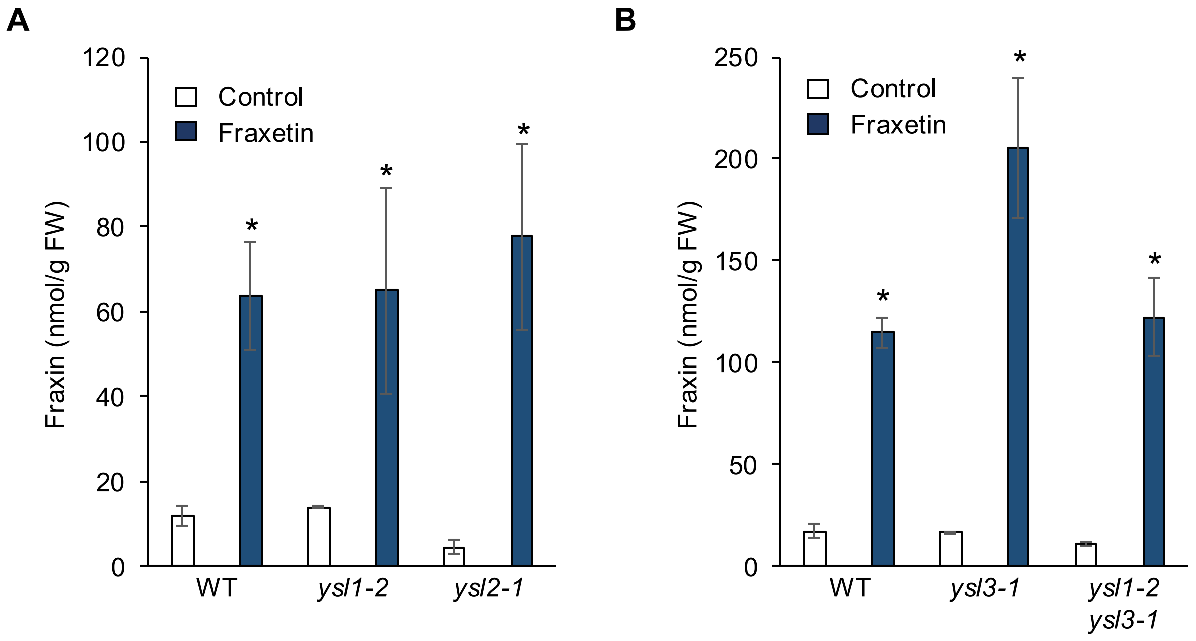

Figure S14. Fraxetin uptake is not inhibited in *ysl1*, *ysl2*, *ysl3* and *ysl1 ysl3* mutants.
(A) Uptake of fraxetin by roots of Arabidopsis wild type (WT) and *ysl1-2* and *ysl2-1* mutant seedlings grown on solid Hoagland medium containing poorly available Fe (+Fe , 25 µM $FeCl_3$) supplemented or not with 100 µM fraxetin. **(B)** Uptake of fraxetin by roots of Arabidopsis WT and *ysl3-1* and *ysl1-2 ysl3-1* mutant seedlings grown on solid Hoagland medium containing poorly available Fe (+Fe , 25 µM $FeCl_3$) supplemented or not with 100 µM fraxetin. **(A-B)** *t*-test significant difference: *P < 0.05 (n = 3 biological repeats). Bars represent means ± SD.

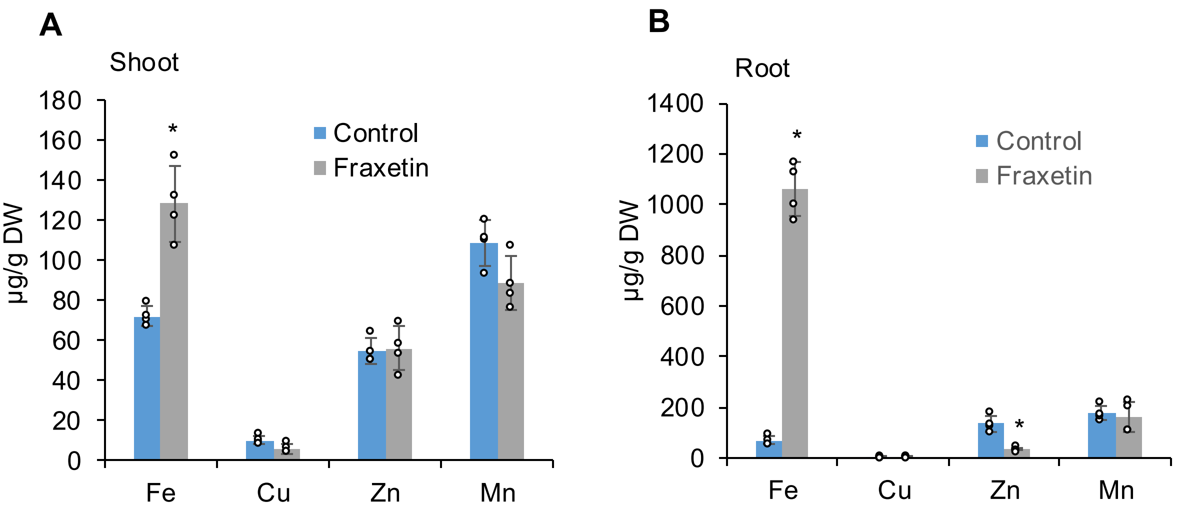

Figure S15. Effect of fraxetin treatment on the accumulation of iron in *Arabidopsis thaliana* WT plants.

(A) Shoots and **(B)** roots Fe and transition metals content of WT plants. Plants were grown for 10 days in control Hoagland agar plates and then transferred for 10 days to new plates containing poorly available Fe Hoagland medium supplemented or not with 100 µM fraxetin. *t* test significant difference: * P < 0.05 (*n* = 4 biological repeats). Bars represent means ± SD. Fe: iron, Cu: copper, Zn: zinc and Mn: manganese.

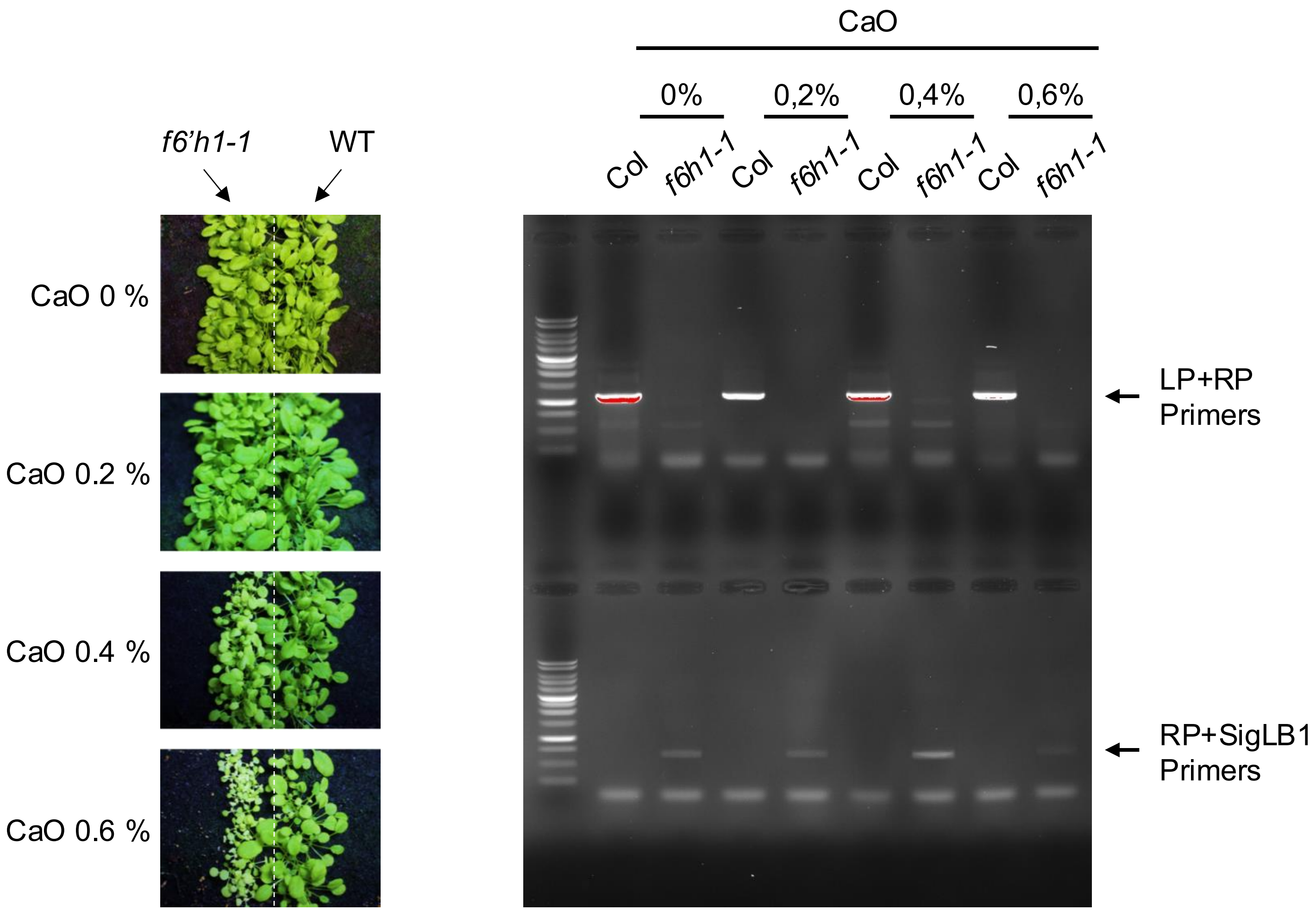

Figure S16. Genotyping of wild type (WT) and *f6'h1* mutant roots prior to coumarins LC-MS/MS analysis. The *F6'H1* wild type allele was amplified with the LP (5'-CCTGTTGCTGTGGAAGAGAAG-3') and RP (5'-GCAGATATCAGGCCAGAACTG-3') primers whereas the T-DNA insertion was assessed with the RP and SigLB1 (5'-CGGAACCACCATCAAACAG-3') primers.

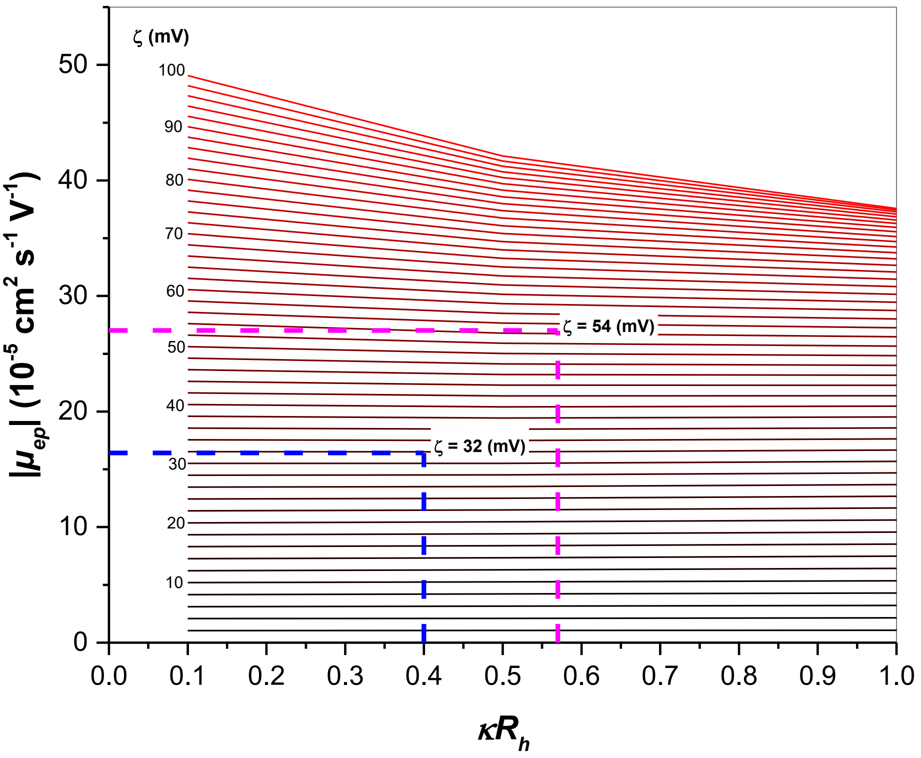

Figure S17. Graphical determination of zeta potential (ζ). ζ is obtained from the experimental effective mobility (μep) of a solute and depending on its hydrodynamic radius and on the ionic strength of the medium according to O'Brien-White-Ohshima model (OWO) (equation (2)), in a symmetrical electrolyte of NH_4HCO_3 ($m+$ = 0.189, $m-$ = 0.288). The dashed lines represent the graphical determination of ζ in a 50 mM NH_4HCO_3 buffer pH 7 for: Fraxetin (blue dashed lines, κR_h = 0.39, R_h = 0.49 nm, $|\mu_{ep}|$ = 16.42 × 10^{-5} cm^2 s^{-1} V^{-1}) and Fraxetin-Fe(III) complex (3:1) (magenta dashed lines, κR_h = 0.57, R_h = 0.77 nm, $|\mu_{ep}|$ = 27.02 × 10^{-5} cm^2 s^{-1} V^{-1})

Complex	Elemental composition	Theoretical m/z	Experimental m/z	Theoretical intensity (%)	Experimental intensity (%)	Δ intensity (%)
$[Fe(fraxetin)_2\text{-}4H^+]^-$	$C_{20}H_{12}O_{10}Fe$	465.9832	465.9843	6.4	5.2	1.2
		466.9866	466.9878	1.4	1.1	0.3
		467.9786	467.9804	100	100	0
		468.9816	468.9833	24.4	20.5	3.9
		469.9835	469.9847	5.2	4.1	1.1
$[Fe(fraxetin)_3\text{-}4H^+]^-$	$C_{30}H_{20}O_{15}Fe$	674.0204	674.0211	6.3	6.2	0.1
		675.0237	675.018	2.1	4.2	-2.1
		676.0158	676.0166	100	100	0
		677.0189	677.0198	35.5	35.4	0.1
		678.0211	678.0223	9.5	8.3	1.2

Table S1. Experimental and theoretical mass-to-charge ratios (*m/z*) and isotope abundance of 2:1 and 3:1 fraxetin-Fe complexes detected by Direct infusion ESI-QTOF MS analysis.
Theoretical isotope abundance was calculated using Data Analysis software from Bruker Daltonics

**Stoichiometry**	**Charge**		**R_h (nm)**	**Expected μ_{ep} ($x10^{-5}$ cm^2 V^{-1} s^{-1})**	
**Fe:Fraxetin**	**Fe^{3+}**	**Fe^{2+}**		**Fe^{3+}**	**Fe^{2+}**
1:1	+1	0	0.58[a]	+5.3	0
1:2	-1	-2	0.67[b1] / 0.72[b2]	-12.1[b1] / -10.7[b2]	-23.9[B1] / -21.9[b2]
1:3	-3	-4	0.77[c]	**-27.2**	-32.9

Table S2. Charge, hydrodynamic radius and electrophoretic mobility of Fe:Fraxetin complexes.
The charge of each complex was determined in the case of Fe(III) and Fe(II) and the hydrodynamic radius was determined on the simulated structure. Using the hydrodynamic radius and the charge, the theoretical electrophoretic mobility of each hypothetical complex could be determined using the OWO model and this latter value was compared to the experimental value of 27.02×10^{-5} cm^2 s^{-1} V^{-1} obtained from the analysis of the Fe-Fraxetin mixture. Results show that only the 3:1 was in accordance with the experimental value and thus the other structures could be discarded.

Metal	Molar masses	Isotope abundances (%)
Al	26.9815	100
Mn	54.9380	100
Fe	55.9349	92
	53.9396	6
	56.9354	2
Ni	57.9353	68
	59.9308	26
Cu	62.9296	69
	64.9278	31
Zn	63.9291	48
	65.9260	28
	67.9248	19

Table S3. Natural isotope abundances of the analyzed metals

Supplemental experimental procedures

Methods S1. Detailed protocols for pKa of Fraxetin determination and Effective charge of fraxetin and Fe(III)-fraxetin complex determination

pK_a of Fraxetin.

The determination of the acid constant of fraxetin was realised by capillary electrophoresis by analysing fraxetin at different pH values between 2.5 and 11. The calculated electrophoretic mobility, obtained from the transformation of the temporal electropherogram into an electrophoretic mobility distribution as described in (Chamieh *et al.*, 2015), was then plotted against the pH value and using the equation for a monoprotic acid (Poole *et al.*, 2004) (equation (1) below) a pK_a value could be obtained as a fitting parameter. It is noteworthy that the second acidity of fraxetin was considered weak and not observed under the operating experimental conditions.

$$\mu_{ep} = \frac{\mu_{A^-} \times 10^{-pK_a}}{10^{-pK_a} + 10^{-pH}} \tag{1}$$

Were μ_{A-} is the electrophoretic mobility of fraxetin anion obtained at the plateau of the curve.

Effective charge of fraxetin and Fe(III)-fraxetin complex.

The determination of the effective charge of fraxetin and Fe-fraxetin complexes was achieved by using the method based on the O'Brien-White-Ohshima model (OWO) (Ohshima, 2001; Ibrahim *et al.*, 2013):

$$\mu_{ep} = \frac{2\varepsilon_r \varepsilon_0 \zeta}{3\eta}\left[f_1(\kappa R_h) - \left(\frac{ze\zeta}{k_B T}\right)^2 f_3(\kappa R_h) - \frac{m_- + m_+}{2}\left(\frac{ze\zeta}{k_B T}\right)^2 f_4(\kappa R_h)\right] \tag{2}$$

Where ε_r is the relative electric permittivity of the media, ε_0 is the electric permittivity of vacuum (8.85 × 10^{-12} C^2 J^{-1} m^{-1}), ζ is the zeta potential (V), η is the viscosity of the media (Pa s), κ is the Debye–Hückel parameter (m^{-1}) which represents the reciprocal thickness of the ionic cloud, R_h is the hydrodynamic radius (m), k_B is the Boltzmann constant (1.38 × 10^{-23} J K^{-1}), T is the temperature (K), z is the charge number of the electrolyte ions, while f_1, f_3 and f_4 are functions of R_h and are given by:

$$f_1(\kappa R_h) = 1 + \frac{1}{2\left[1 + 2.5/\{\kappa R_h\}\left(1 + 2e^{-\kappa R_h}\right)\right]^3} \tag{3}$$

$$f_3(\kappa R_h) = \frac{\kappa R_h\left(\kappa R_h + 1.3e^{-0.18\kappa R_h} + 2.5\right)}{2\left(\kappa R_h + 1.2e^{-7.4\kappa R_h} + 4.8\right)^3} \tag{4}$$

$$f_4(\kappa R_h) = \frac{9\kappa R_h\left(\kappa R_h + 5.2e^{-3.9\kappa R_h} + 5.6\right)}{8\left(\kappa R_h - 1.55e^{-0.32\kappa R_h} + 6.02\right)^3} \tag{5}$$

While m_+ and m_- are dimensionless ionic drag coefficients, accessible from the limiting equivalent conductivities of the cation Λ_+^0 and the anion Λ_-^0 in the electrolyte and calculated using the following equation:

$$m_\pm = \frac{2\varepsilon_r \varepsilon_0 k_B T N_A}{3\eta z \Lambda_\pm} \tag{6}$$

The formalism suggested by Ohshima (Ohshima, 2001) requires the use of a symmetrical (z:z) electrolyte. If we consider the ammonium bicarbonate (NH_4HCO_3) buffer to be a 1:1 electrolyte with an ionic strength I = C(NH_4^+), the dimensionless ionic drag coefficients of the cations and the anions can be calculated from the corresponding limiting ion conductivities which were found to be of Λ_+^0 = 68.16 S cm^2 mol^{-1} for NH_4^+ at 25°C (calculated from data taken from (Tanaka & Hashitani, 1971) via the Einstein relationship) and Λ_-^0 = 44.71 S cm^2 mol^{-1} for HCO_3^- at 25°C (calculated from data taken from (Zeebe, 2011) via the Einstein relationship). From these limiting ion conductivities, the following ionic drag coefficients were obtained using equation (6): m_+ = 0.189 and m_- = 0.288.

This model can be applied for the effective charge determination of small ions, polyelectrolytes and nanoparticles (Ibrahim *et al.*, 2013) as long as the zeta potential ζ is lower than 100 mV and that κR_h is lower than 10 (Pyell *et al.*, 2009; Ibrahim *et al.*, 2012). Further, κ is a function of the ionic strength of the media, I (mol l^{-1}), and can be calculated using the following equation:

$$\kappa = \left(\sqrt{\frac{\varepsilon_0 \varepsilon_r k_B T}{2 N_A e^2 I 1000}} \right)^{-1} \tag{7}$$

Where N_A is the Avogadro number (6.02 × 10^{23} mol^{-1}), e is the elementary electric charge (-1.6 × 10^{-19} C) and 1000 is the conversion factor from l to m^3.

Next, analytical solutions of the OWO model (equation (2) and Supplemental Figure S16) relating the μ_{ep} to κR_h at different ζ values were plotted. Knowing, experimentally, the electrophoretic mobility μ_{ep} and the hydrodynamic radius of the analysed solute, ζ could be determined graphically and finally used for the calculation of the effective charge, z_{eff}, using the following equation (Makino & Ohshima, 2010):

$$z_{eff} = \frac{8\pi R_h^2 \varepsilon_r \varepsilon_0 \kappa k_B T}{ze^2} \sinh\left(\frac{ze\zeta}{2k_B T}\right) \times \left[1 + \frac{1}{\kappa R_h} \frac{2}{\cosh^2\left(ze\zeta/4k_B T\right)} + \frac{1}{\left(\kappa R_h\right)^2} \frac{8 \ln\left[\cosh\left(ze\zeta/4k_B T\right)\right]}{\sinh^2\left(ze\zeta/2k_B T\right)} \right]^{1/2}$$

(8)

Supplemental references

Chamieh J, Martin M, Cottet H. 2015. Quantitative analysis in capillary electrophoresis: transformation of raw electropherograms into continuous distributions. *Analytical chemistry* **87**(2): 1050-1057.

Ibrahim A, Meyrueix R, Pouliquen G, Chan YP, Cottet H. 2013. Size and charge characterization of polymeric drug delivery systems by Taylor dispersion analysis and capillary electrophoresis. *Analytical and Bioanalytical Chemistry* **405**(16): 5369-5379.

Ibrahim A, Ohshima H, Allison SA, Cottet H. 2012. Determination of effective charge of small ions, polyelectrolytes and nanoparticles by capillary electrophoresis. *Journal of Chromatography A* **1247**(0): 154-164.

Makino K, Ohshima H. 2010. Electrophoretic Mobility of a Colloidal Particle with Constant Surface Charge Density. *Langmuir* **26**(23): 18016-18019.

Ohshima H. 2001. Approximate Analytic Expression for the Electrophoretic Mobility of a Spherical Colloidal Particle. *Journal of Colloid and Interface Science* **239**(2): 587-590.

Poole SK, Patel S, Dehring K, Workman H, Poole CF. 2004. Determination of acid dissociation constants by capillary electrophoresis. *Journal of Chromatography A* **1037**(1): 445-454.

Pyell U, B□cking W, Huhn C, Herrmann B, Merkoulov A, Mannhardt J, Jungclas H, Nann T. 2009. Calibration-free concentration determination of charged colloidal nanoparticles and determination of effective charges by capillary isotachophoresis. *Analytical and Bioanalytical Chemistry* **395**(6): 1681-1691.

Tanaka K, Hashitani T. 1971. Measurements of self-diffusion coefficients of ammonium ion in aqueous solutions. *Transactions of the Faraday Society* **67**(0): 2314-2317.

Zeebe RE. 2011. On the molecular diffusion coefficients of dissolved CO2,HCO3-, and CO32- and their dependence on isotopic mass. *Geochimica et Cosmochimica Acta* **75**(9): 2483-2498.
